# Supplementary material for: A targetable dependency on nonsense-mediated decay for cellular homeostasis and immune control in small cell lung cancer
Source: Mol Cancer. 2026 Aug 27;25:210. doi: 10.1186/s12943-026-02750-2 (PMC13551691; doi:10.1186/s12943-026-02750-2)
Supplement: Supplementary file 1 [file 12943_2026_2750_MOESM1_ESM.pdf]

Figure S1

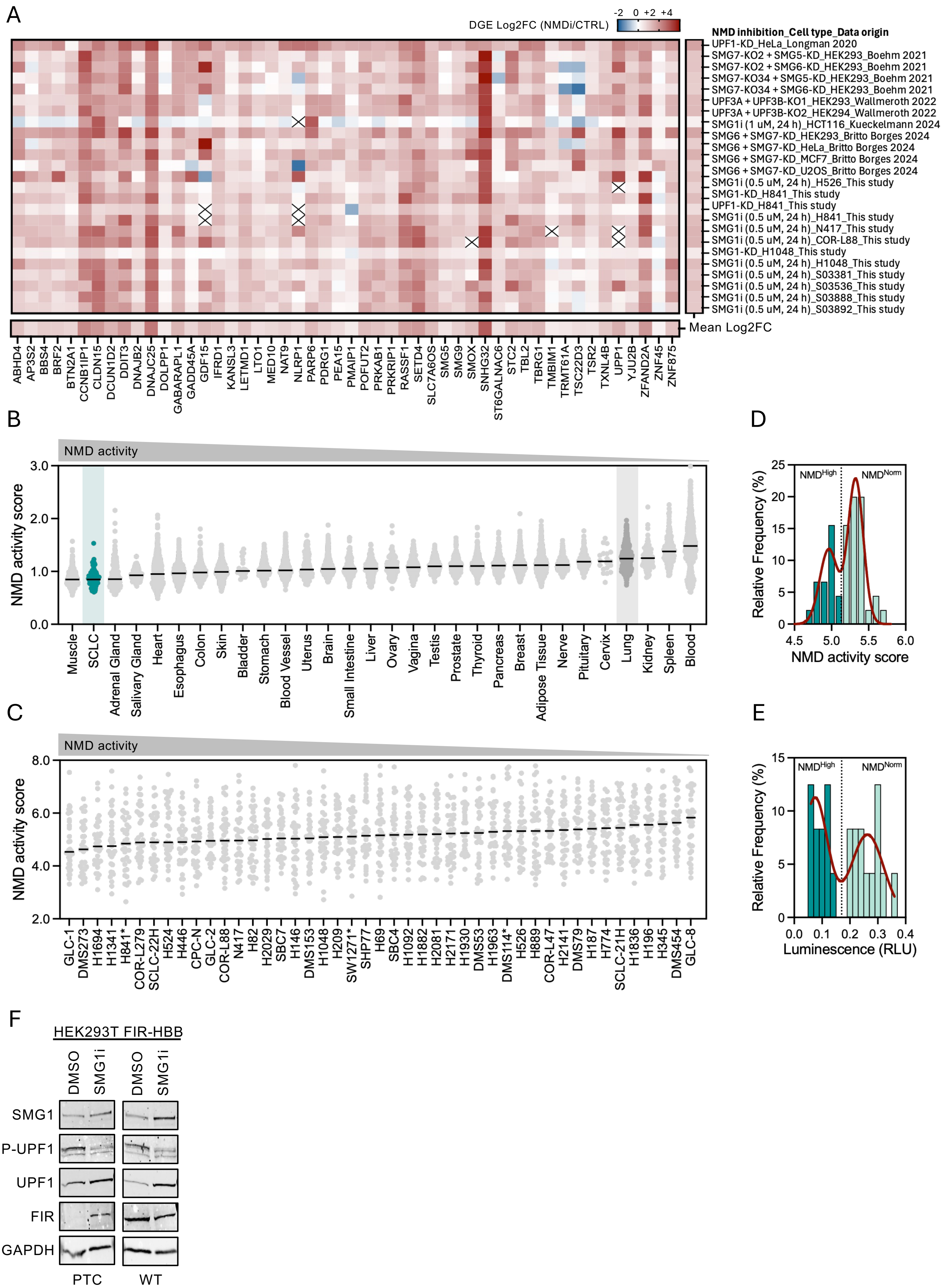
A
DGE Log2FC (NMDi/CTRL)
-2 0 +2 +4
NMD inhibition_Cell type_Data origin
UPF1-KD_HeLa_Longman 2020
SMG7-KO2 + SMG5-KD_HEK293_Boehm 2021
SMG7-KO2 + SMG6-KD_HEK293_Boehm 2021
SMG7-KO34 + SMG5-KD_HEK293_Boehm 2021
SMG7-KO34 + SMG6-KD_HEK293_Boehm 2021
UPF3A + UPF3B-KO1_HEK293_Wallmeroth 2022
UPF3A + UPF3B-KO2_HEK294_Wallmeroth 2022
SMG1i (1 uM, 24 h)_HCT116_Kueckelmann 2024
SMG6 + SMG7-KD_HEK293_Britto Borges 2024
SMG6 + SMG7-KD_HeLa_Britto Borges 2024
SMG6 + SMG7-KD_MCF7_Britto Borges 2024
SMG6 + SMG7-KD_U2OS_Britto Borges 2024
SMG1i (0.5 uM, 24 h)_H526_This study
SMG1-KD_H841_This study
UPF1-KD_H841_This study
SMG1i (0.5 uM, 24 h)_H841_This study
SMG1i (0.5 uM, 24 h)_N417_This study
SMG1i (0.5 uM, 24 h)_COR-L88_This study
SMG1-KD_H1048_This study
SMG1i (0.5 uM, 24 h)_H1048_This study
SMG1i (0.5 uM, 24 h)_S03381_This study
SMG1i (0.5 uM, 24 h)_S03536_This study
SMG1i (0.5 uM, 24 h)_S03888_This study
SMG1i (0.5 uM, 24 h)_S03892_This study
Mean Log2FC
ABHD4 AP3S2 BBS4 BRF2 BTN2A1 CCNB1IP1 CLDN15 DCUN1D2 DDIT3 DNAJB2 DNAJC25 DOLPP1 GABARAPL1 GADD45A GDF15 IFRD1 KANSL3 LETMD1 LTO1 MED10 NAT9 NLRP1 PARP6 PDRG1 PEA15 PMAIP1 POFUT2 PRKAB1 PRKRIP1 RASSF1 SETD4 SLC7A6OS SMG5 SMG9 SMOX SNHG32 ST6GALNAC6 STC2 TBL2 TBRG1 TMBIM1 TRMT61A TSC22D3 TSR2 TXNL4B UPP1 YJU2B ZFAND2A ZNF45 ZNF875
B
NMD activity
NMD activity score
3.0 2.0 1.0 0.0
Muscle SCLC Adrenal Gland Salivary Gland Heart Esophagus Colon Skin Bladder Stomach Blood Vessel Uterus Brain Small Intestine Liver Ovary Vagina Testis Prostate Thyroid Pancreas Breast Adipose Tissue Nerve Pituitary Cervix Lung Kidney Spleen Blood
C
NMD activity
NMD activity score
8.0 6.0 4.0 2.0
GLC-1 DMS273 H1694 H1341 H841* COR-L279 SCLC-22H H524 H446 CPC-N GLC-2 COR-L88 N417 H82 H2029 SBC7 H146 DMS153 H1048 H209 SW1271* SHP77 H69 SBC4 H1092 H1882 H2081 H2171 H1930 DMS53 H1963 DMS114* H526 H889 COR-L47 H2141 DMS79 H187 H774 SCLC-21H H1836 H196 H345 DMS454 GLC-8
D
NMD^High NMD^Norm
Relative Frequency (%)
25 20 15 10 5 0
4.5 5.0 5.5 6.0
NMD activity score
E
NMD^High NMD^Norm
Relative Frequency (%)
15 10 5 0
0.0 0.1 0.2 0.3 0.4
Luminescence (RLU)
F
HEK293T FIR-HBB
DMSO SMG1i DMSO SMG1i
SMG1
P-UPF1
UPF1
FIR
GAPDH
PTC WT

Figure S1 – related to Figure 1. Transcriptome-based NMD activity scores reliably estimate NMD pathway activity across multiple human tissues and cancers. A) Heatmap for log2FC expression of NMD-inhibited (NMDi) *vs* control (CTRL) samples referring to 50 NMD-sensitive genes employed to calculate transcriptome-based NMD activity scores, as proposed by Wang et al[43]. Six independent NMD inhibition studies[48–53] were included – different from the four studies previously employed by Wang et al.[44–47] –, with the reference, cell system and NMD inhibition method indicated at the right side of the heatmap. This analysis additionally includes data from this study referring to NMD inhibition by SMG1i treatment in SCLC cell lines (n=5), patient-derived tumor cells (n=4), and SMG1-KD or UPF1-KD in the SCLC cell line H1048 and the SMARCA4-UT cell line H841. **B)** Transcriptome-based NMD activity scores calculated for all adult healthy tissues within the GTEx database harmonized with published SCLC data[5], with SCLC and lung healthy tissue highlighted. **C)** Transcriptome-based NMD activity scores calculated from expression data for n = 45 human SCLC cell lines, some of which have been recently reclassified as SMARCA4-UT[63] (marked by *, see **Table S1C**). **D)** Frequency distribution for NMD activity score median values shown in **C**, with resultant histograms adjusting to two gaussian curves which reveal two different NMD activity groups. **E)** Frequency distribution for Luminescence (RLU) values derived from luciferase reporter assays (see **Fig. 1E**), with resultant histograms adjusting to two gaussian curves which reveal two different NMD activity groups. **F)** Western blot of HEK293T treated with DMSO vs SMG1i and transfected for luciferase assays shown in **Fig. 1D** with FIR-HBB-WT (WT) or FIR-HBB-PTC reporters (PTC).

Figure S2

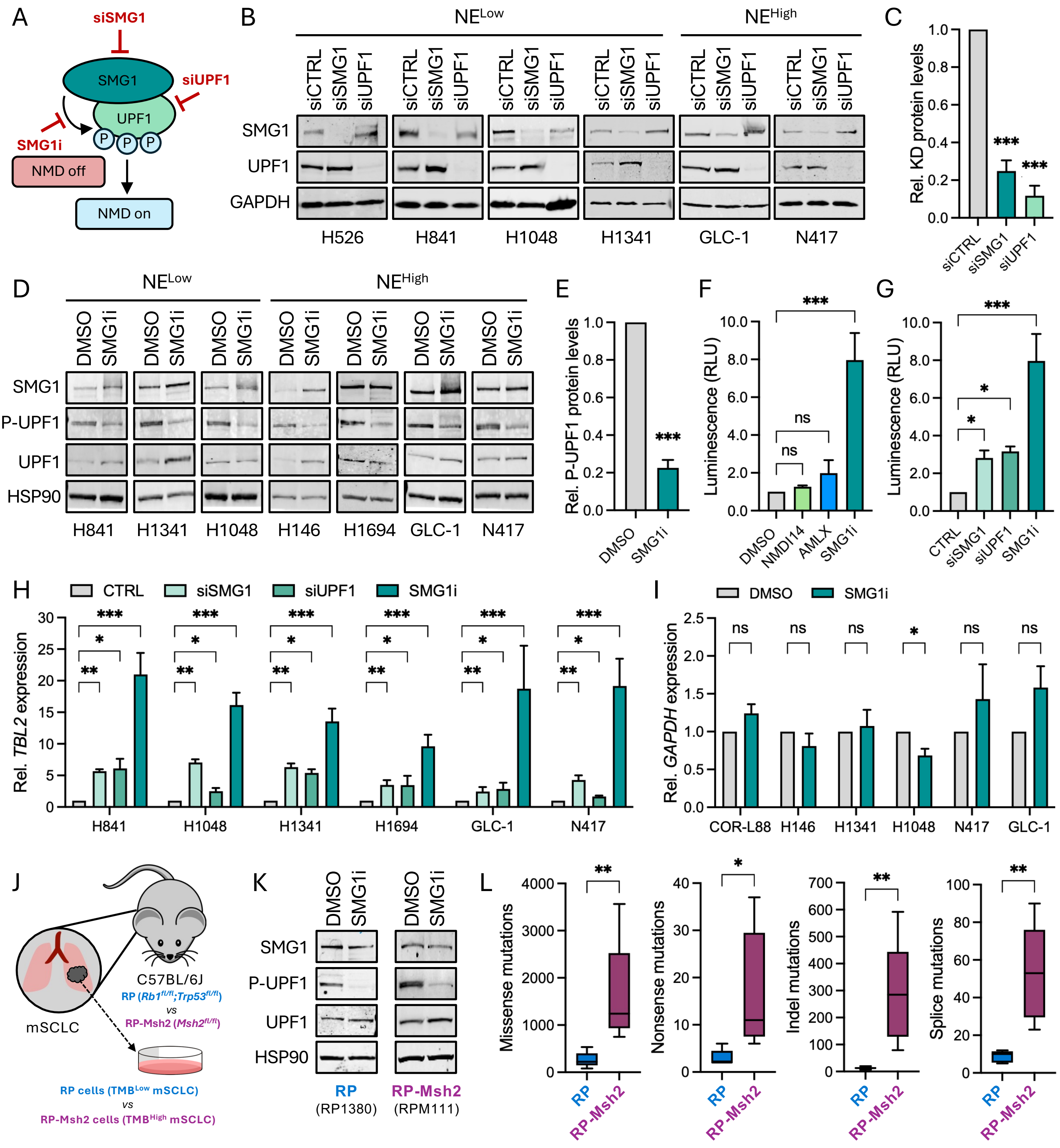

A
siSMG1
SMG1
siUPF1
UPF1
SMG1i
NMD off
NMD on
B
NELow
NEHigh
siCTRL siSMG1 siUPF1
SMG1
UPF1
GAPDH
H526 H841 H1048 H1341 GLC-1 N417
C
Rel. KD protein levels
siCTRL siSMG1 siUPF1
D
DMSO SMG1i
SMG1
P-UPF1
UPF1
HSP90
H841 H1341 H1048 H146 H1694 GLC-1 N417
E
Rel. P-UPF1 protein levels
DMSO SMG1i
F
Luminescence (RLU)
DMSO NMDI14 AMLX SMG1i
G
Luminescence (RLU)
CTRL siSMG1 siUPF1 SMG1i
H
CTRL siSMG1 siUPF1 SMG1i
Rel. TBL2 expression
H841 H1048 H1341 H1694 GLC-1 N417
I
DMSO SMG1i
Rel. GAPDH expression
COR-L88 H146 H1341 H1048 N417 GLC-1
J
C57BL/6J
RP (Rb1fl/fl;Trp53fl/fl)
vs
RP-Msh2 (Msh2fl/fl)
mSCLC
RP cells (TMBLow mSCLC)
vs
RP-Msh2 cells (TMBHigh mSCLC)
K
DMSO SMG1i
SMG1
P-UPF1
UPF1
HSP90
RP (RP1380)
RP-Msh2 (RPM111)
L
Missense mutations
Nonsense mutations
Indel mutations
Splice mutations
RP RP-Msh2

Figure S2 – related to Figure 2. Transient inhibition of the NMD pathway through siRNA mediated knockdown and chemical inhibition. A) Schematic representation of the NMD pathway and approaches for NMD inhibition by siRNA-mediated knockdown (KD) of *SMG1* (siSMG1) or *UPF1* (siUPF1), or chemical inhibition of the SMG1 kinase (SMG1i). **B)** Representative western blot for n = 4 SCLC cell lines, the SMARCA4-undifferentiated (UT) cell line H841[127] and the small cell carcinoma of the cervix cell line H1341[127] 72 hours post-transfection (hpt) with siCTRL/siSMG1/siUPF1. Neuroendocrine phenotypes (NE^{High} and NE^{Low}) are indicated. **C)** Band densitometry quantification of western blots depicted in **B** for the SMG1-KD and UPF1-KD protein levels (relative to GAPDH) in siSMG1- and siUPF1-transfected cells, respectively. **D)** Representative western blot for n = 5 SCLC cell lines, the SMARCA4-undifferentiated (UT) cell line H841[127] and the small cell carcinoma of the cervix cell line H1341[127] 24 hours post-treatment with 0.5 μM SMG1i. NE^{High} and NE^{Low} phenotypes are indicated. **E)** Band densitometry quantification of western blots depicted in **D** for the levels of P-UPF1 (relative to UPF1/HSP90). **F-G)** NMD inhibition quantified by dual luciferase reporter assays comparing SMG1i treatment (0.5 μM, 24 h) with **(F)** previously reported NMD inhibitors NMD14[69] (50 μM, 48 h) and Amlexanox[68] (AMLX, 100 μM, 48 h) (n = 3), and **(G)** with siRNA-mediated KD of SMG1 and UPF1 (n = 4). **H-I**) qPCR analysis in SCLC and H841 cancer cell lines following NMD inhibition through siSMG1/siUPF1/SMG1i treatment quantifying levels of (**H**) the *bona fide* NMD target *TBL2* (n ≥ 3) and **(I)** *GAPDH as* a non-target of NMD. **J)** Schematic representation for mSCLC cell lines derived from lung tumors arising in RP (*$Rb1^{fl/fl}$;$Trp53^{fl/fl}$*; TMB^{Low}) and RP-Msh2 mice (*$Rb1^{fl/fl}$;$Trp53^{fl/fl}$;$Msh2^{fl/fl}$;* TMB^{High}) following Adeno-CMV-Cre inhalation. **K)** Western blot showing the SMG1i-mediated inhibition of UPF1 phosphorylation in representative murine RP and RP-Msh2 cell lines. **L)** Comparison of exome mutations in RP *vs* RP-Msh2 cell lines. Bars represent mean + SEM. ***P < 0.001; **P < 0.01; *P < 0.05; ns = non-significant (One-sample t-test for C and E; One-way ANOVA for F and G, Two-way ANOVA for H; unpaired t-tests for I).

Figure S3

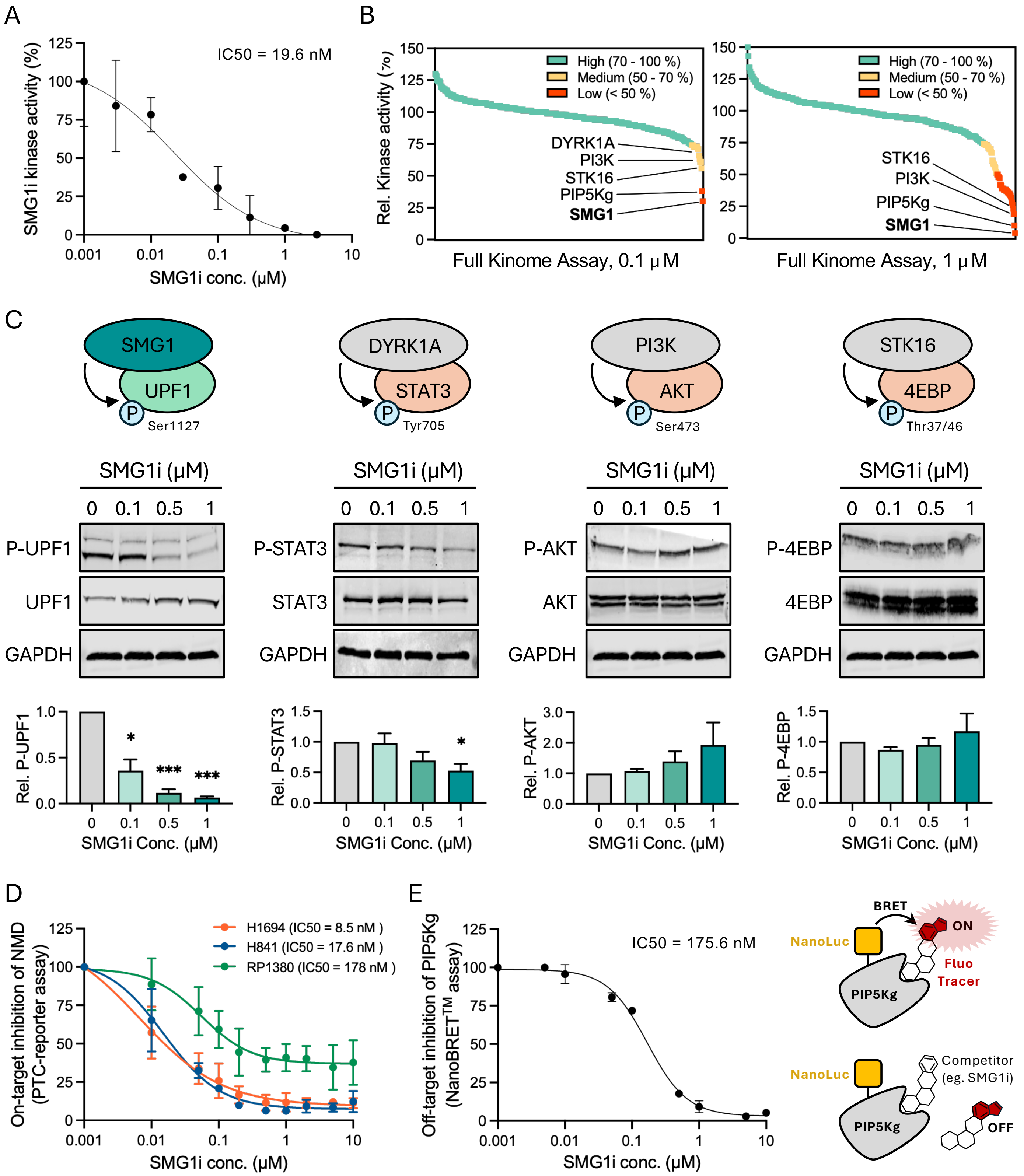

A
IC50 = 19.6 nM
SMG1i kinase activity (%)
SMG1i conc. (μM)
B
Rel. Kinase activity (%)
High (70 - 100 %)
Medium (50 - 70 %)
Low (< 50 %)
DYRK1A
PI3K
STK16
PIP5Kg
SMG1
Full Kinome Assay, 0.1 μM
Full Kinome Assay, 1 μM
C
SMG1
UPF1
Ser1127
DYRK1A
STAT3
Tyr705
PI3K
AKT
Ser473
STK16
4EBP
Thr37/46
SMG1i (μM)
0 0.1 0.5 1
P-UPF1
UPF1
GAPDH
P-STAT3
STAT3
P-AKT
AKT
P-4EBP
4EBP
Rel. P-UPF1
Rel. P-STAT3
Rel. P-AKT
Rel. P-4EBP
SMG1i Conc. (μM)
D
H1694 (IC50 = 8.5 nM)
H841 (IC50 = 17.6 nM)
RP1380 (IC50 = 178 nM)
On-target inhibition of NMD (PTC-reporter assay)
SMG1i conc. (μM)
E
IC50 = 175.6 nM
Off-target inhibition of PIP5Kg (NanoBRET™ assay)
SMG1i conc. (μM)
BRET
NanoLuc
ON
Fluo Tracer
PIP5Kg
Competitor (eg. SMG1i)
OFF

Figure S3 – related to Figure 2. The compound SMG1i-11j is a potent and highly selective SMG1 kinase inhibitor. A) Enzymatic *in vitro* phosphorylation assay for full-length recombinant SMG1 protein using p53-Ser15 as a phosphorylation substrate in the presence of increasing SMG1i concentrations. IC50 was calculated by non-linear adjustment of the kinase activity inhibitory curve. **B)** Kinome assay on 436 kinases for selected SMG1i concentrations showing the relative kinase activity (%) for each individual kinase upon NMD inhibition with 0.1 μM **(left)** and 1 μM SMG1i **(right)**, with selected kinases highlighted (see **Table S2A**). **C)** Western blot evaluation of the off-target inhibition of selected kinases in COR-L88 cells (NE^{High} SCLC) treated with increasing concentrations of SMG1i, assessing for the respective kinase activity by probing for the phosphorylated downstream substrate as indicated in the scheme (**upper panel**). Quantifications based on band densitometry are provided (**lower panels**). Bars represent mean + SEM (n = 3); ***P<0.001; *P<0.05 (Unpaired t-tests *vs* untreated sample). **E)** On-target inhibition of NMD by SMG1i assessed by cell-based luciferase reporter assays in human SCLC (H1694), human SMARCA4-UT (H841) and murine SCLC (RP1380) cell lines transfected with the NMD-sensitive PTC-reporter and treated with increasing SMG1i concentrations. IC50 values were calculated by non-linear adjustment of NMD inhibitory curves (n = 3). **F)** Off-target inhibition of PIP5Kg by SMG1i assessed by NanoBRET assays, with IC50 value calculated by non-linear adjustment of PIP5Kg inhibitory curve (**left**), accompanied by a schematic representation of the NanoBRET assay principle (**right**).

Figure S4

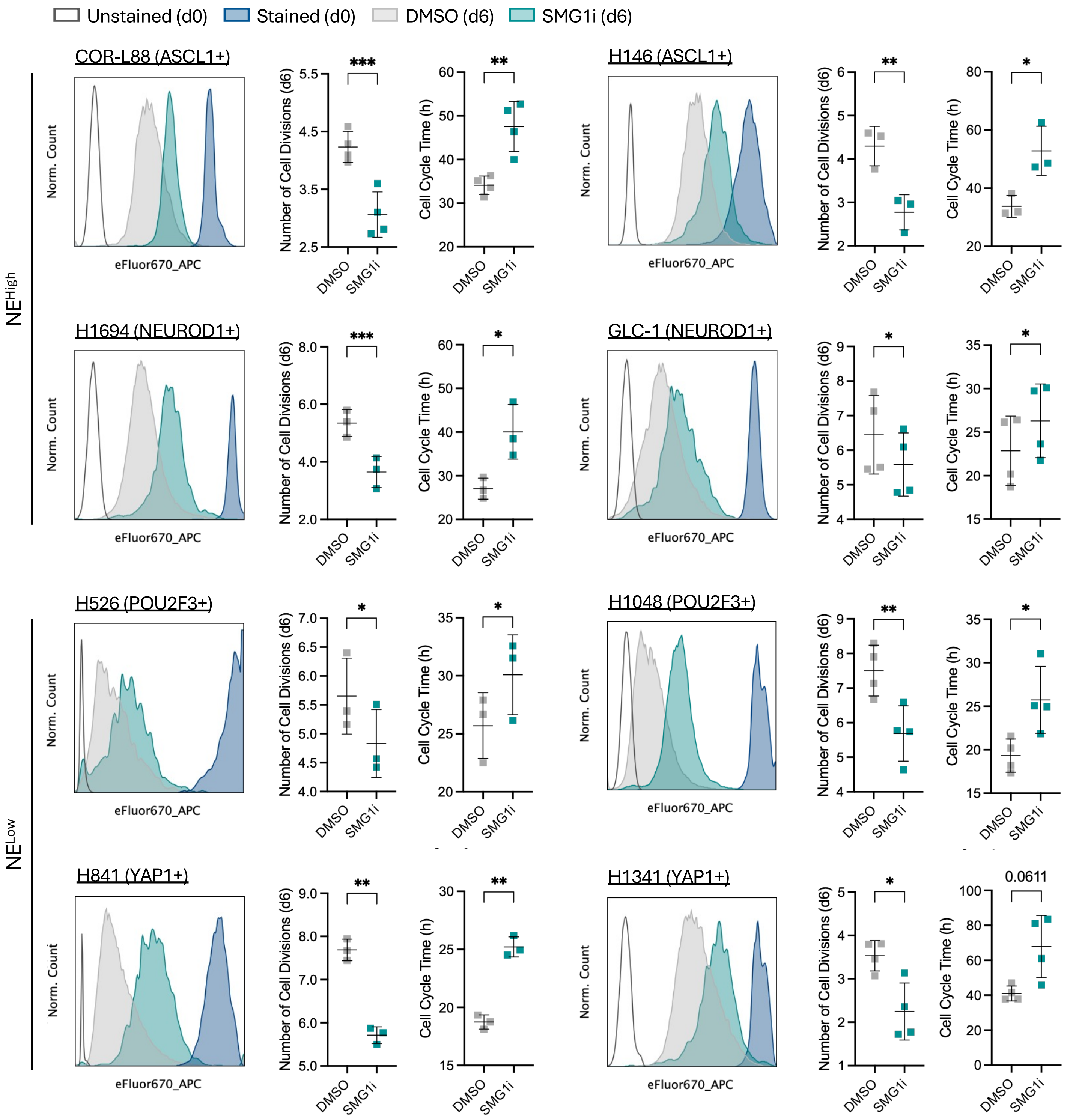
Unstained (d0)
Stained (d0)
DMSO (d6)
SMG1i (d6)
NE^High
NE^Low
COR-L88 (ASCL1+)
H146 (ASCL1+)
H1694 (NEUROD1+)
GLC-1 (NEUROD1+)
H526 (POU2F3+)
H1048 (POU2F3+)
H841 (YAP1+)
H1341 (YAP1+)
Norm. Count
eFluor670_APC
Number of Cell Divisions (d6)
Cell Cycle Time (h)
DMSO
SMG1i
0.0611

Figure S4 – related to Figure 2. Inhibition of NMD restricts cell proliferation in SCLC. Proliferation assays for n = 8 human SCLC cell lines (two of each SCLC subtype: COR-L88 and H146 as ASCL1+; N417 and GLC-1 as NEUROD1+; H526 and H1048 as POU2F3+; H841 and H1341 as YAP1+, with H841 recently reclassified as a SMARCA4-UT cell line and H1341 as a small cell carcinoma of the cervix cell line[127]. Neuroendocrine phenotype is indicated as NE^{High} or NE^{Low}. For each cell line, a representative flow cytometry eFluor670 histogram for DMSO *vs* SMG1i-treated cells 6 days post-staining/treatment is shown (**left**), along with a quantification of the number of cell divisions after 6 days based on the loss of eFluor670 fluorescence intensity over time, calculated as Log2 $[(MFI_{day0} – MFI_{unstained})/(MFI_{dayX} – MFI_{unstained})]$ (**middle**), and the estimated average duration of cell cycle time in hours (**right**).

Figure S5

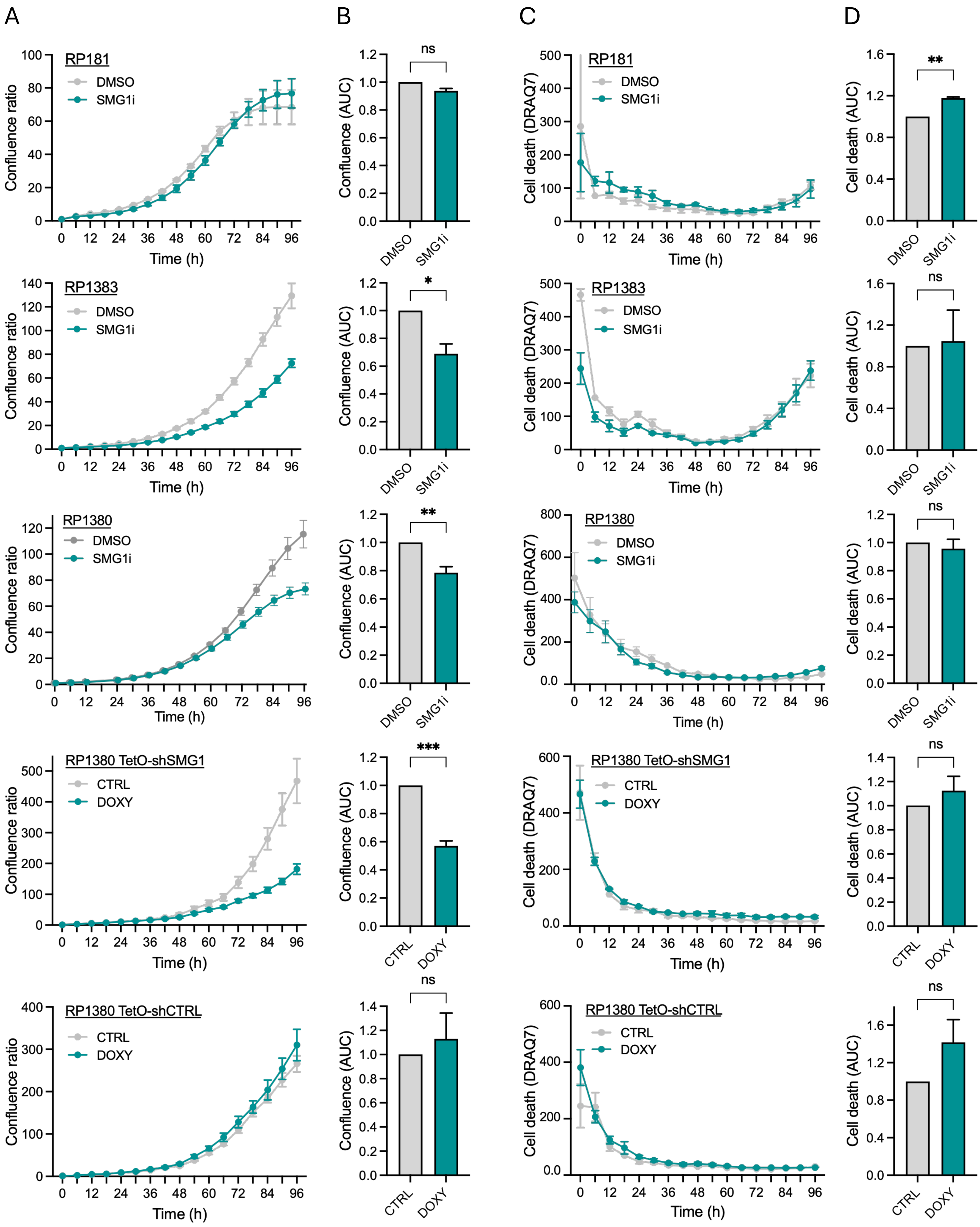
A
B
C
D
RP181
DMSO
SMG1i
Confluence ratio
Time (h)
Confluence (AUC)
ns
Cell death (DRAQ7)
**
Cell death (AUC)
RP1383
*
RP1380
RP1380 TetO-shSMG1
CTRL
DOXY
***
RP1380 TetO-shCTRL

Figure S5 – related to Figure 2. Inhibition of NMD does not induce cell death in murine TMB^{low} SCLC cell lines. A-D) Representative curves for cell growth (confluence ratio, **A**) and cell death (DRAQ7 staining, **C**) determined by live-cell imaging accompanied by area under curve (AUC) quantifications (**B** and **D**, respectively) for individual experiments (n = 3) in several murine TMB^{low} SCLC cell lines. All graphs represent mean + SEM. ***P < 0.001; **P < 0.01; *P < 0.05; ns = non-significant (Unpaired t-tests on AUC quantification). The three upper panels refer to chemical NMD inhibition treatment with DMSO *vs* SMG1i, and the two lower panels refer to DMSO vs doxycycline (DOXY) treatment of RP1380 TetO-shSMG1 cells for genetic NMD inhibition via SMG1-KD, and of RP1380 TetO-shCTRL cells as a control to account for doxycycline-associated effects. Proof of doxycycline-inducible SMG1-KD in representative cellular models is shown in **Figs. S8C-F**.

Figure S6

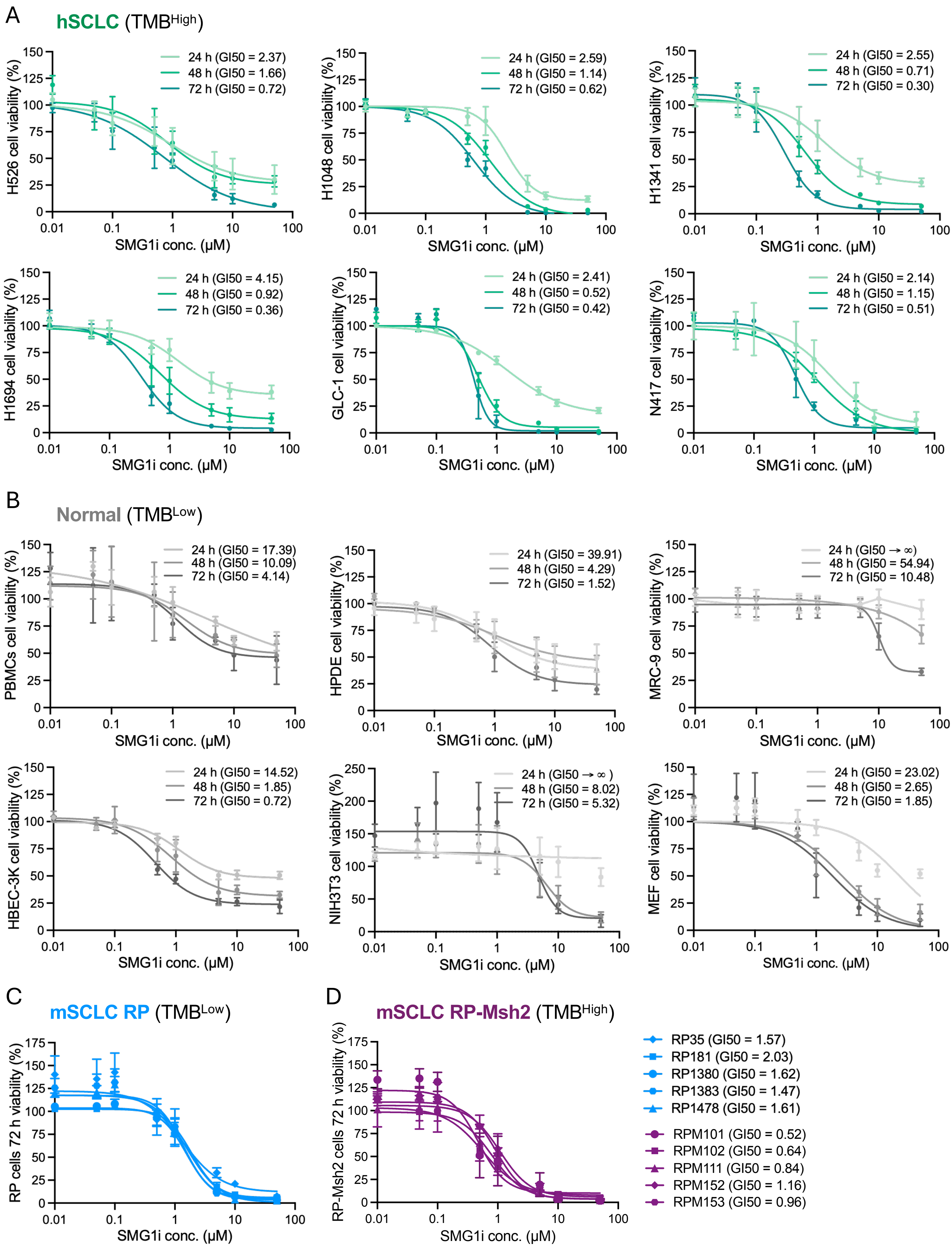
A
hSCLC (TMBHigh)
H526 cell viability (%)
24 h (GI50 = 2.37)
48 h (GI50 = 1.66)
72 h (GI50 = 0.72)
H1048 cell viability (%)
24 h (GI50 = 2.59)
48 h (GI50 = 1.14)
72 h (GI50 = 0.62)
H1341 cell viability (%)
24 h (GI50 = 2.55)
48 h (GI50 = 0.71)
72 h (GI50 = 0.30)
H1694 cell viability (%)
24 h (GI50 = 4.15)
48 h (GI50 = 0.92)
72 h (GI50 = 0.36)
GLC-1 cell viability (%)
24 h (GI50 = 2.41)
48 h (GI50 = 0.52)
72 h (GI50 = 0.42)
N417 cell viability (%)
24 h (GI50 = 2.14)
48 h (GI50 = 1.15)
72 h (GI50 = 0.51)
SMG1i conc. (μM)
B
Normal (TMBLow)
PBMCs cell viability (%)
24 h (GI50 = 17.39)
48 h (GI50 = 10.09)
72 h (GI50 = 4.14)
HPDE cell viability (%)
24 h (GI50 = 39.91)
48 h (GI50 = 4.29)
72 h (GI50 = 1.52)
MRC-9 cell viability (%)
24 h (GI50 → ∞)
48 h (GI50 = 54.94)
72 h (GI50 = 10.48)
HBEC-3K cell viability (%)
24 h (GI50 = 14.52)
48 h (GI50 = 1.85)
72 h (GI50 = 0.72)
NIH3T3 cell viability (%)
24 h (GI50 → ∞)
48 h (GI50 = 8.02)
72 h (GI50 = 5.32)
MEF cell viability (%)
24 h (GI50 = 23.02)
48 h (GI50 = 2.65)
72 h (GI50 = 1.85)
SMG1i conc. (μM)
C
mSCLC RP (TMBLow)
RP cells 72 h viability (%)
D
mSCLC RP-Msh2 (TMBHigh)
RP-Msh2 cells 72 h viability (%)
SMG1i conc. (μM)
RP35 (GI50 = 1.57)
RP181 (GI50 = 2.03)
RP1380 (GI50 = 1.62)
RP1383 (GI50 = 1.47)
RP1478 (GI50 = 1.61)
RPM101 (GI50 = 0.52)
RPM102 (GI50 = 0.64)
RPM111 (GI50 = 0.84)
RPM152 (GI50 = 1.16)
RPM153 (GI50 = 0.96)

Figure S6 – related to Figure 2. Cell-intrinsic vulnerability to NMD inhibition correlates with TMB, suggesting a therapeutic window. A-C) CTG-viability assays following treatment at the indicated times with increasing concentrations of SMG1i for (**A**) the indicated human TMB^{High} SCLC cell lines, (**B**) TMB^{Low} non-neoplastic (normal/healthy) cells, including PMBCs = peripheral blood mononuclear cells (primary); HPDE = human pancreatic duct epithelial cell line; MRC-9 = lung human fibroblast cell line; HBEC-3K = human bronchial epithelial cells; MEFs = mouse embryonic fibroblasts; NIH3T3= mouse embryonic fibroblasts; and (**C**) TMB^{Low} murine SCLC RP (*Rb1-/-; Trp53-/-*) primary cells and (**D**) TMB^{High} murine SCLC RP-Msh2 (*Rb1-/-; Trp53-/-; Msh2-/-*) primary cells. The growth inhibitory concentration GI50 was estimated from non-linear adjustments of viability curves. Graphs represent mean + SEM (n ≥ 3).

Figure S7

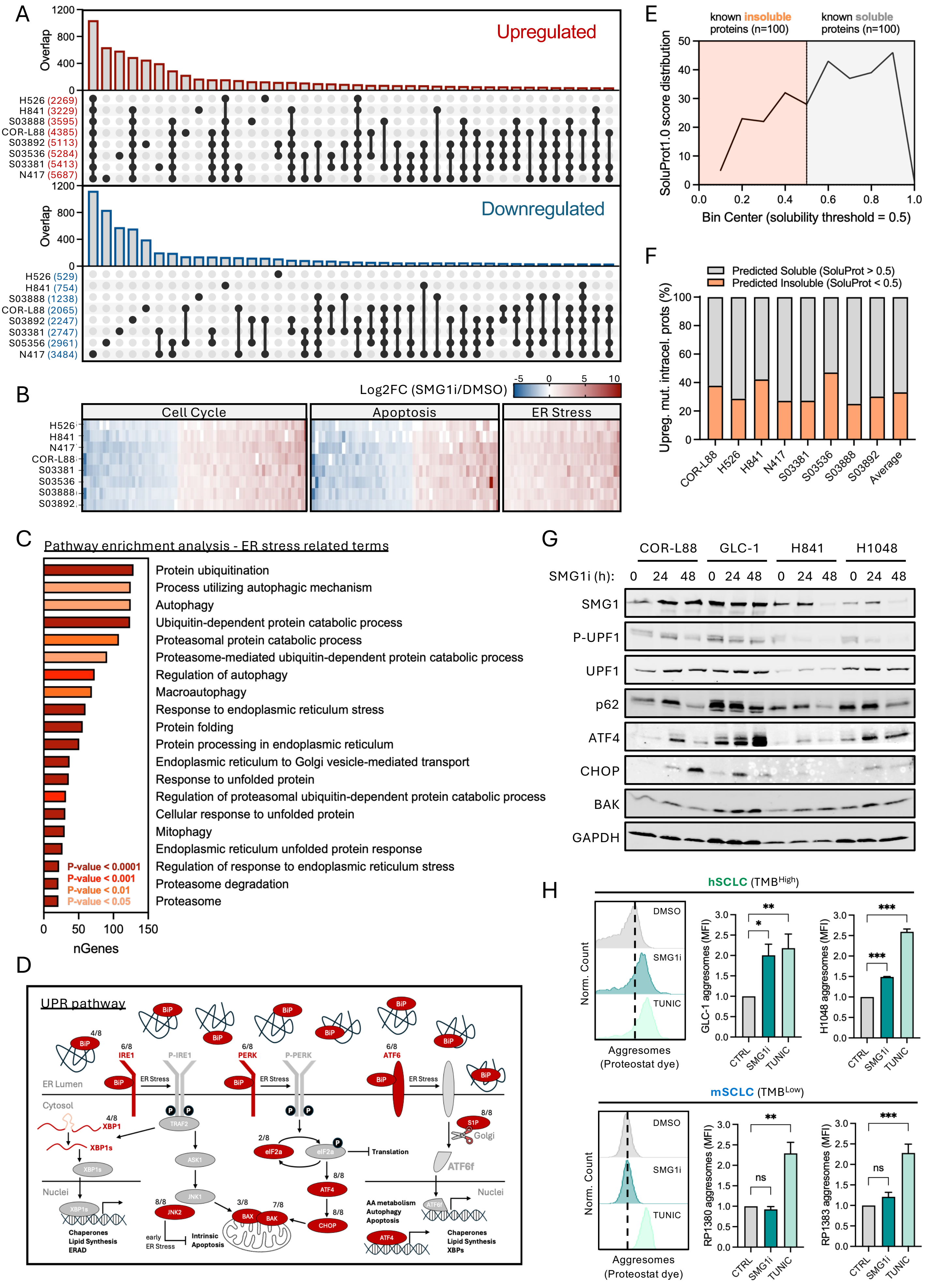
A
Upregulated
Overlap
H526 (2269)
H841 (3229)
S03888 (3595)
COR-L88 (4385)
S03892 (5113)
S03536 (5284)
S03381 (5413)
N417 (5687)
Downregulated
H526 (529)
H841 (754)
S03888 (1238)
COR-L88 (2065)
S03892 (2247)
S03381 (2747)
S05356 (2961)
N417 (3484)
B
Log2FC (SMG1i/DMSO)
Cell Cycle
Apoptosis
ER Stress
H526
H841
N417
COR-L88
S03381
S03536
S03888
S03892
C
Pathway enrichment analysis - ER stress related terms
Protein ubiquitination
Process utilizing autophagic mechanism
Autophagy
Ubiquitin-dependent protein catabolic process
Proteasomal protein catabolic process
Proteasome-mediated ubiquitin-dependent protein catabolic process
Regulation of autophagy
Macroautophagy
Response to endoplasmic reticulum stress
Protein folding
Protein processing in endoplasmic reticulum
Endoplasmic reticulum to Golgi vesicle-mediated transport
Response to unfolded protein
Regulation of proteasomal ubiquitin-dependent protein catabolic process
Cellular response to unfolded protein
Mitophagy
Endoplasmic reticulum unfolded protein response
Regulation of response to endoplasmic reticulum stress
Proteasome degradation
Proteasome
P-value < 0.0001
P-value < 0.001
P-value < 0.01
P-value < 0.05
nGenes
D
UPR pathway
ER Lumen
Cytosol
Nuclei
IRE1
P-IRE1
PERK
P-PERK
ATF6
BiP
ER Stress
TRAF2
ASK1
JNK1
JNK2
XBP1
XBP1s
eIF2a
ATF4
CHOP
BAX
BAK
Translation
S1P
Golgi
ATF6f
Chaperones
Lipid Synthesis
ERAD
early ER Stress
Intrinsic Apoptosis
AA metabolism
Autophagy
Apoptosis
XBPs
E
known insoluble proteins (n=100)
known soluble proteins (n=100)
SoluProt1.0 score distribution
Bin Center (solubility threshold = 0.5)
F
Predicted Soluble (SoluProt > 0.5)
Predicted Insoluble (SoluProt < 0.5)
Upreg. mut. intracel. prots (%)
COR-L88
H526
H841
N417
S03381
S03536
S03888
S03892
Average
G
COR-L88
GLC-1
H841
H1048
SMG1i (h):
0 24 48 0 24 48 0 24 48 0 24 48
SMG1
P-UPF1
UPF1
p62
ATF4
CHOP
BAK
GAPDH
H
hSCLC (TMBHigh)
DMSO
SMG1i
TUNIC
Norm. Count
Aggresomes
(Proteostat dye)
GLC-1 aggresomes (MFI)
H1048 aggresomes (MFI)
CTRL
SMG1i
TUNIC
mSCLC (TMBLow)
RP1380 aggresomes (MFI)
RP1383 aggresomes (MFI)
ns

Figure S7 – related to Figure 3. NMD inhibition triggers ER stress and upregulates the UPR pathway. A-C) Transcriptome sequencing data for indicated SCLC cell lines (n = 4) and patient-derived SCLC tumor cells (n = 4) treated with DMSO *vs* SMG1i for 24 h (**Table S3**). UpSet plots for differential transcript expression (DTE) overlaps (**Tables S4A-B**) with the number of differentially up/downregulated transcripts (Log2FC>|1|, Padj<0.05, n=3) indicated by colored numbers within brackets **(A)**. Heatmap for differentially expressed transcripts involved in cell cycle, apoptosis and ER stress-related processes identified through pathway enrichment analysis (**Tables S4C-D** and **Methods**) **(B)**. Pathway enrichment analysis based on transcriptome sequencing data of n = 8 SCLC samples treated with DMSO *vs* SMG1i (See **Tables S3** and **S4**). Transcripts commonly upregulated (Log2FC > 1 and Padj < 0.05) upon SMG1i treatment in at least 6 out of 8 SCLC samples revealed an enrichment of multiple ER stress-related terms (**C**). **D)** Schematic representation of the ER stress response pathway also known as the unfolded protein response (UPR) pathway highlighting in red those proteins for which transcripts were found significantly upregulated in at least n=2 SCLC samples. The number of samples for which such an upregulation was observed is indicated as a fraction (e.g. 2/8) next to the respective protein. **E)** Distribution of protein solubility scores determined with the Protein solubility predictor SoluProt1.0 for n=100 known soluble and n=100 known insoluble proteins (based on experimental solubility upon expression in *E.Coli*) extracted from the SoluProt1.0 validation set[131] with a threshold at Solubility score = 0.5. **F)** Protein solubility prediction (SoluProt1.0) for mutated intracellular proteins (see **Methods**) encoded by transcripts significantly upregulated following NMD inhibition. Proportions of predicted soluble and predicted insoluble mutant proteins are shown for each sample, and integrate upregulated PTC (frameshift and nonsense) and non-PTC (missense/in-frame) mutations. We note that main **Fig. 3B** instead presents the prediction for all upregulated PTC-mutant proteins integrated across all samples. **G)** Representative western blot showing levels of selected proteins of the UPR pathway (namely the apoptosis effector axis ATF4-CHOP-BAK1 and the autophagy substrate p62) upon SMG1i treatment in SCLC cell lines (including one cell line per SCLC subtype: COR-L88 as ASCL1+, GLC-1 as NEUROD1+, H1048 as POU2F3+ and H841 as YAP1+ which has been recently reclassified as SMARCA4-UT cell line[127]). **H)** Aggregated protein foci or *aggresomes* measured by flow cytometry (Proteostat staining, see **Methods**) 72 h post-treatment with SMG1i or Tunicamycin (TUNIC) in hSCLC (TMB^{High}) and mSCLC RP (TMB^{Low}) cell lines, accompanied by representative Proteostat histograms (n≥3) (see also **Fig. 3E**).

Figure S8

A

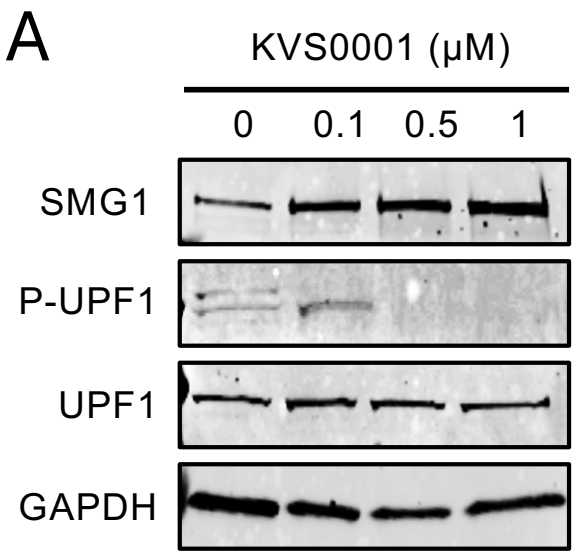

B

Matrix	KVS0001 conc. (mpk)	No_points_ lambda_z	$t_{1/2}$ (h)	T_{max} (h)	C_{max} (ng/g)	AUC_{0-t} (h*ng/g)	$AUC_{0-\infty obs}$ (h*ng/g)	AUC_%Extra p_obs(%)	$AUC_{0-t\_D\_obs}$ (h*kg*ng/g/mg)	MRT_{0-t} (h)
Plasma	25,0	7,0	7,6	0,5	966,7	4066,8	4078,1	0,3	162,7	5,3
	50,0	7,0	8,7	0,5	1246,3	6744,1	6796,1	0,8	134,9	7,9
Tumor	25,0	3,0	3,8	2,0	205,8	1260,9	1278,4	1,4	50,4	5,0
	50,0	3,0	6,0	2,0	253,9	2281,9	2456,2	7,1	45,6	6,5

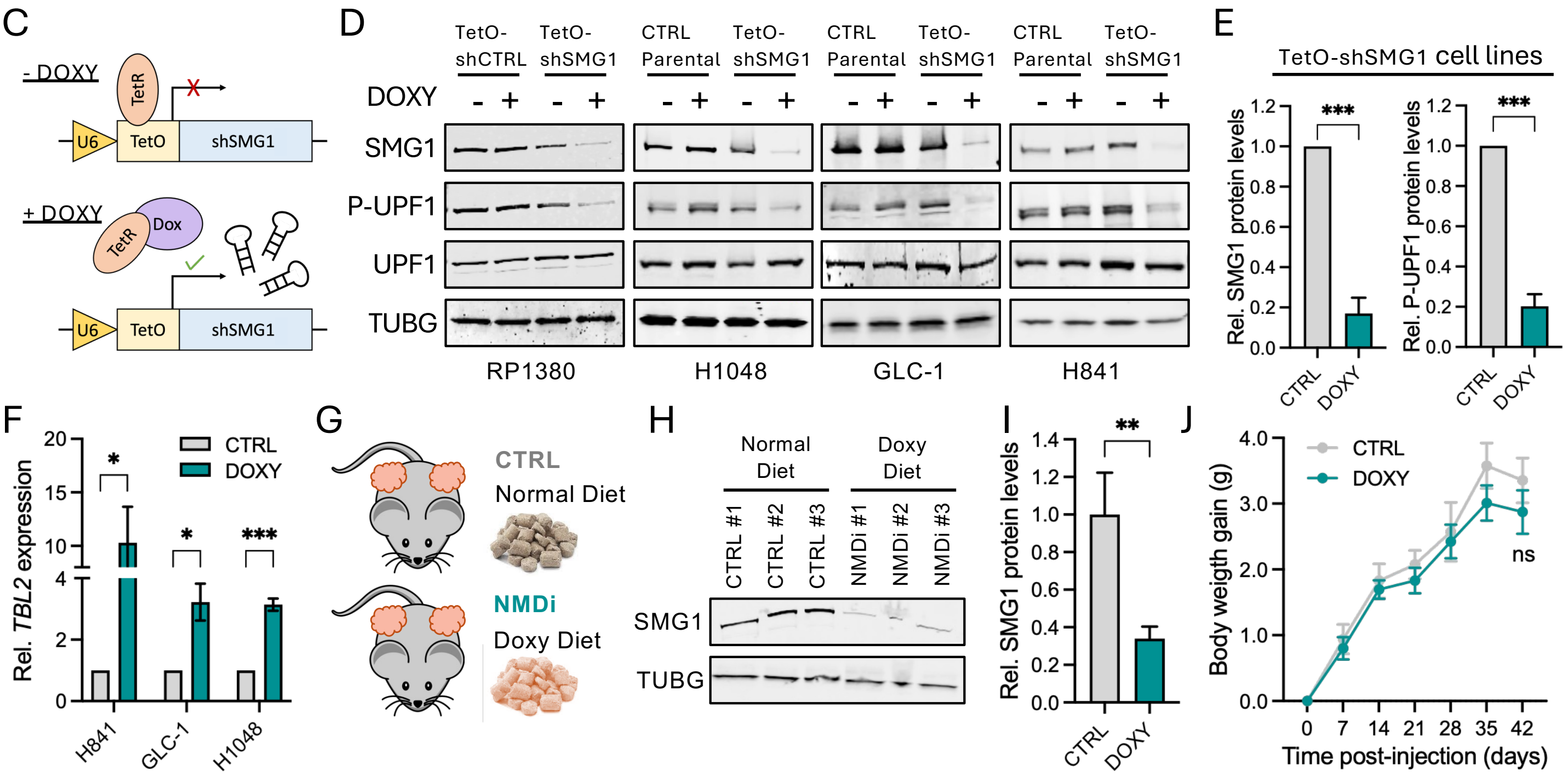

Figure S8 – related to Figure 4. Approaches for *in vivo* inhibition of NMD: pharmacological systemic inhibition via KVS0001 treatment and genetic tumor-targeted inhibition via doxycycline-inducible SMG1-KD. A) Western blot on the human COR-L88 SCLC cell line (NE^{High}, ASCL1+) treated *in vitro* with increasing concentrations of the SMG1i-derivate compound KVS0001 for 24 h. **B)** Pharmacokinetic parameters for KVS0001 compound detected by mass spectrometry in plasma and tumors of NSG mice after a single intraperitoneal injection with 25 or 50 milligram per kg (mpk) at timepoints post-injection indicated in **Fig. 4C-D. C)** Schematic representation for the doxycycline-inducible SMG1-KD operating within TetO-shSMG1 models, which include the murine SCLC cell lines RP1380 and RPM111, the human SMARCA-UT cell line H841, the human NE^{High} SCLC cell line GLC-1 and the human NE^{Low} SCLC cell line H1048. **D)** Western blots for representative TetO-shSMG1 cell lines probing for downregulation of SMG1 and consequently decreased P-UPF1 levels upon adding 2 ug/ml doxycycline to the cell culture for 72h. For human TetO-shSMG1 cell lines, the corresponding parental cell lines served as control to account for the effects of doxycycline treatment. For the murine SCLC cell line RP1380, a TetO-shCTRL cell line was generated to exclude immunogenic effects of the TetO system in subsequent experiments shown in **Fig. 6**. **E)** Band densitometry quantification for SMG1 (relative to TUBG) and P-UPF1 (relative to UPF1/TUBG) protein levels in TetO-shSMG1 cell lines referring to the data in panel **D**. **F)** qPCR quantification of the *bona fide* NMD target *TBL2* in human TetO-shSMG1 cell lines after 72 h of *in vitro* doxycycline treatment. **G)** Scheme for *in vivo* induction of SMG1-KD through the administration of doxycycline-containing diet to either NSG (**Fig. 4**) or C57BL/6J (**Fig. 6**) mice subcutaneously implanted with TetO-shSMG1 cells. **H)** Representative blot for SMG1 protein levels from allograft RP1380 TetO-shSMG1 subcutaneous tumors derived from C57BL/6J mice fed with normal or doxycycline diet. **I)** Band densitometry quantification for SMG1 protein levels (relative to TUBG) determined in western blots shown in **H** and additional independent blots ($n \geq 14$ tumors). **I)** Mouse weight changes over time in pooled NSG and C57BL/6J mice receiving normal or doxycycline diet ($n = 16$).

Figure S9

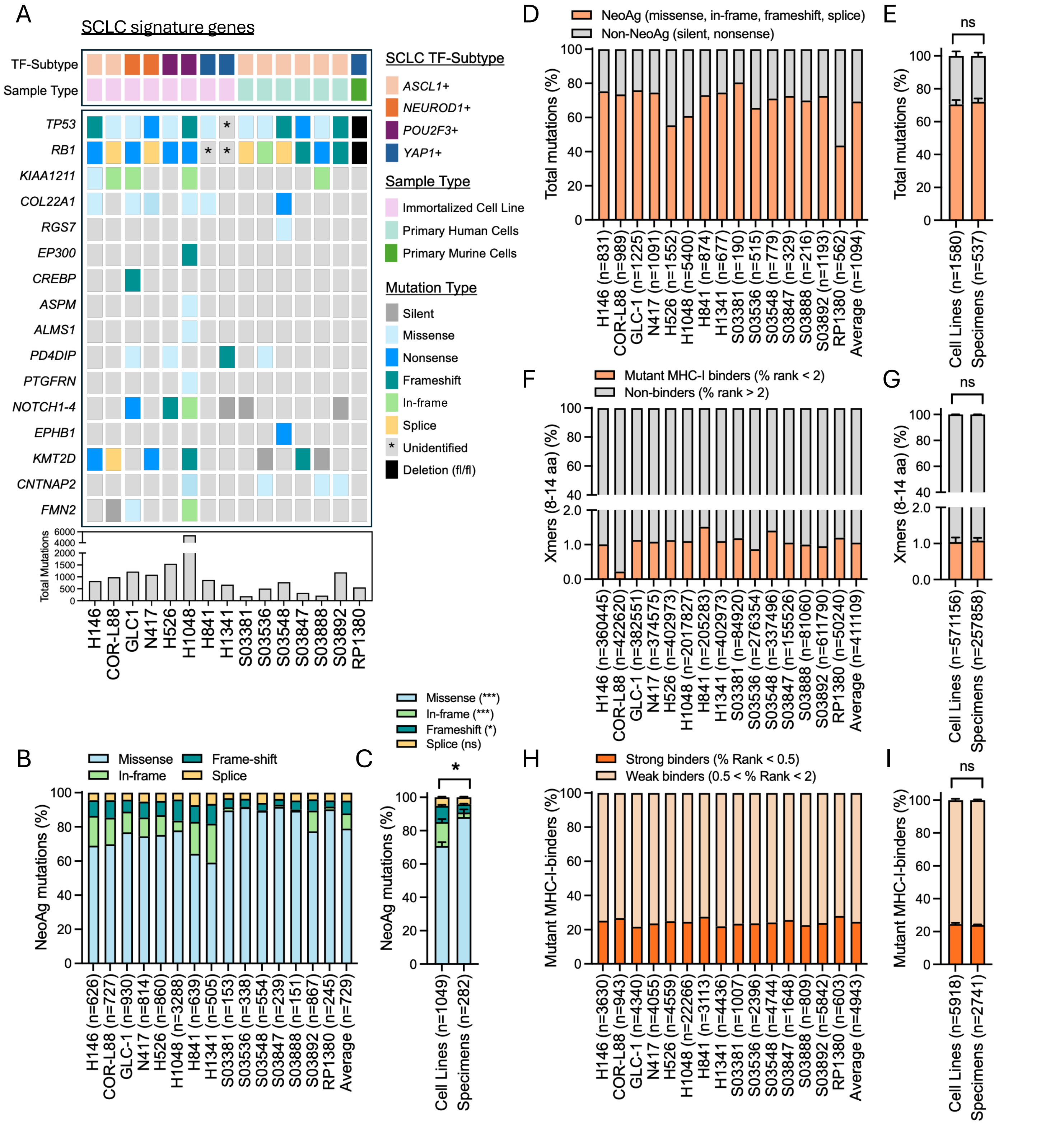
A
SCLC signature genes
TF-Subtype
Sample Type
TP53
RB1
KIAA1211
COL22A1
RGS7
EP300
CREBP
ASPM
ALMS1
PD4DIP
PTGFRN
NOTCH1-4
EPHB1
KMT2D
CNTNAP2
FMN2
Total Mutations
H146
COR-L88
GLC1
N417
H526
H1048
H841
H1341
S03381
S03536
S03548
S03847
S03888
S03892
RP1380
SCLC TF-Subtype
ASCL1+
NEUROD1+
POU2F3+
YAP1+
Sample Type
Immortalized Cell Line
Primary Human Cells
Primary Murine Cells
Mutation Type
Silent
Missense
Nonsense
Frameshift
In-frame
Splice
Unidentified
Deletion (fl/fl)
B
Missense
In-frame
Frame-shift
Splice
NeoAg mutations (%)
H146 (n=626)
COR-L88 (n=727)
GLC-1 (n=930)
N417 (n=814)
H526 (n=860)
H1048 (n=3288)
H841 (n=639)
H1341 (n=505)
S03381 (n=153)
S03536 (n=338)
S03548 (n=554)
S03847 (n=239)
S03888 (n=151)
S03892 (n=867)
RP1380 (n=245)
Average (n=729)
C
Missense (***)
In-frame (***)
Frameshift (*)
Splice (ns)
NeoAg mutations (%)
Cell Lines (n=1049)
Specimens (n=282)
D
NeoAg (missense, in-frame, frameshift, splice)
Non-NeoAg (silent, nonsense)
Total mutations (%)
H146 (n=831)
COR-L88 (n=989)
GLC-1 (n=1225)
N417 (n=1091)
H526 (n=1552)
H1048 (n=5400)
H841 (n=874)
H1341 (n=677)
S03381 (n=190)
S03536 (n=515)
S03548 (n=779)
S03847 (n=329)
S03888 (n=216)
S03892 (n=1193)
RP1380 (n=562)
Average (n=1094)
E
ns
Total mutations (%)
Cell Lines (n=1580)
Specimens (n=537)
F
Mutant MHC-I binders (% rank < 2)
Non-binders (% rank > 2)
Xmers (8-14 aa) (%)
H146 (n=360445)
COR-L88 (n=422620)
GLC-1 (n=382551)
N417 (n=374575)
H526 (n=402973)
H1048 (n=2017827)
H841 (n=205283)
H1341 (n=402973)
S03381 (n=84920)
S03536 (n=276354)
S03548 (n=337496)
S03847 (n=155526)
S03888 (n=81060)
S03892 (n=611790)
RP1380 (n=50240)
Average (n=411109)
G
ns
Xmers (8-14 aa) (%)
Cell Lines (n=571156)
Specimens (n=257858)
H
Strong binders (% Rank < 0.5)
Weak binders (0.5 < % Rank < 2)
Mutant MHC-I-binders (%)
H146 (n=3630)
COR-L88 (n=943)
GLC-1 (n=4340)
N417 (n=4055)
H526 (n=4559)
H1048 (n=22266)
H841 (n=3113)
H1341 (n=4436)
S03381 (n=1007)
S03536 (n=2396)
S03548 (n=4744)
S03847 (n=1648)
S03888 (n=809)
S03892 (n=5842)
RP1380 (n=603)
Average (n=4943)
I
ns
Mutant MHC-I-binders (%)
Cell Lines (n=5918)
Specimens (n=2741)

Figure S9 – related to Figure 5. Whole exome sequencing followed by MHC-I-binding modeling predicts thousands of neoantigens in SCLC samples. A) SCLC-relevant genome alterations[3,5] determined for the indicated SCLC cell lines and patient-derived tumor specimens by whole exome sequencing processed with our in-house pipeline. **B-C)** Proportion of potentially immunogenic neoantigen (NeoAg) mutations (i. e. missense, in-frame, frameshift and splice) for the indicated SCLC samples (**B**) and comparing cell line *vs* patient data (**C**). Nonstop mutations are included in the frameshift mutation count, and intron-exon mutations are included in the splice mutation count. **D-E)** Proportion of NeoAg and non-NeoAg mutations (silent and nonsense) for the indicated SCLC samples (**D**) and comparing cell line *vs* patient data (**E**). **F-G)** Proportion of MHC-I binders and non-binders predicted within all tested overlapping Xmers (8mers-14mers) for the indicated SCLC samples (**F**) and comparing cell line *vs* patient data (**G**). **H-I)** Proportion of strong and weak MHC-I-binders for the indicated SCLC samples (**H**) and comparing cell line *vs* patient data (**I**). Every graph indicates proportion (%), with absolute numbers (n = abs) indicated for each sample. Average values (n = abs_mean) are also indicated for all samples, for all cell lines and for all patient specimens. Error bars represent the SEM. ***P-value < 0.005; *P-value < 0.05; ns = non-significant (Unpaired t-tests:). See also **Table S5A-D.**

Figure S10

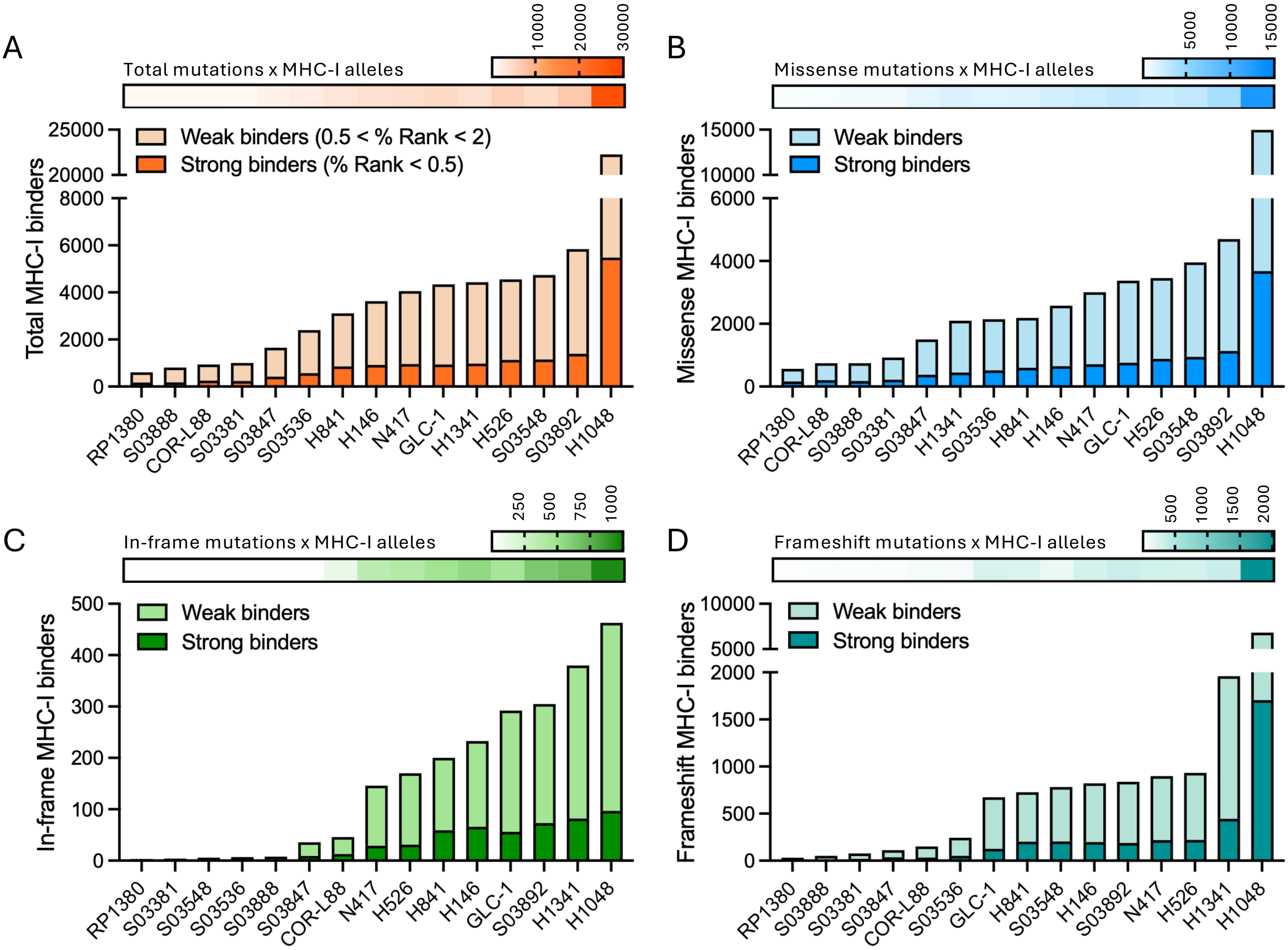

E

Sample	A1	A2	B1	B2	C1	C2
H841	HLA-A*03:01	HLA-A*66:01	HLA-B*07:02	HLA-B*15:17	HLA-C*07:01	HLA-C*07:02
H146	HLA-A*01:01	HLA-A*03:01	HLA-B*14:02	HLA-B*14:02	HLA-C*08:02	HLA-C:16:01
H1341	HLA-A*25:01	HLA-A*31:01	HLA-B*18:01	HLA-B*44:02	HLA-C*05:01	HLA-C*12:03
N417	HLA-A*23:01	HLA-A*26:01	HLA-B:08:01	HLA-B:08:01	HLA-C:07:01	HLA-C:07:01
GLC-1	HLA-A*03:01	HLA-A*68:01	HLA-B*35:01	HLA-B*35:08	HLA-C*04:01	HLA-C*04:01
H526	HLA-A*03:01	HLA-A*24:02	HLA-B*07:02	HLA-B*07:02	HLA-C*07:02	HLA-C*07:02
H1048	HLA-A*23:01	HLA-A*68:02	HLA-B*53:01	HLA-C*04:01	HLA-C*06:02	HLA-C*06:02
COR-L88	HLA-A*01:01	HLA-A*01:01	HLA-B*37:04	HLA-B*58:05	HLA-C*07:06	HLA-C*07:06
S03381	HLA-A*01:01	HLA-A*11:01	HLA-B*08:01	HLA-B*35:02	HLA-C*04:01	HLA-C*07:01
S03536	HLA-A*01:01	HLA-A*68:01	HLA-B*08:01	HLA-B*51:01	HLA-C*07:01	HLA-C*51:02
S03548	HLA-A*24:02	HLA-A*68:01	HLA-B*27:05	HLA-B*58:01	HLA-C*03:02	HLA-C*07:04
S03847	HLA-A*30:01	HLA-A*32:01	HLA-B*13:02	HLA-B*44:02	HLA-C*05:01	HLA-C*06:02
S03888	HLA-A*24:02	HLA-A*33:01	HLA-B*14:02	HLA-B*57:01	HLA-C*06:02	HLA-C*08:02
S03892	HLA-A*03:01	HLA-A*23:01	HLA-B*49:01	HLA-B*50:01	HLA-C*06:02	HLA-C*07:01
RP1380	H2-Db	H2-Kb				

F

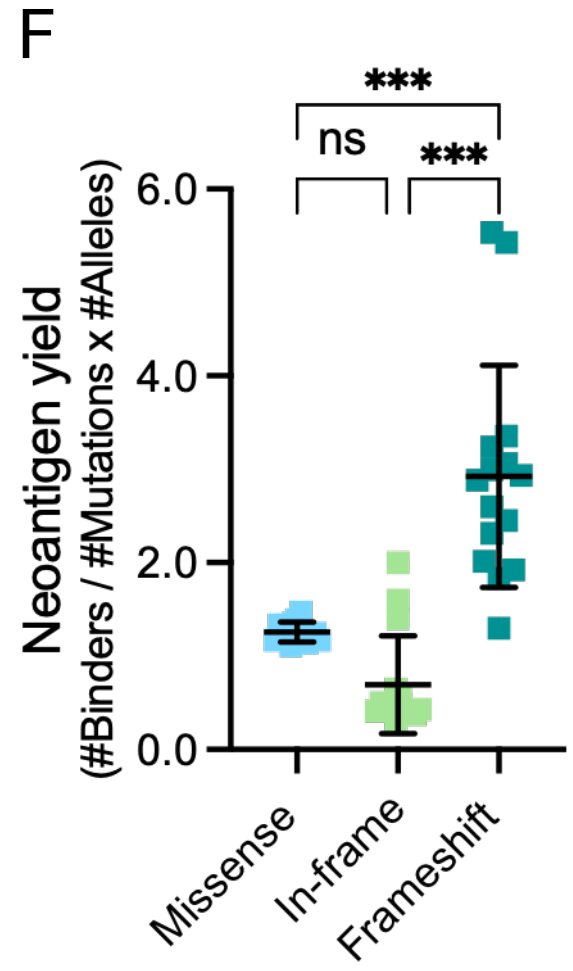

Figure S10 – related to Figure 5. Mutation type-specific neoantigen prediction reveals a higher neoantigen yield of frameshift mutations. A-D) Number of predicted MHC-I binders identified for the indicated SCLC samples for all missense, in-frame and frameshift mutations together (**A**), or independently for missense (**B**), in-frame (**C**) and frameshift mutations (**D**). The number of MHC-I-binders identified in each case highly depends on the number of input mutations and the number of input MHC-I alleles included for the prediction (shown in **E**), as shown by the upper heatmap panels in A-D. **E)** *MHC-I* locus genotyping for all samples included in the MHC-I binding prediction analysis. Alleles marked in red were not available for selection in the IEDB online *MHC-I binding tool* and therefore binders for those alleles could not be predicted. **F)** Neoantigen yield of different mutation types, which considers the number of predicted neoantigens (MHC-I-binders) per mutation and per MHC-I allele included in the prediction. Error bars represent the SD. ***P-value < 0.005; **P-value < 0.01; *P-value < 0.05 (One-way ANOVA). See also **Fig. 5A-B** and **Table S5A-D.**

Figure S11

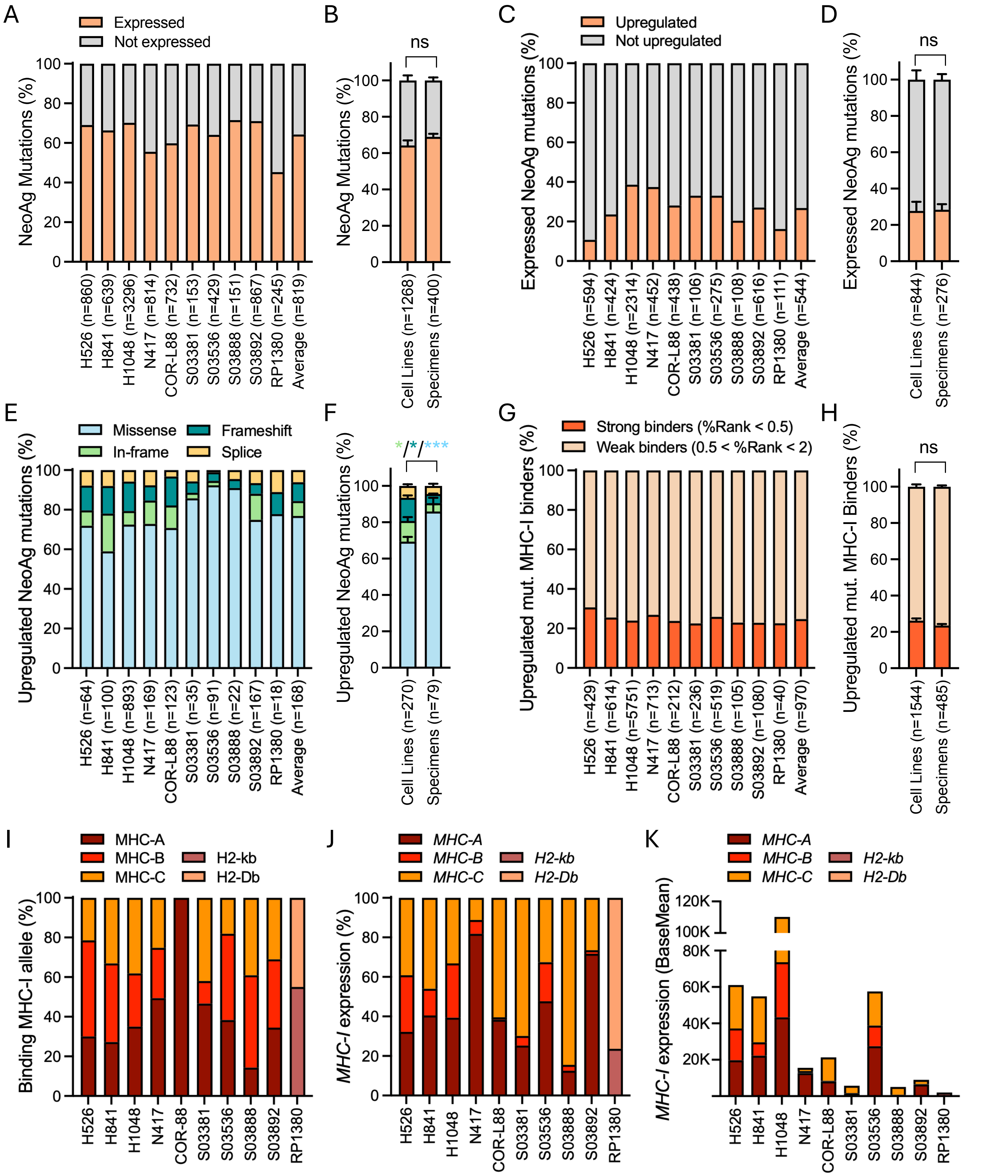

A
Expressed
Not expressed
NeoAg Mutations (%)
H526 (n=860)
H841 (n=639)
H1048 (n=3296)
N417 (n=814)
COR-L88 (n=732)
S03381 (n=153)
S03536 (n=429)
S03888 (n=151)
S03892 (n=867)
RP1380 (n=245)
Average (n=819)
B
ns
NeoAg Mutations (%)
Cell Lines (n=1268)
Specimens (n=400)
C
Upregulated
Not upregulated
Expressed NeoAg mutations (%)
H526 (n=594)
H841 (n=424)
H1048 (n=2314)
N417 (n=452)
COR-L88 (n=438)
S03381 (n=106)
S03536 (n=275)
S03888 (n=108)
S03892 (n=616)
RP1380 (n=111)
Average (n=544)
D
ns
Expressed NeoAg mutations (%)
Cell Lines (n=844)
Specimens (n=276)
E
Missense
Frameshift
In-frame
Splice
Upregulated NeoAg mutations (%)
H526 (n=64)
H841 (n=100)
H1048 (n=893)
N417 (n=169)
COR-L88 (n=123)
S03381 (n=35)
S03536 (n=91)
S03888 (n=22)
S03892 (n=167)
RP1380 (n=18)
Average (n=168)
F
//***
Upregulated NeoAg mutations (%)
Cell Lines (n=270)
Specimens (n=79)
G
Strong binders (%Rank < 0.5)
Weak binders (0.5 < %Rank < 2)
Upregulated mut. MHC-I binders (%)
H526 (n=429)
H841 (n=614)
H1048 (n=5751)
N417 (n=713)
COR-L88 (n=212)
S03381 (n=236)
S03536 (n=519)
S03888 (n=105)
S03892 (n=1080)
RP1380 (n=40)
Average (n=970)
H
ns
Upregulated mut. MHC-I Binders (%)
Cell Lines (n=1544)
Specimens (n=485)
I
MHC-A
MHC-B
MHC-C
H2-kb
H2-Db
Binding MHC-I allele (%)
H526
H841
H1048
N417
COR-88
S03381
S03536
S03888
S03892
RP1380
J
MHC-A
MHC-B
MHC-C
H2-kb
H2-Db
MHC-I expression (%)
H526
H841
H1048
N417
COR-L88
S03381
S03536
S03888
S03892
RP1380
K
MHC-A
MHC-B
MHC-C
H2-kb
H2-Db
MHC-I expression (BaseMean)
120K
100K
80K
60K
40K
20K
H526
H841
H1048
N417
COR-L88
S03381
S03536
S03888
S03892
RP1380

Figure S11 – related to Figure 5. Inhibition of NMD in SCLC samples upregulates hundreds of predicted neoantigens derived from all mutation types. A-B) Proportion of mutated genes identified as expressed by transcriptomic sequencing for the indicated SCLC samples (**A**) and comparing of cell line *vs* patient data (**B**). Only potentially NeoAg mutations (missense, in-frame, frameshift and splice) were considered. **C-D)** Proportion of expressed mutated genes found to be upregulated upon SMG1i treatment in the indicated SCLC samples (**C**) and comparing cell line *vs* patient data (**D**). Only potentially NeoAg mutations were considered. **E-F)** Proportion of upregulated NeoAg mutations of different types (**E**) and comparing cell line *vs* patient data (**F**). **G-H)** Mutant MHC-I-binders predicted for upregulated NeoAg-encoding transcripts derived from missense, in-frame and frameshift mutations in the indicated SCLC samples (**G**) and comparing cell line *vs* patient data (**H**). **A-H** show proportion (%), with absolute numbers (n = abs) indicated for each sample. Average values (n = abs_mean) are also indicated for all samples, for all cell lines and for all patient specimens. **I)** Proportion of MHC-A/B/C molecules predicted to bind upregulated neoantigens from **G-H**. Note that not all six alleles per sample were available for the binding prediction (**Fig. S10E**). **J)** Proportion of *MHC-A/B/C* alleles expression determined by transcriptomic sequencing calculated from BaseMean expression values. **K)** *MHC-A/B/C* alleles expression (BaseMean) determined by transcriptomic sequencing. Error bars represent the SEM. ***P-value < 0.001; *P-value < 0.05; (Unpaired t-tests). See also **Fig. 5A, C** and **Table S5E.**

Figure S12

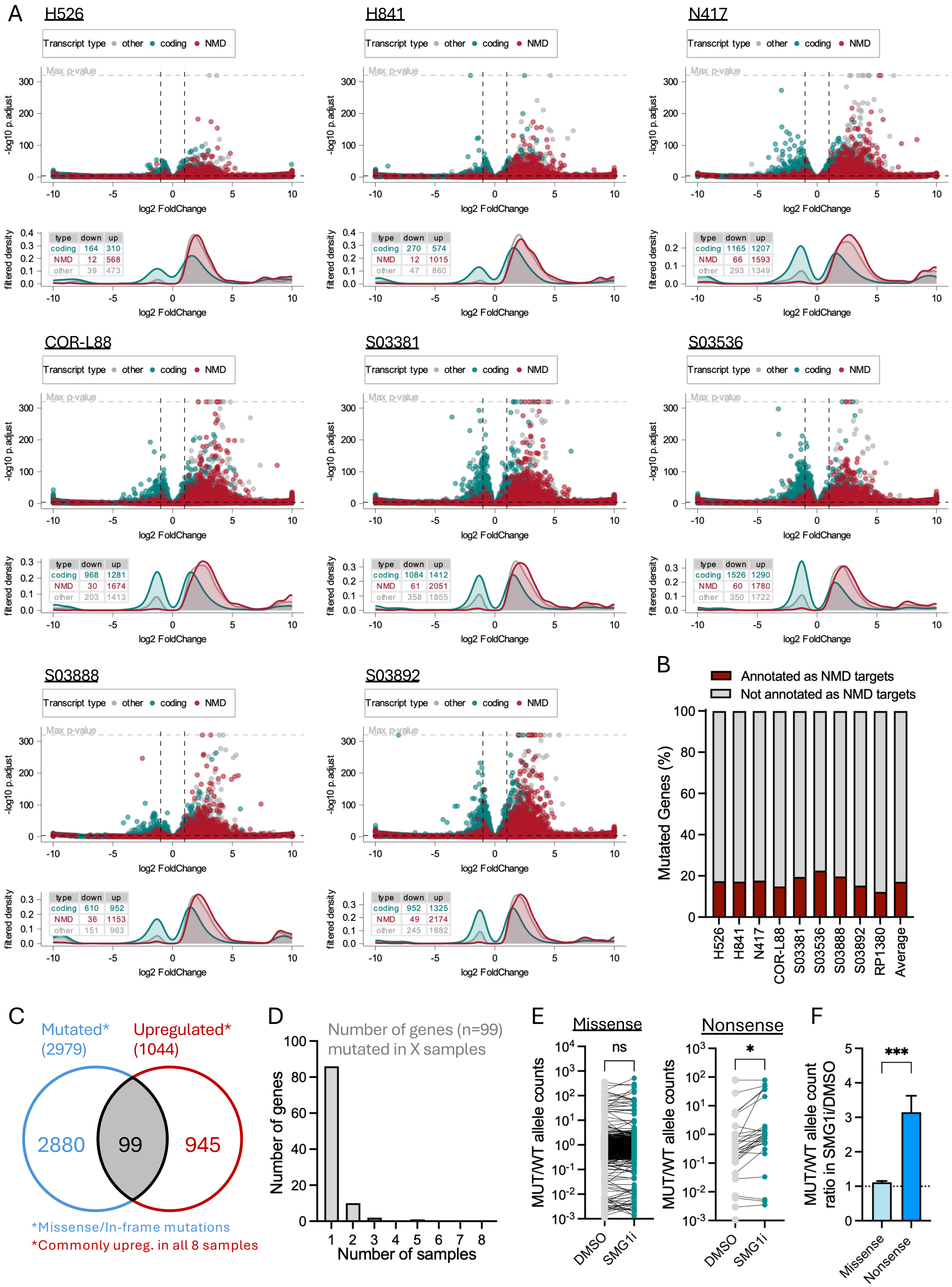
A
H526
H841
N417
COR-L88
S03381
S03536
S03888
S03892
Transcript type ● other ● coding ● NMD
Max p-value
-log10 p.adjust
log2 FoldChange
filtered density
H526: type down up; coding 164 310; NMD 12 568; other 39 473
H841: type down up; coding 270 574; NMD 12 1015; other 47 860
N417: type down up; coding 1165 1207; NMD 66 1593; other 293 1349
COR-L88: type down up; coding 968 1281; NMD 30 1674; other 203 1413
S03381: type down up; coding 1084 1412; NMD 61 2051; other 358 1855
S03536: type down up; coding 1526 1290; NMD 60 1780; other 350 1722
S03888: type down up; coding 610 952; NMD 36 1153; other 151 963
S03892: type down up; coding 952 1325; NMD 49 2174; other 245 1882
B
Annotated as NMD targets
Not annotated as NMD targets
Mutated Genes (%)
H526
H841
N417
COR-L88
S03381
S03536
S03888
S03892
RP1380
Average
C
Mutated* (2979)
Upregulated* (1044)
2880
99
945
*Missense/In-frame mutations
*Commonly upreg. in all 8 samples
D
Number of genes (n=99) mutated in X samples
Number of genes
Number of samples
E
Missense
Nonsense
ns
*
MUT/WT allele counts
DMSO
SMG1i
F
***
MUT/WT allele count ratio in SMG1i/DMSO
Missense
Nonsense

Figure S12 – related to Figure 5. NMD-mediated regulation of missense and in-frame mutated transcripts. A) Volcano plots accompanied by filtered densities showing significantly up or downregulated transcripts (Log2FC > |1| and Padj < 0.001) predicted as NMD targets (*NMD*, based on transcript GENCODE annotation as *nonsense-mediated decay* owed to the presence of a PTC at > 50 bp upstream of the next splice site) in comparison to transcripts annotated as *protein-coding* or *non-coding*. Note that transcripts annotated as *nonsense-mediated decay* can also be either protein-coding or non-coding, but the *nonsense-mediated decay* annotation prevails. **B)** Proportion of mutated genes for each sample annotated as NMD targets. **C)** Overlap between genes containing missense or in-frame mutations in at least one of the 8 tested samples (shown in **A-B),** and genes commonly upregulated in all 8 tested samples upon SMG1i treatment. **D)** Number of samples in which overlapping genes identified in **C** are found to have a specific missense or in-frame mutation. **E-F)** Split allele count analysis in 8 samples (shown in **A-B**) using matched exome and transcriptome data. The expression ratio of mutated to wild-type alleles (MUT/WT) is shown for all upregulated missense (non-PTC mutation) and nonsense (PTC-mutation) mutated mRNAs in DMSO and SMG1i conditions **(E)** and for the SMG1i/DMSO ratio in order to show the differential regulation of MUT alleles between missense and nonsense mutations.

Figure S13

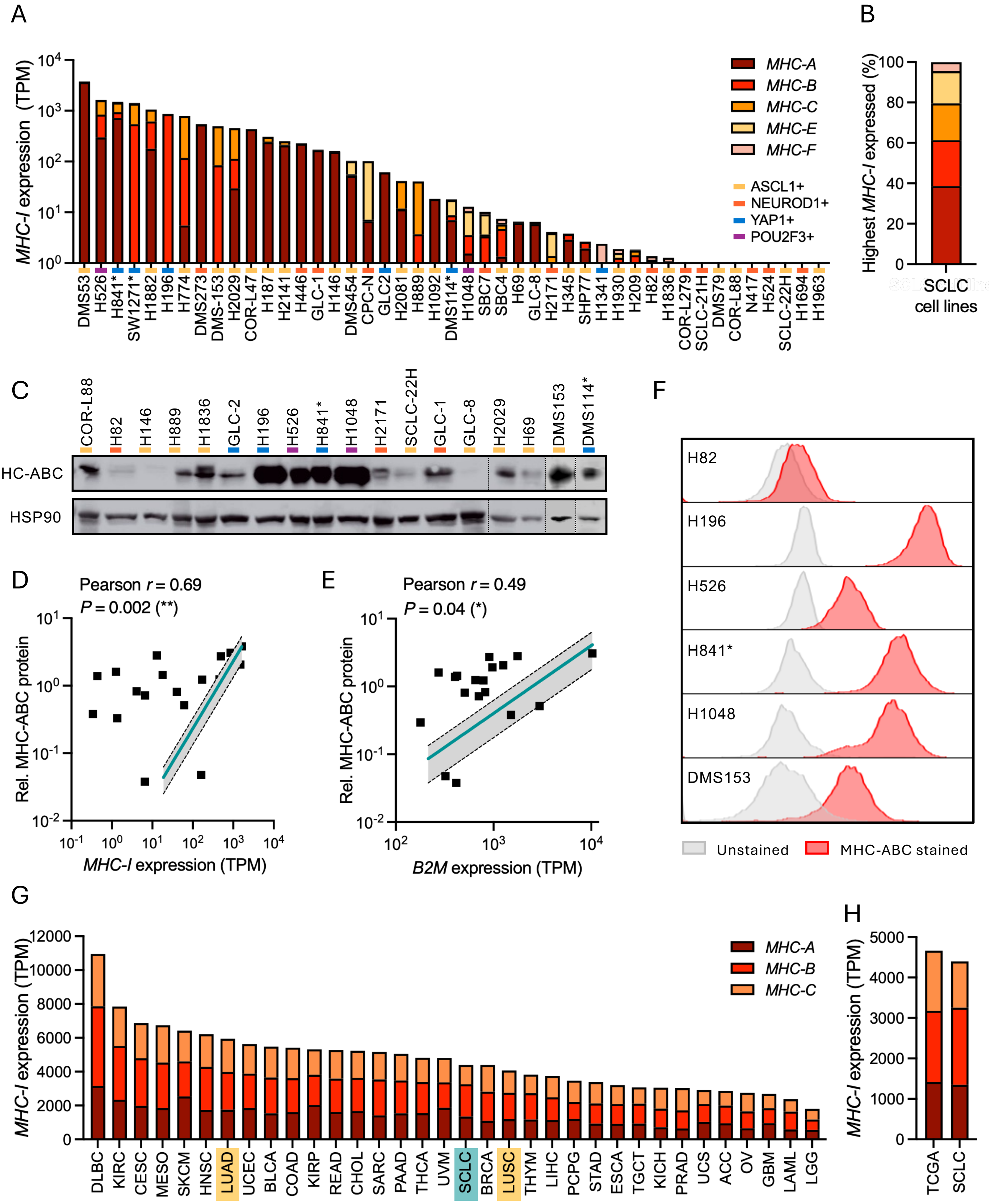
A
MHC-I expression (TPM)
MHC-A
MHC-B
MHC-C
MHC-E
MHC-F
ASCL1+
NEUROD1+
YAP1+
POU2F3+
DMS53 H526 H841* SW1271* H1882 H196 H774 DMS273 DMS-153 H2029 COR-L47 H187 H2141 H446 GLC-1 H146 DMS454 CPC-N GLC2 H2081 H889 H1092 DMS114* H1048 SBC7 SBC4 H69 GLC-8 H2171 H345 SHP77 H1341 H1930 H209 H82 H1836 COR-L279 SCLC-21H DMS79 COR-L88 N417 H524 SCLC-22H H1694 H1963
B
Highest MHC-I expressed (%)
SCLC cell lines
C
COR-L88 H82 H146 H889 H1836 GLC-2 H196 H526 H841* H1048 H2171 SCLC-22H GLC-1 GLC-8 H2029 H69 DMS153 DMS114*
MHC-ABC
HSP90
D
Pearson r = 0.69
P = 0.002 (**)
Rel. MHC-ABC protein
MHC-I expression (TPM)
E
Pearson r = 0.49
P = 0.04 (*)
Rel. MHC-ABC protein
B2M expression (TPM)
F
H82
H196
H526
H841*
H1048
DMS153
Unstained
MHC-ABC stained
G
MHC-I expression (TPM)
MHC-A
MHC-B
MHC-C
DLBC KIRC CESC MESO SKCM HNSC LUAD UCEC BLCA COAD KIRP READ CHOL SARC PAAD THCA UVM SCLC BRCA LUSC THYM LIHC PCPG STAD ESCA TGCT KICH PRAD UCS ACC OV GBM LAML LGG
H
MHC-I expression (TPM)
TCGA
SCLC

Figure S13 – related to Figure 5. Human SCLC samples express MHC-I. A) *MHC-I* expression (sum of *MHC-A/B/C/E/F*) determined by transcriptome sequencing of 45 human SCLC cell lines under steady state conditions (with cell lines recently reclassified as SMARCA-UT marked with * and neuroendocrine phenotype indicated: ASCL1+ and NEUROD1+ (NE^{High}); POU2F3 and YAP1+ (NE^{Low}). **B)** Proportion of most abundant *MHC-I* genes referring to the highest expressed *MHC-I* for each cell line determined by transcriptome sequencing. 80% of cell lines express mostly classical MHC-A/B/C molecules. Data is displayed following the color code depicted in panel A. **C)** Western blot showing protein levels of MHC-ABC in human SCLC cell lines under steady state conditions (n = 18; including recently reclassified SMARCA4-UT as indicated by * and neuroendocrine phenotype as described in **A**). **D-E)** Pearson correlation of *MHC-I* mRNA expression (**D**) or *B2M* mRNA expression (**E**), both derived from transcriptome sequencing data, with MHC-ABC protein levels relative to HSP90 protein levels quantified by densitometry analysis for western blots shown in panel **C**. **F)** Flow cytometry histograms for MHC-ABC surface expression in n = 6 human SCLC cell lines. Recent re-classifications as SMARCA4-UT are designated with *. H82 was chosen as a low MHC-ABC-expressing cell line and all other cell lines as high MHC-ABC, confirming expression levels and correlations depicted in previous panels **A-E**. **G)** *MHC-ABC* expression for SCLC and all cancers within the TCGA database. **H)** *MHC-ABC* expression in SCLC vs the average of all cancers within the TCGA database.

Figure S14

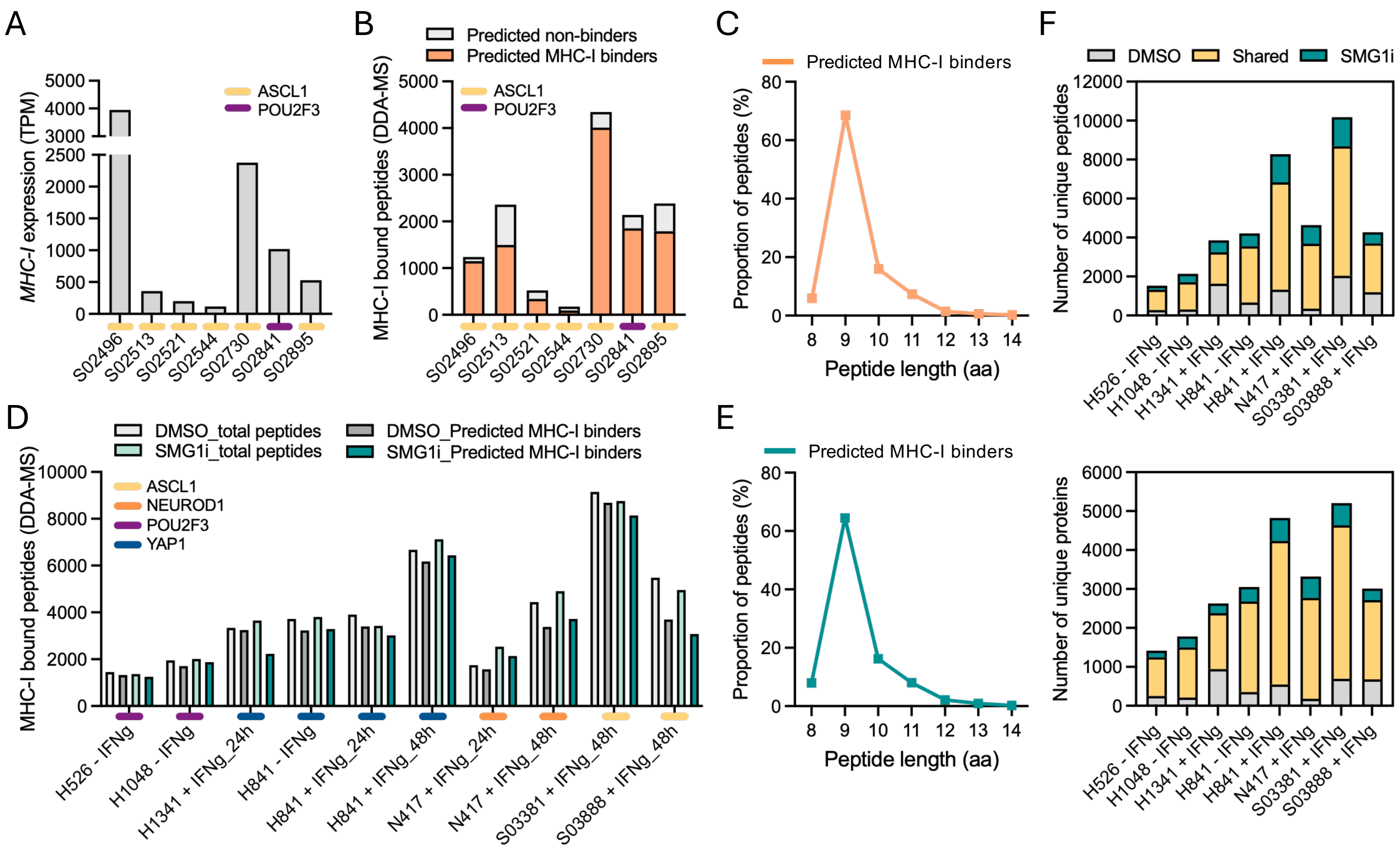

A
B
C
D
E
F
ASCL1
POU2F3
NEUROD1
YAP1
MHC-I expression (TPM)
S02496
S02513
S02521
S02544
S02730
S02841
S02895
Predicted non-binders
Predicted MHC-I binders
MHC-I bound peptides (DDA-MS)
Proportion of peptides (%)
Peptide length (aa)
DMSO_total peptides
SMG1i_total peptides
DMSO_Predicted MHC-I binders
SMG1i_Predicted MHC-I binders
H526 - IFNg
H1048 - IFNg
H1341 + IFNg_24h
H841 - IFNg
H841 + IFNg_24h
H841 + IFNg_48h
N417 + IFNg_24h
N417 + IFNg_48h
S03381 + IFNg_48h
S03888 + IFNg_48h
DMSO
Shared
SMG1i
Number of unique peptides
Number of unique proteins
H1341 + IFNg
H841 + IFNg
N417 + IFNg
S03381 + IFNg
S03888 + IFNg

Figure S14 – related to Figure 5. MHC-I immunopeptidomics in SCLC patients and cell lines of distinct molecular subtypes. A) *MHC-I* transcript expression (TPM sum of *MHC-A/B/C*) for n=7 SCLC patient specimens including NE^{High} and NE^{Low} subtypes. **B-C)** MHC-I immunopeptidomics obtained by data-dependent acquisition mass spectrometry (DDA-MS) from n=7 PDX-derived SCLC patient samples, including NE^{High} and NE^{Low} subtypes (see **Methods**). Immunoprecipitated peptides were subjected to MHC-I binding prediction using sample specific MHC-I alleles to assess immunoprecipitation purity (**B**), and to confirm the expected peptide length distribution of 8-14 aa for predicted MHC-I binders (**C**). **D-F)** MHC-I immunopeptidomics obtained by data-dependent acquisition mass spectrometry (DDA-MS) for n=7 SCLC cell lines (including n=2 patient-derived cellular models) of NE^{High} and NE^{Low} subtypes treated with DMSO (control) or SMG1i for NMD inhibition. Immunoprecipitated peptides were analyzed for MHC-I binding prediction using sample specific MHC-I alleles to assess immunoprecipitation purity (**D**) and confirm the expected peptide length distribution of 8-14 aa for predicted MHC-I binders (**E**). The fractions of DMSO-specific and SMG1i-specific peptides are a proof-of-concept for an altered MHC-I immunopeptidome following NMD inhibition (**F**).

Figure S15

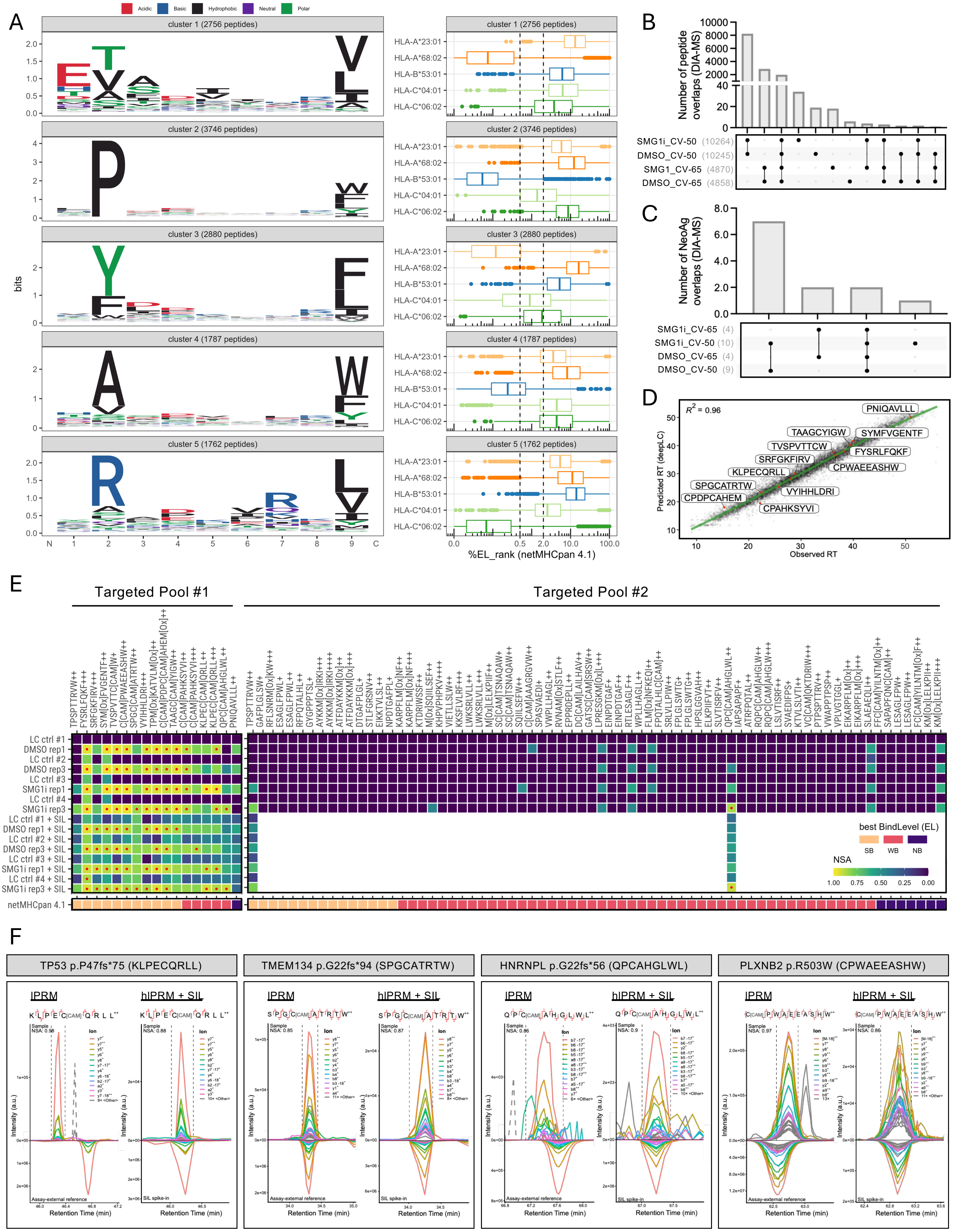

A
Acidic
Basic
Hydrophobic
Neutral
Polar
cluster 1 (2756 peptides)
cluster 2 (3746 peptides)
cluster 3 (2880 peptides)
cluster 4 (1787 peptides)
cluster 5 (1762 peptides)
bits
HLA-A*23:01
HLA-A*68:02
HLA-B*53:01
HLA-C*04:01
HLA-C*06:02
%EL_rank (netMHCpan 4.1)
B
Number of peptide overlaps (DIA-MS)
SMG1i_CV-50 (10264)
DMSO_CV-50 (10245)
SMG1_CV-65 (4870)
DMSO_CV-65 (4858)
C
Number of NeoAg overlaps (DIA-MS)
SMG1i_CV-65 (4)
SMG1i_CV-50 (10)
DMSO_CV-65 (4)
DMSO_CV-50 (9)
D
R^2 = 0.96
Predicted RT (deepLC)
Observed RT
PNIQAVLLL
TAAGCYIGW
SYMFVGENTF
TVSPVTTCW
FYSRLFQKF
SRFGKFIRV
CPWAEEASHW
KLPECQRLL
SPGCATRTW
VYIHHLDRI
CPDPCAHEM
CPAHKSYVI
E
Targeted Pool #1
Targeted Pool #2
LC ctrl #1
DMSO rep1
LC ctrl #2
DMSO rep3
LC ctrl #3
SMG1i rep1
LC ctrl #4
SMG1i rep3
LC ctrl #1 + SIL
DMSO rep1 + SIL
LC ctrl #2 + SIL
DMSO rep3 + SIL
LC ctrl #3 + SIL
SMG1i rep1 + SIL
LC ctrl #4 + SIL
SMG1i rep3 + SIL
netMHCpan 4.1
best BindLevel (EL)
SB
WB
NB
NSA
F
TP53 p.P47fs*75 (KLPECQRLL)
TMEM134 p.G22fs*94 (SPGCATRTW)
HNRNPL p.G22fs*56 (QPCAHGLWL)
PLXNB2 p.R503W (CPWAEEASHW)
IPRM
hIPRM + SIL
Intensity (a.u.)
Retention Time (min)
Assay-external reference
SIL spike-in

Figure S15 – related to Figure 5. MHC-I immunopeptidomics and targeted proteomics reveal upregulation of presented neoantigens upon NMD inhibition in SCLC. A) Analysis for sequence motif enrichment (**left**) in MHC-I binders identified by MHC-I immunopeptidomics via data-independent acquisition mass spectrometry (DIA-MS) and NetMHCpan EL 4.1 binding predictions of peptides constituting the respective motif (**right**). **B-C)** UpSet plots showing number and overlaps for overall peptides (**B**) and *bona fide* neoantigens (**C**) captured by MHC-I immunopeptidomics via DIA-MS using two different compensation voltages (CV, -50 and -65). **D**) Correlation of observed and predicted retention times (RT) for all peptides captured by MHC-I immunopeptidomics via DIA-MS, with *bona fide* neoantigens highlighted. **E)** Targeted immunopeptidomics on MHC-I immunoprecipitated samples for the validation of neoantigen peptides identified via DIA-MS (n = 13) and to assess novel neoantigen discovery of predicted neoantigens (n = 58, see **Methods**). NSA = Normalized Spectral Angle; SB = strong binders, WB = weak binders and NB = non-binder based on MHC-I binding predictions via NetMHCpan EL 4.1. **F)** Extracted ion chromatograms from targeted immunopeptidomics (**top**) and comparison with assay-external reference signal (IPRM) or SIL spike-in signal (hIPRM+SIL) for three SMG1i-upregulated neoantigens identified via unbiased DIA-MS approach (Targeted Pool #1) and one SMG1i-upregulated neoantigen discovered by the targeted approach, as part of the predicted frameshift neoantigen pool (Targeted Pool #2). See also **Table S5F-G.**

Figure S16

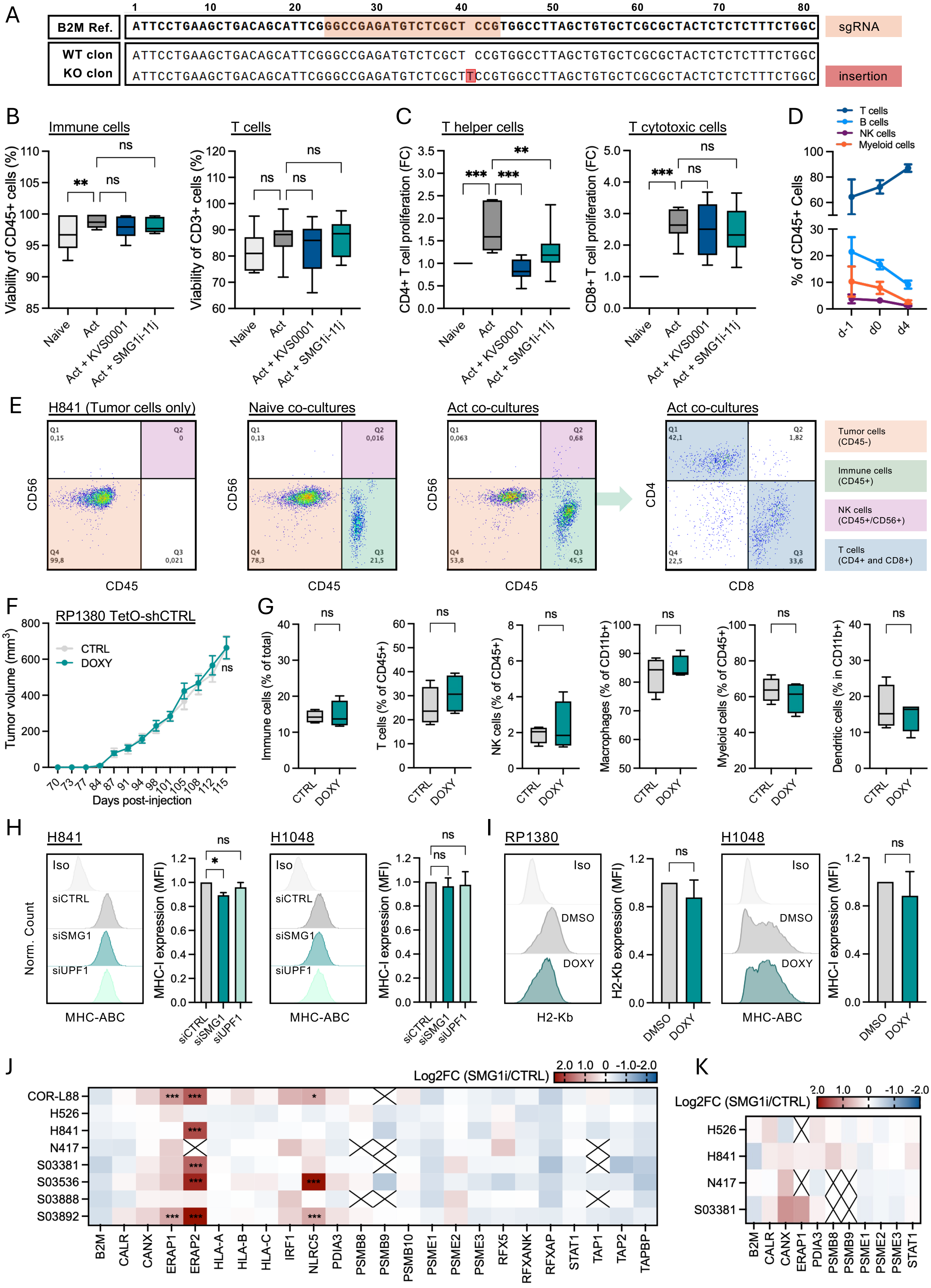

A
B2M Ref.
ATTCCTGAAGCTGACAGCATTCGGGCCGAGATGTCTCGCT CCGTGGCCTTAGCTGTGCTCGCGCTACTCTCTCTTTCTGGC
sgRNA
WT clon
KO clon
ATTCCTGAAGCTGACAGCATTCGGGCCGAGATGTCTCGCT CCGTGGCCTTAGCTGTGCTCGCGCTACTCTCTCTTTCTGGC
ATTCCTGAAGCTGACAGCATTCGGGCCGAGATGTCTCGCTTCCGTGGCCTTAGCTGTGCTCGCGCTACTCTCTCTTTCTGGC
insertion
B
Immune cells
Viability of CD45+ cells (%)
T cells
Viability of CD3+ cells (%)
C
T helper cells
CD4+ T cell proliferation (FC)
T cytotoxic cells
CD8+ T cell proliferation (FC)
Naive
Act
Act + KVS0001
Act + SMG1i-11j
D
T cells
B cells
NK cells
Myeloid cells
% of CD45+ Cells
d-1
d0
d4
E
H841 (Tumor cells only)
Naive co-cultures
Act co-cultures
Act co-cultures
CD56
CD45
CD4
CD8
Tumor cells (CD45-)
Immune cells (CD45+)
NK cells (CD45+/CD56+)
T cells (CD4+ and CD8+)
F
RP1380 TetO-shCTRL
CTRL
DOXY
Tumor volume (mm³)
Days post-injection
G
Immune cells (% of total)
T cells (% of CD45+)
NK cells (% of CD45+)
Macrophages (% of CD11b+)
Myeloid cells (% of CD45+)
Dendritic cells (% in CD11b+)
H
H841
H1048
Norm. Count
Iso
siCTRL
siSMG1
siUPF1
MHC-ABC
MHC-I expression (MFI)
I
RP1380
H1048
DMSO
DOXY
H2-Kb
H2-Kb expression (MFI)
MHC-I expression (MFI)
J
Log2FC (SMG1i/CTRL)
COR-L88
H526
H841
N417
S03381
S03536
S03888
S03892
B2M
CALR
CANX
ERAP1
ERAP2
HLA-A
HLA-B
HLA-C
IRF1
NLRC5
PDIA3
PSMB8
PSMB9
PSMB10
PSME1
PSME2
PSME3
RFX5
RFXANK
RFXAP
STAT1
TAP1
TAP2
TAPBP
K
Log2FC (SMG1i/CTRL)
H526
H841
N417
S03381

Figure S16 – related to Figure 6. Inhibition of NMD enhances SCLC immunogenicity *in vitro* and *in vivo*. A) DNA Sanger sequencing of the amplified B2M locus in representative H841 CRISPR/Cas9 WT and B2M-KO clones aligned to the reference sequence (NCBI, NG_012920.2) with sgRNA and the identified insertion mutation in H841 B2M-KO cells highlighted. **B-D)** Pharmacological NMD inhibition with either KVS0001 or SMG1i-11j compounds in healthy PBMC donors upon T cell artificial activation. (**B**) Proportion of viable CD45+ cells (**left**) and T cells (**right**) within PBMC populations after 4 days treatment with a T cell artificial activation cocktail (Act = CD3+CD28+IL-2) *vs* unstimulated conditions (Naive). (**C**) Proliferation of CD4+ (**left**) and CD8+ (**right**) T cells derived from T cell counts expressed as fold change (FC) for activated (Act) conditions relative to the unstimulated (Naive) control. (**D**) Proportion of T cells (CD3+), B cells (CD19+), NK cells (CD56+) and Myeloid cells (CD11b+) within PBMC populations (CD45+) from healthy donors directly after thawing (d-1), directly prior to stimulation (d0) with a T cell artificial activation cocktail (CD3+CD28+IL-2) and 4 days post-stimulation (d4). Graphs represent mean + SEM (n = 7). ***P<0,001; **P<0.01; *P<0.05; ns=non-significant (One-way ANOVA). **E)** Representative flow cytometry dot plots for the staining of CD45 *vs* CD56 (for NK cells) and CD8 *vs* CD4 (both for T cells) following 4 days co-culture of healthy donor PBMCs with WT H841 tumor cells. **F)** *In vivo* tumor growth in immunocompetent C57BL/6J mice transplanted with murine RP1380 TetO-shCTRL fed with normal or doxycycline-containing diet (n ≥ 4). **G)** Flow cytometry immunophenotyping of RP1380 TetO-shCTRL tumors harvested at the end of experiments shown in panel F (n ≥ 4). **H-I)** MHC-I surface expression quantified by flow cytometry in the indicated human and murine cell lines following genetic NMD inhibition via siRNA-mediated SMG1-KD and UPF1-KD (**H**) or doxycycline-inducible SMG1-KD (**I**). **J-K)** Heatmaps for transcript (**J**) and protein (**K**) expression of components of the MHC-I antigen presentation machinery, measured via transcriptome sequencing data (***Log2FC > |1|; Padj < 0.05) or whole cell DDA-MS proteomics (for all data points P > 0.05 ; ns), respectively.

Figure S17

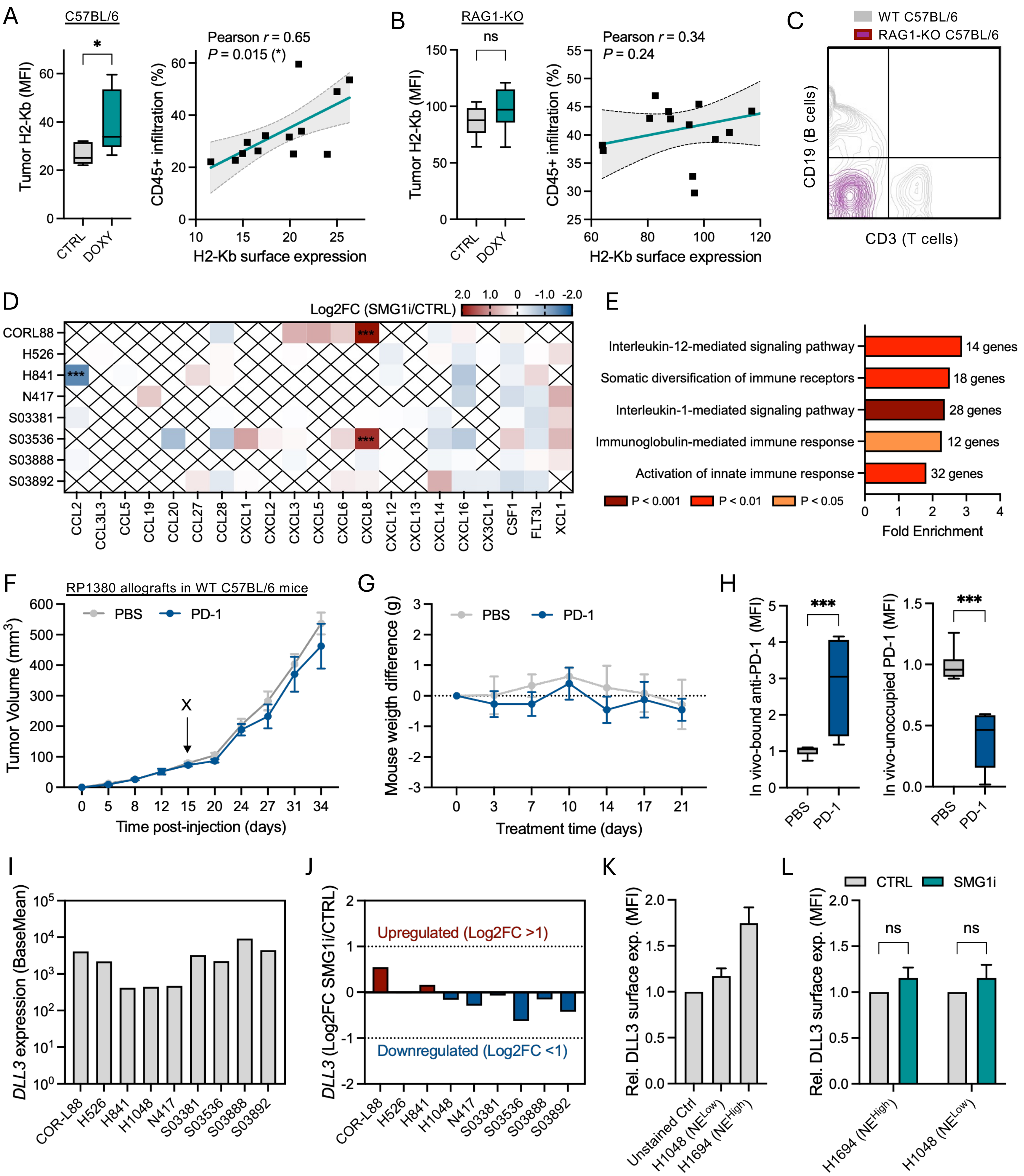

A
C57BL/6
Tumor H2-Kb (MFI)
CTRL
DOXY
Pearson r = 0.65
P = 0.015 (*)
CD45+ infiltration (%)
H2-Kb surface expression
B
RAG1-KO
ns
Pearson r = 0.34
P = 0.24
C
WT C57BL/6
RAG1-KO C57BL/6
CD19 (B cells)
CD3 (T cells)
D
Log2FC (SMG1i/CTRL)
CORL88
H526
H841
N417
S03381
S03536
S03888
S03892
CCL2
CCL3L3
CCL5
CCL19
CCL20
CCL27
CCL28
CXCL1
CXCL2
CXCL3
CXCL5
CXCL6
CXCL8
CXCL12
CXCL13
CXCL14
CXCL16
CX3CL1
CSF1
FLT3L
XCL1
E
Interleukin-12-mediated signaling pathway
Somatic diversification of immune receptors
Interleukin-1-mediated signaling pathway
Immunoglobulin-mediated immune response
Activation of innate immune response
14 genes
18 genes
28 genes
12 genes
32 genes
P < 0.001
P < 0.01
P < 0.05
Fold Enrichment
F
RP1380 allografts in WT C57BL/6 mice
PBS
PD-1
Tumor Volume (mm³)
Time post-injection (days)
G
Mouse weigth difference (g)
Treatment time (days)
H
In vivo-bound anti-PD-1 (MFI)
In vivo-unoccupied PD-1 (MFI)
I
DLL3 expression (BaseMean)
COR-L88
H526
H841
H1048
N417
S03381
S03536
S03888
S03892
J
DLL3 (Log2FC SMG1i/CTRL)
Upregulated (Log2FC >1)
Downregulated (Log2FC <1)
K
Rel. DLL3 surface exp. (MFI)
Unstained Ctrl
H1048 (NE^Low)
H1694 (NE^High)
L
CTRL
SMG1i
H1694 (NE^High)
H1048 (NE^Low)

Figure S17 – related to Figure 6. NMD inhibition modulates key determinants of anti-tumor immunity and immunotherapy response. **A)** *In vivo* levels of tumor-specific MHC-I (H2-Kb) surface expression in control (CTRL) vs NMD-inhibited (DOXY) murine RP1380 TetO-shSMG1 tumors grown subcutaneously in C57BL/6 mice (**left**), accompanied by data correlation analysis of tumor-specific H2-Kb surface expression with the levels of tumor immune cell infiltration (CD45+) measured by flow cytometry (**right**). **B**) *In vivo* levels of tumor-specific MHC-I (H2-Kb) surface expression in control (CTRL) vs NMD-inhibited (DOXY) RP1380 TetO-shSMG1 tumors grown subcutaneously in RAG1-KO C57BL/6 mice (**left**), accompanied by data correlation analysis of tumor-specific H2-Kb surface expression with the levels of tumor immune cell infiltration (CD45+) measured by flow cytometry (**right**). **C)** Representative flow cytometry contour plot showing T cells (CD45+/CD3+) and B cells (CD45+/CD19+) in blood from WT C57BL/6 and RAG1-KO C57BL/6 mice. **D)** Transcript levels of tumor-expressed chemokines in the indicated cell lines and patient-derived primary cells following 24 h SMG1i treatment (Log2FC, **Table S3**). ***Log2FC > |1|; Padj < 0.05. **E)** Top 5 immune-related signatures identified by pathway enrichment analysis of transcriptome sequencing data of n=8 SCLC samples treated with DMSO *vs* SMG1i. Input transcripts defined as commonly upregulated (Log2FC > 1 and Padj < 0.05) upon SMG1i treatment in at least 6 out of 8 samples (See **Tables S3** and **S4**). **F-H)** Tumor growth of RP1380 tumor allografts in C57BL/6J mice treated with PBS or anti-PD-1 under normal diet conditions serving as a control experiment for **Fig. 6J**. Body weight was monitored over three weeks of treatment (**G**). Target engagement of therapeutic anti-PD-1 on CD45+ tumor-infiltrating cells was measured by flow cytometry at endpoint using either a labelled secondary antibody (**H, left**) or a competing labelled anti-PD-1 antibody (**H, right**) (see **Methods**). **I)** *DLL3* transcript expression levels determined by transcriptome sequencing data (BaseMean, **Table S3**) in the indicated cell lines and patient-derived primary cells. **J)** Regulation of *DLL3* transcript expression following 24h SMG1i treatment in the indicated cell lines and patient-derived primary cells (Log2FC, **Table S3**). **K)** DLL3 surface expression measured by flow cytometry in representative NE^{High} and NE^{Low} SCLC cell lines, showing expectedly higher expression in NE^{High} cells. **L)** Flow cytometry data for DLL3 surface expression following 48 h SMG1i treatment in representative NE^{High} and NE^{Low} cell lines.
