# Supplementary material for: A targetable dependency on nonsense-mediated decay for cellular homeostasis and immune control in small cell lung cancer
Source: Mol Cancer. 2026 Aug 27;25:210. doi: 10.1186/s12943-026-02750-2 (PMC13551691; doi:10.1186/s12943-026-02750-2)

Uncropped western blot images:

Conditions and marker molecular weights are indicated in blue; probed antibodies are indicated in pink. For more information, please refer to corresponding figure legends and Methods section.

Figure 1C:

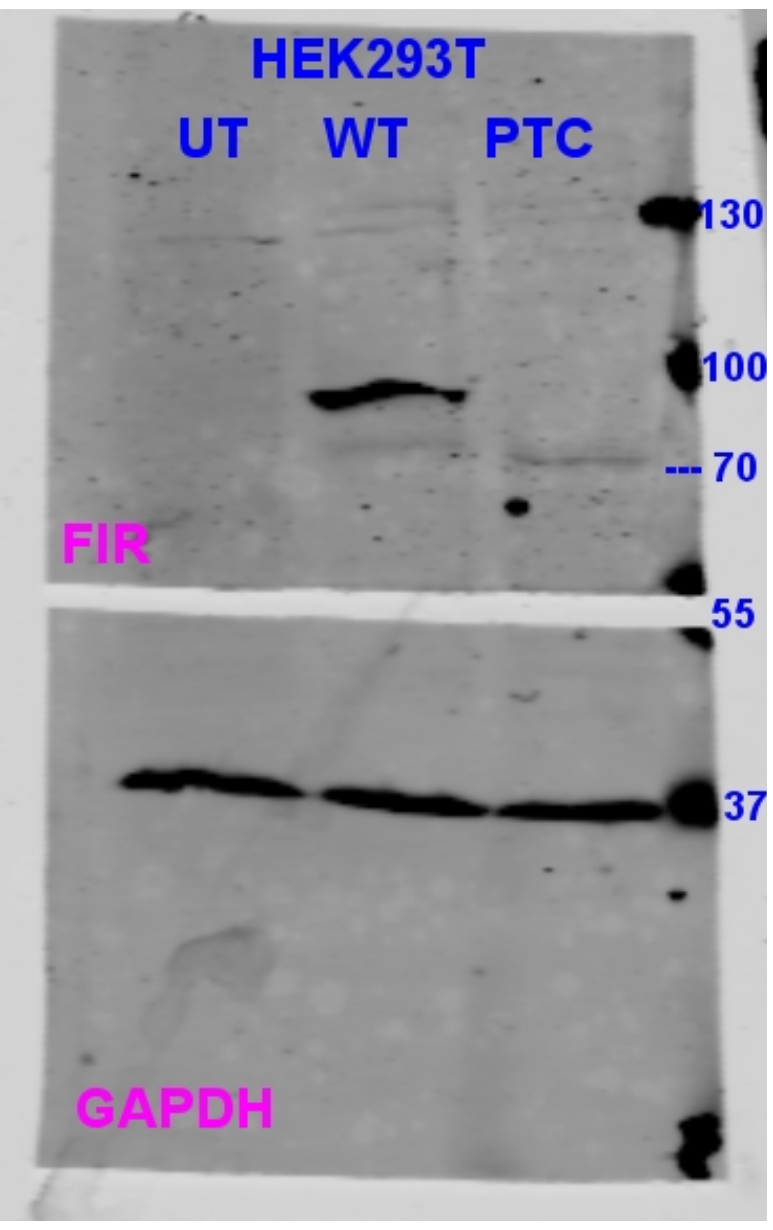

Figure 2S:

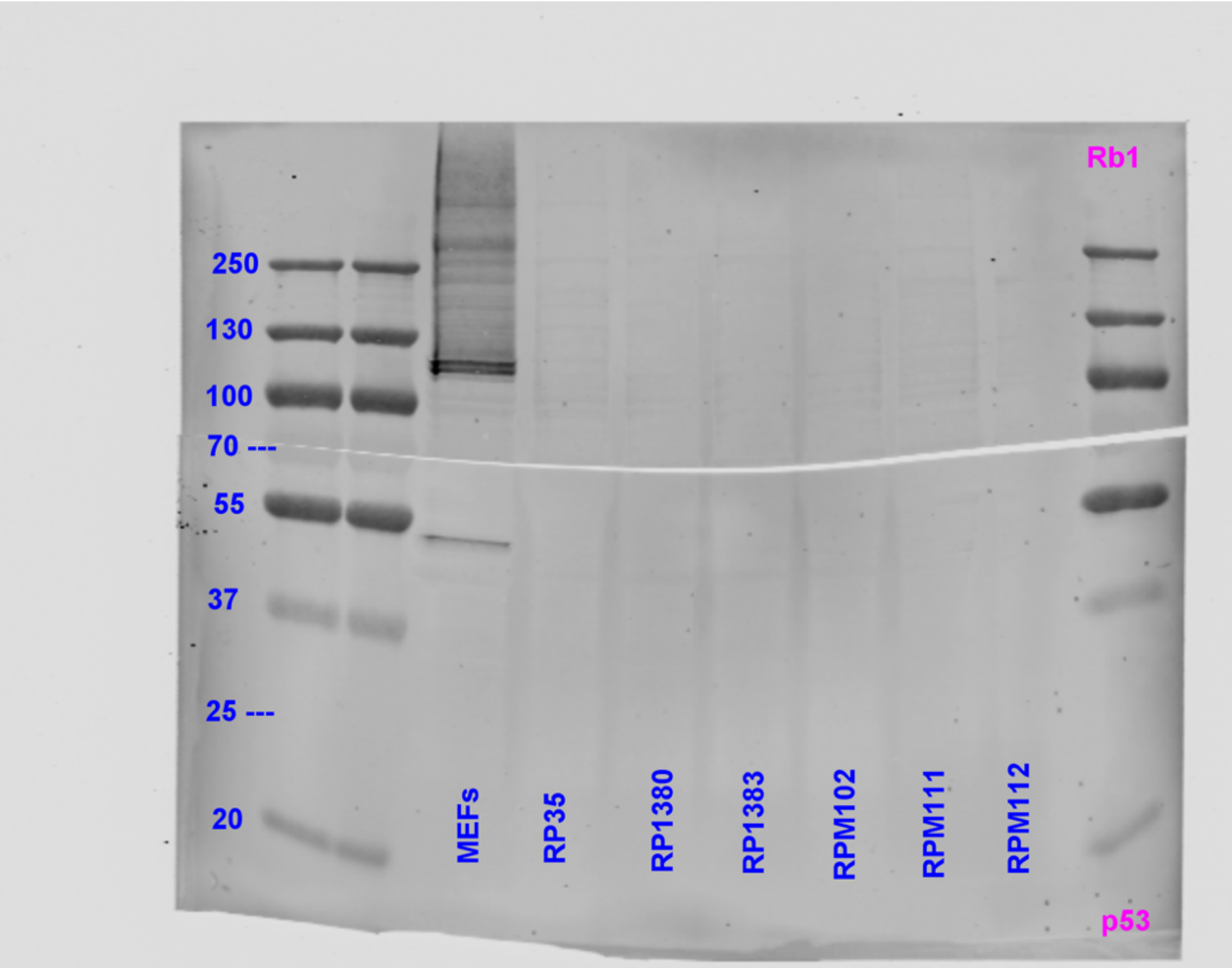

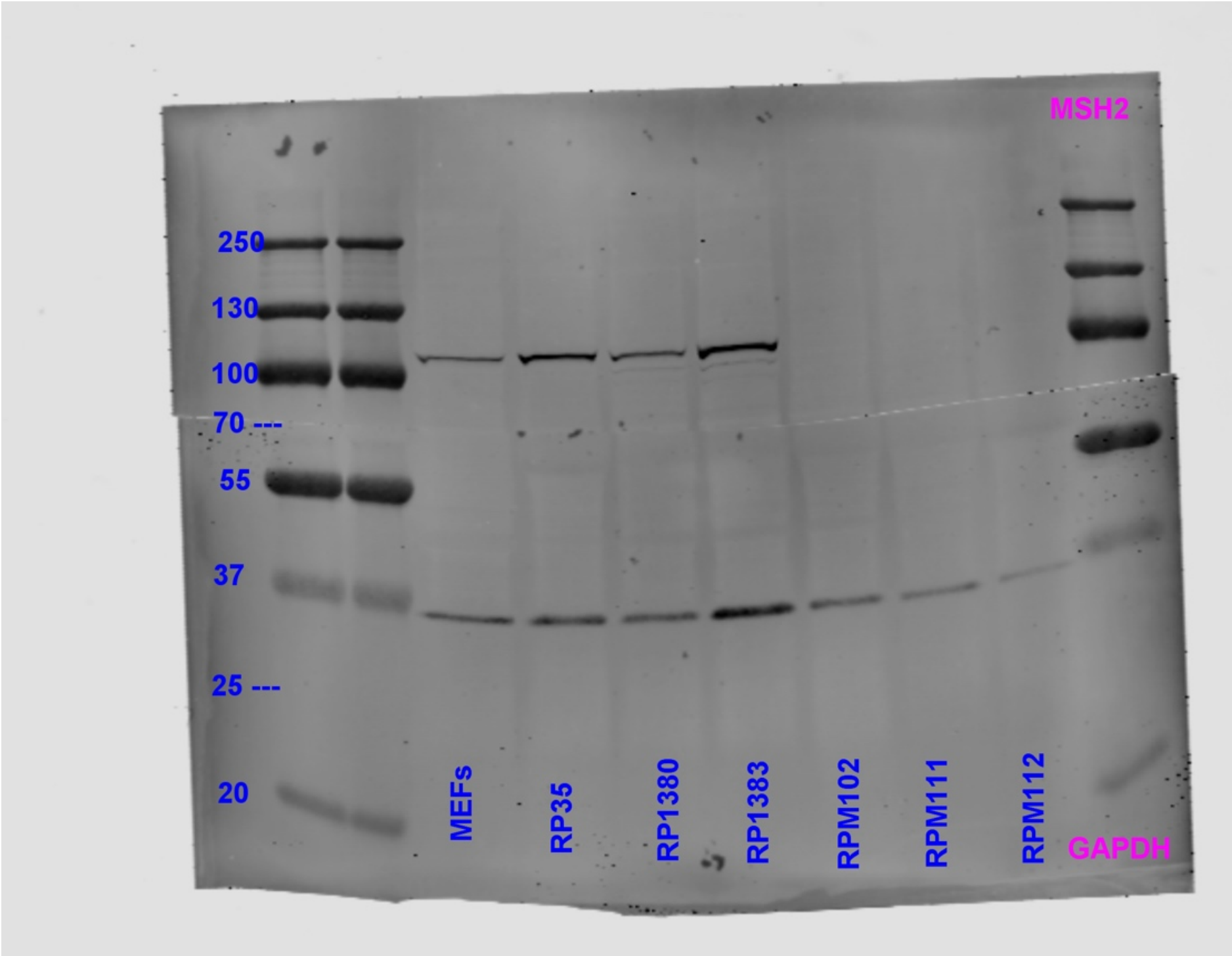
MSH2
250
130
100
70 ---
55
37
25 ---
20
MEFs
RP35
RP1380
RP1383
RPM102
RPM111
RPM112
GAPDH

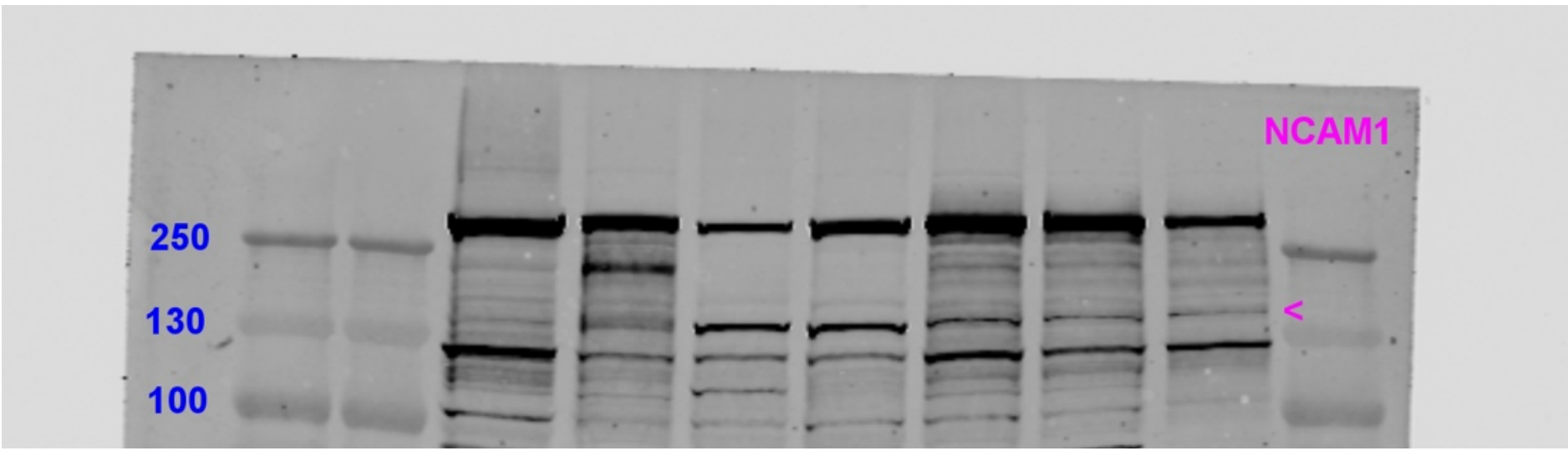
NCAM1
250
130
100

Figure 3C:

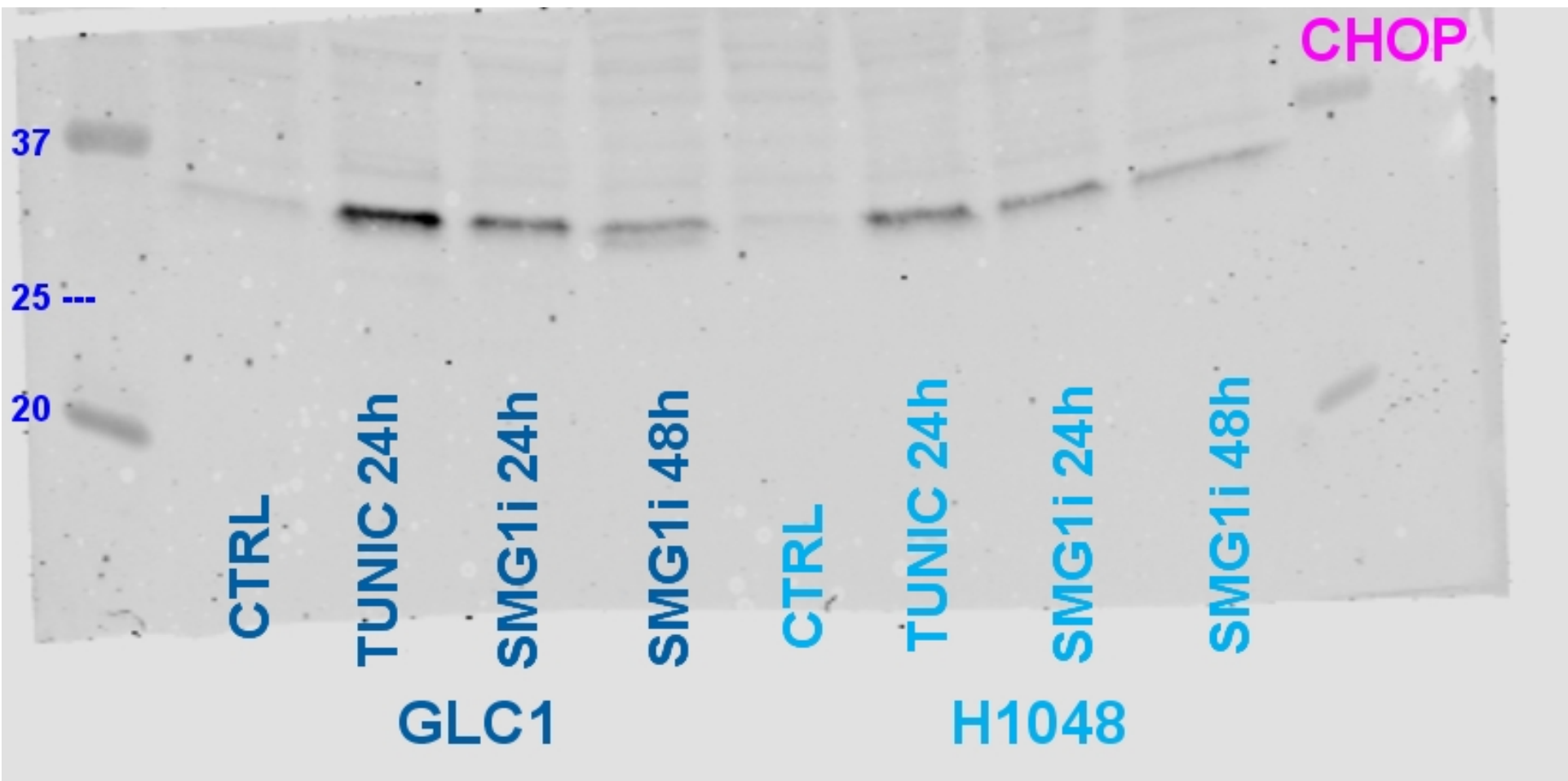
CHOP
37
25 ---
20
CTRL
TUNIC 24h
SMG1i 24h
SMG1i 48h
CTRL
TUNIC 24h
SMG1i 24h
SMG1i 48h
GLC1
H1048

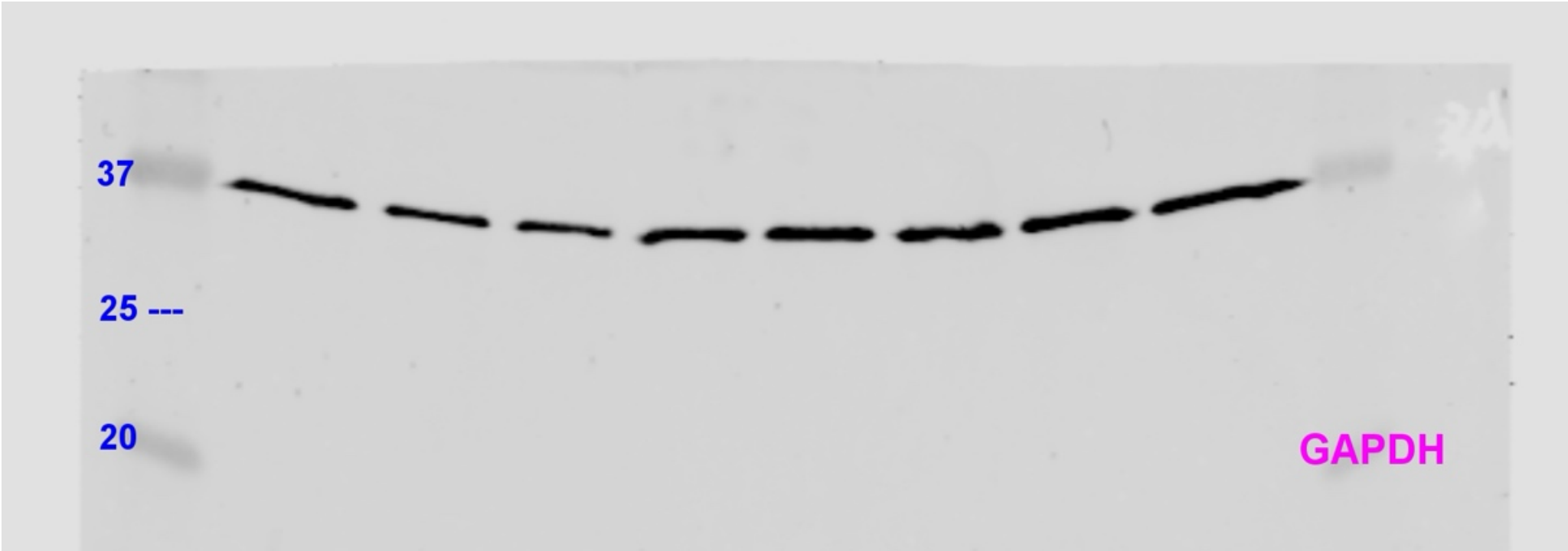
37
25 ---
20
GAPDH

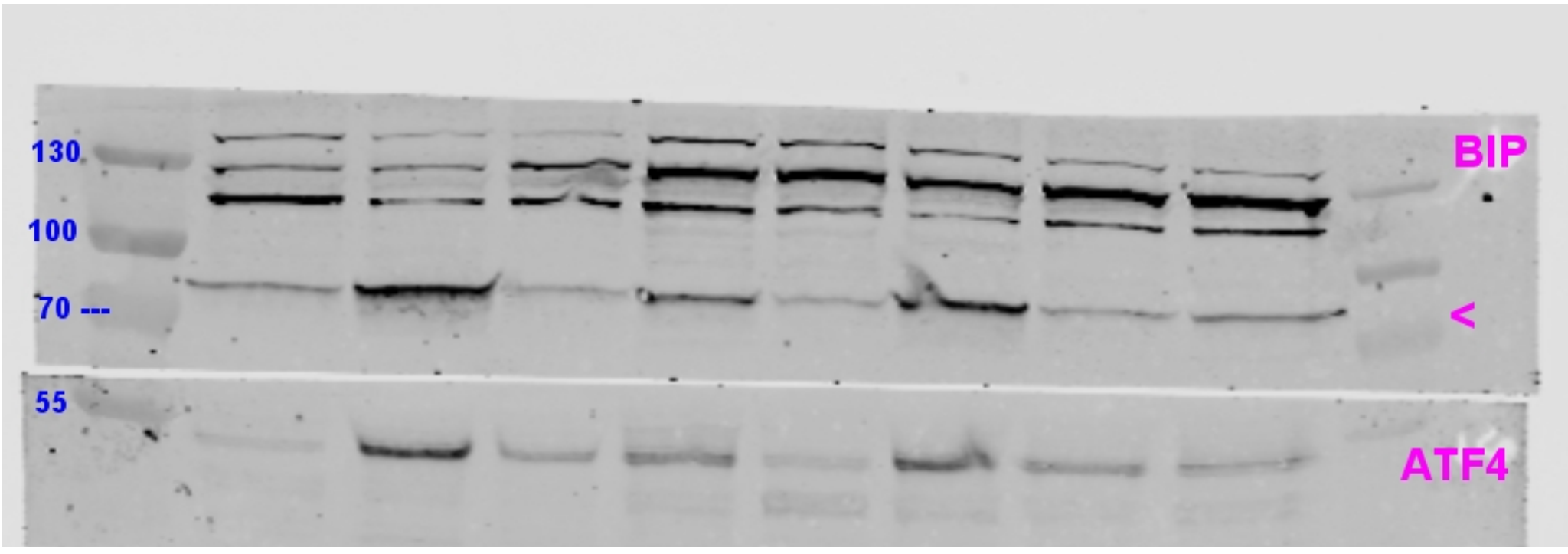
130
100
70 ---
55
BIP
<
ATF4

Figure 3D:

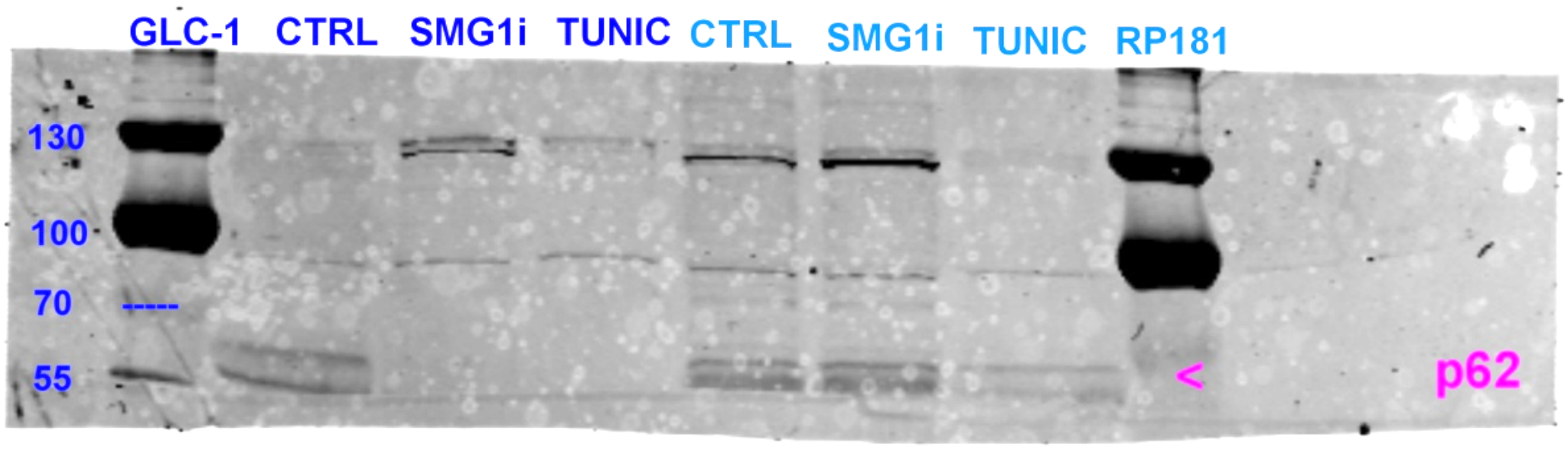
GLC-1 CTRL SMG1i TUNIC CTRL SMG1i TUNIC RP181
130
100
70
55
<
p62

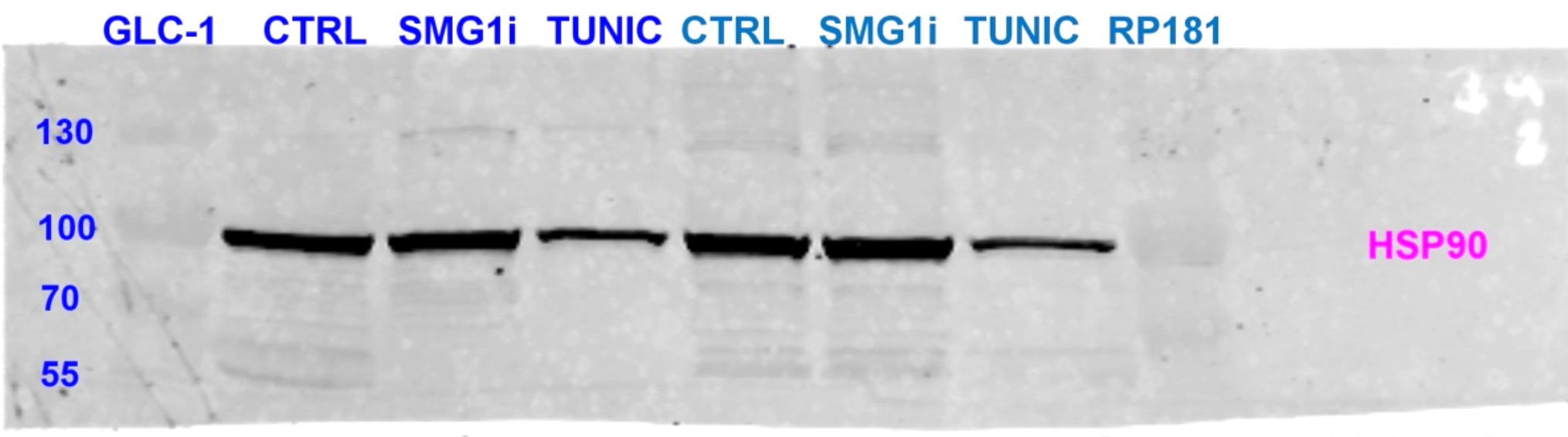
GLC-1 CTRL SMG1i TUNIC CTRL SMG1i TUNIC RP181
130
100
70
55
HSP90

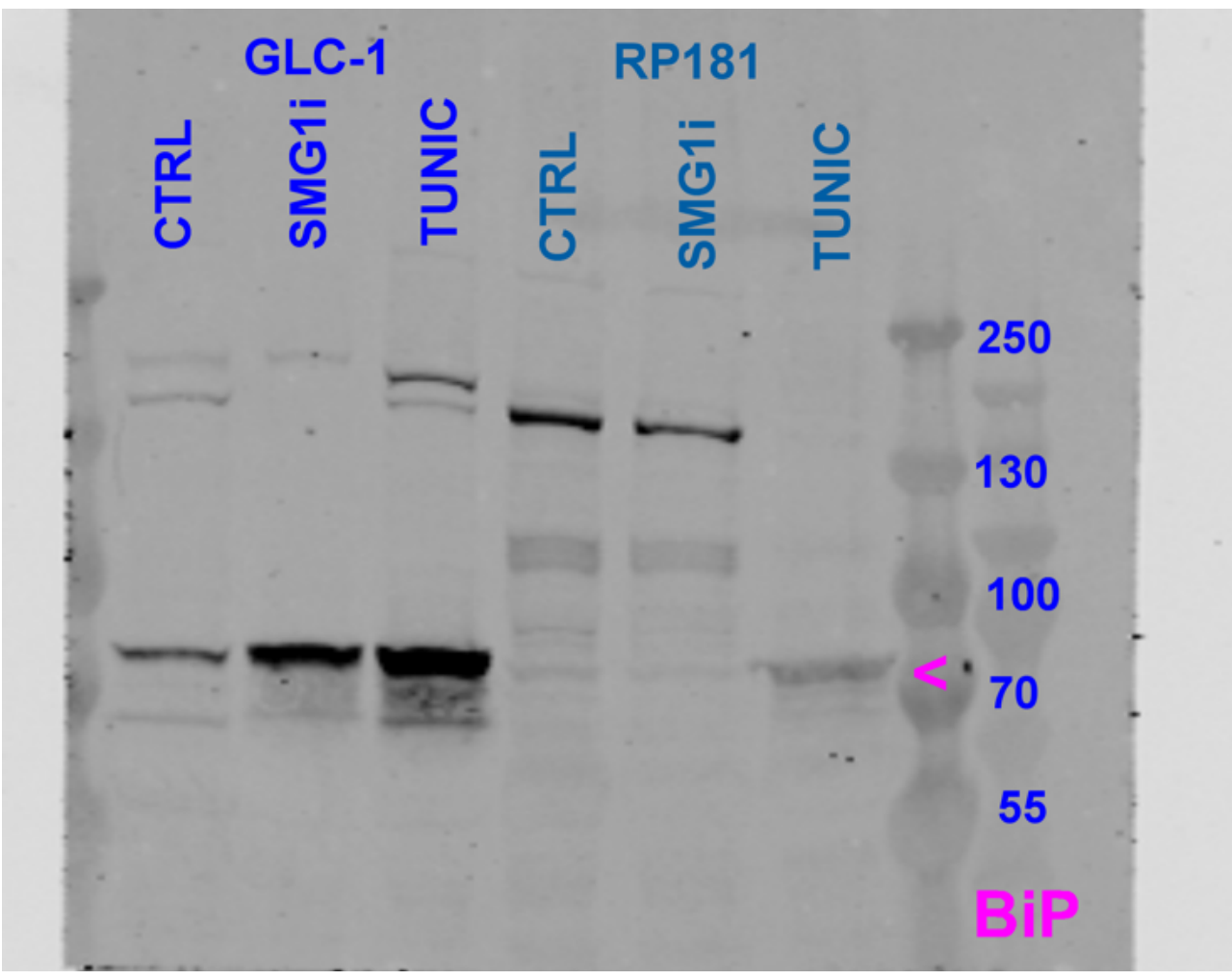

*Run on a separate blot due to close migration of BiP and p62 (both rabbit antibodies)

Figure 5E:

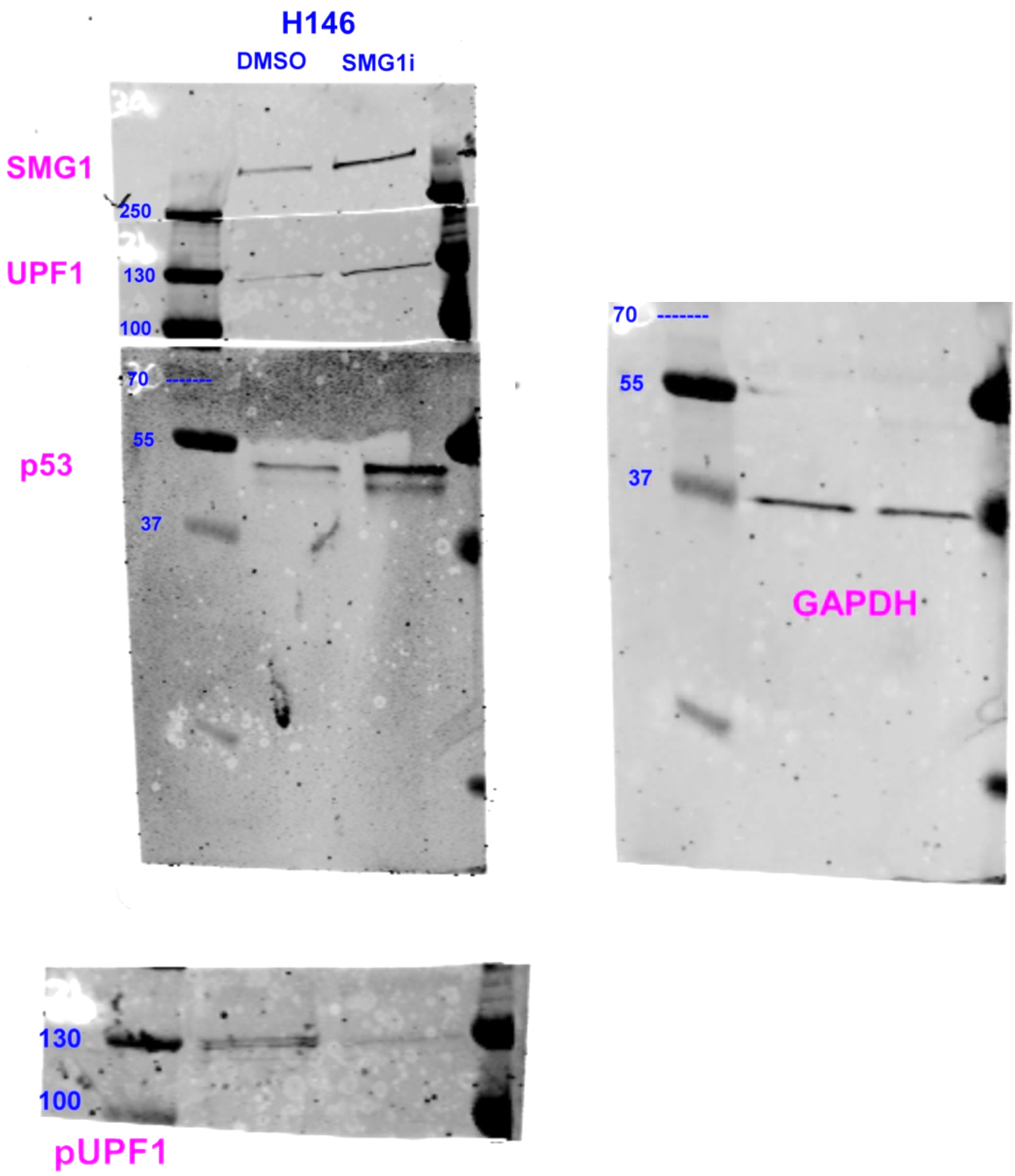

Figure 6C:

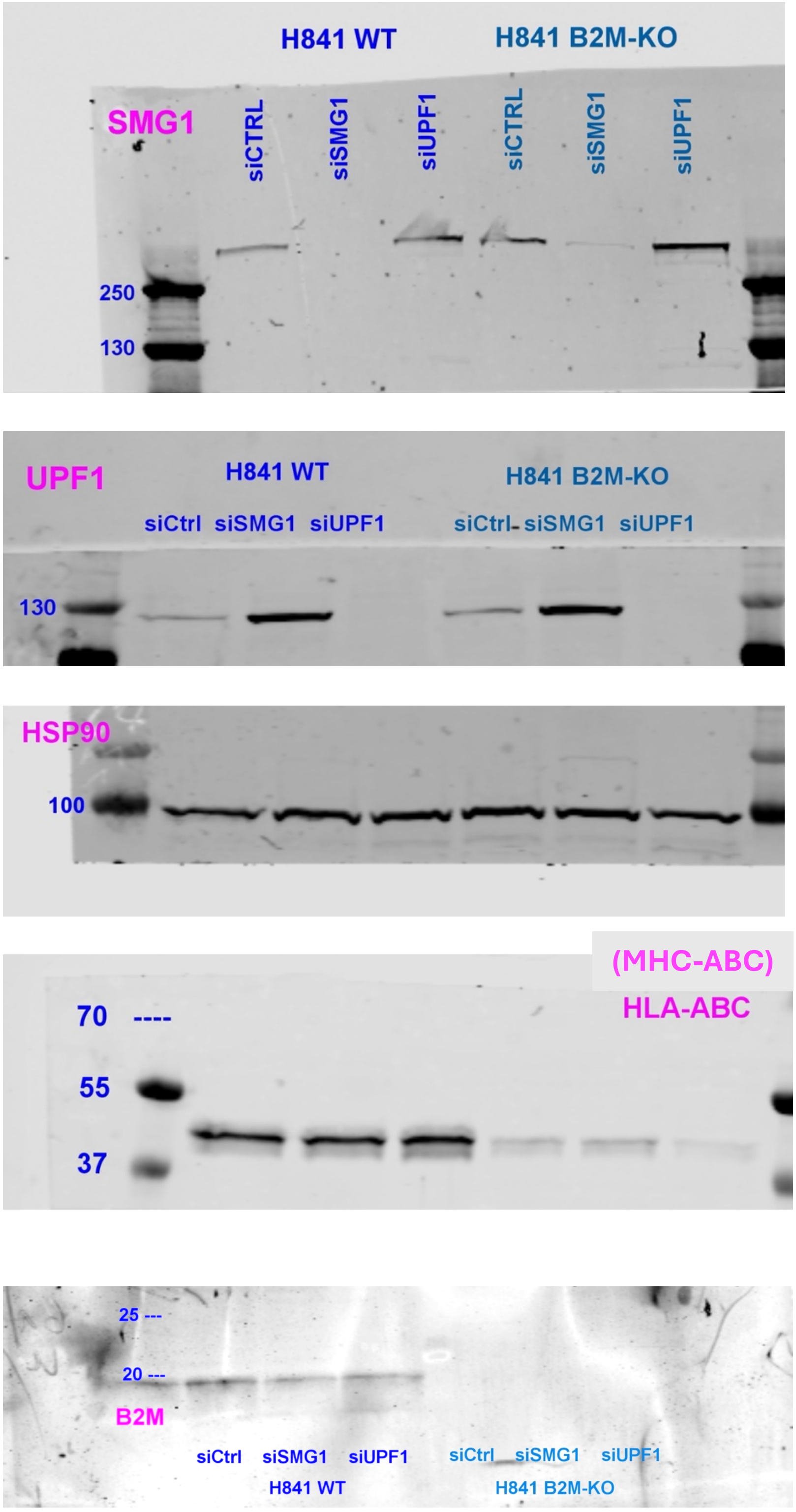

*Run on a separate blot because B2M is very small and required different transfer conditions

Figure S1F

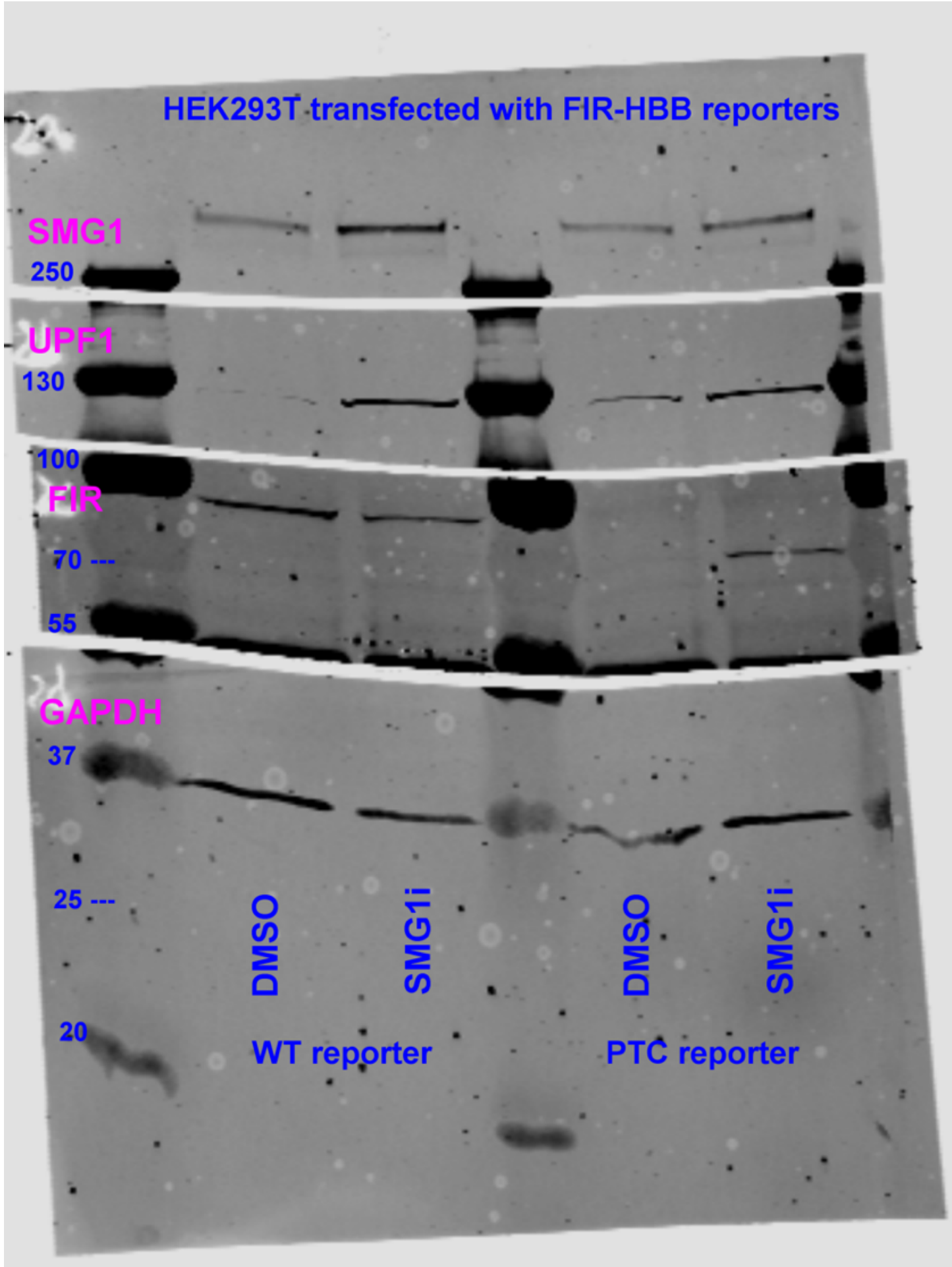

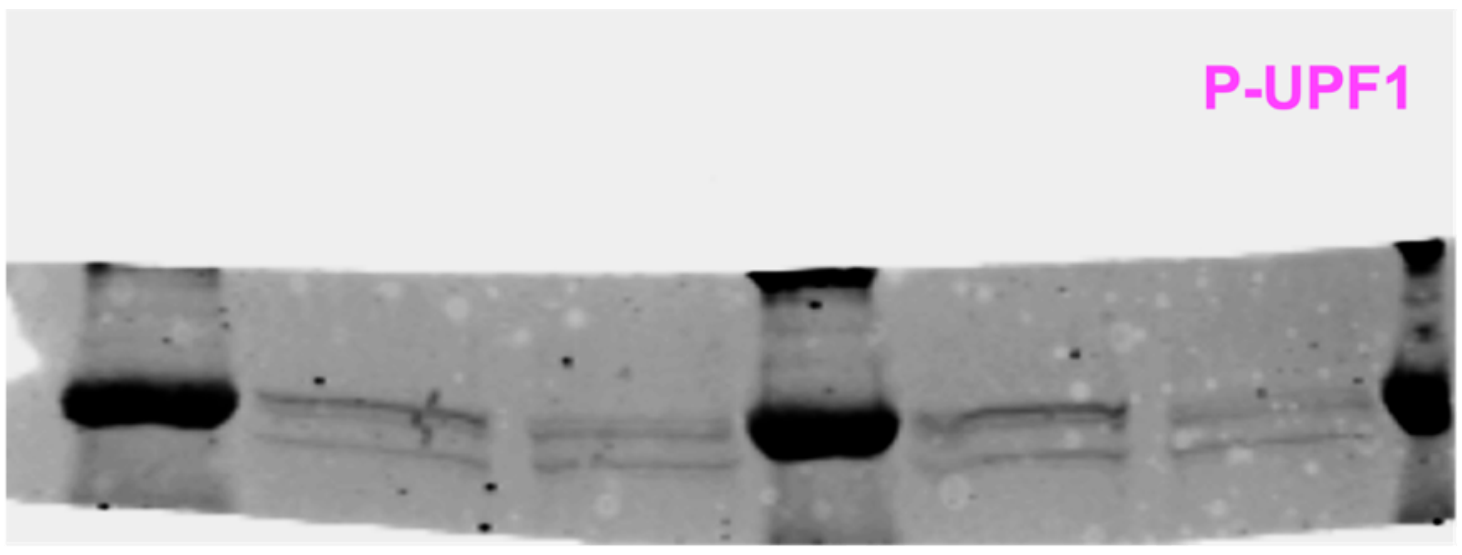

Figure S2B_H526

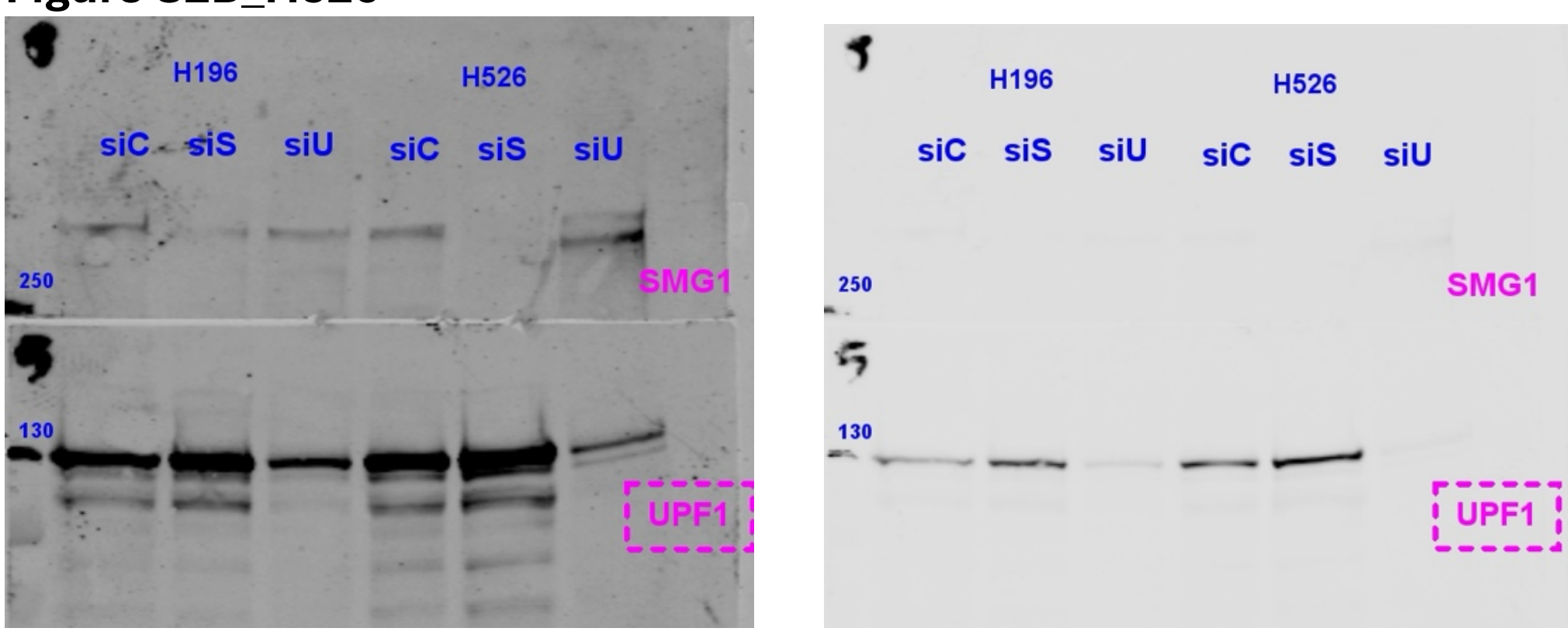

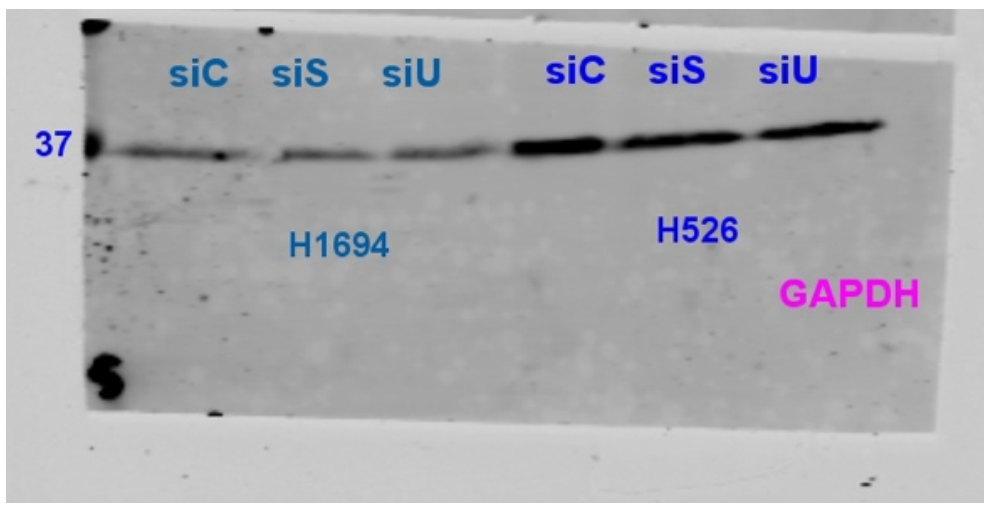

Figure S2B_H841

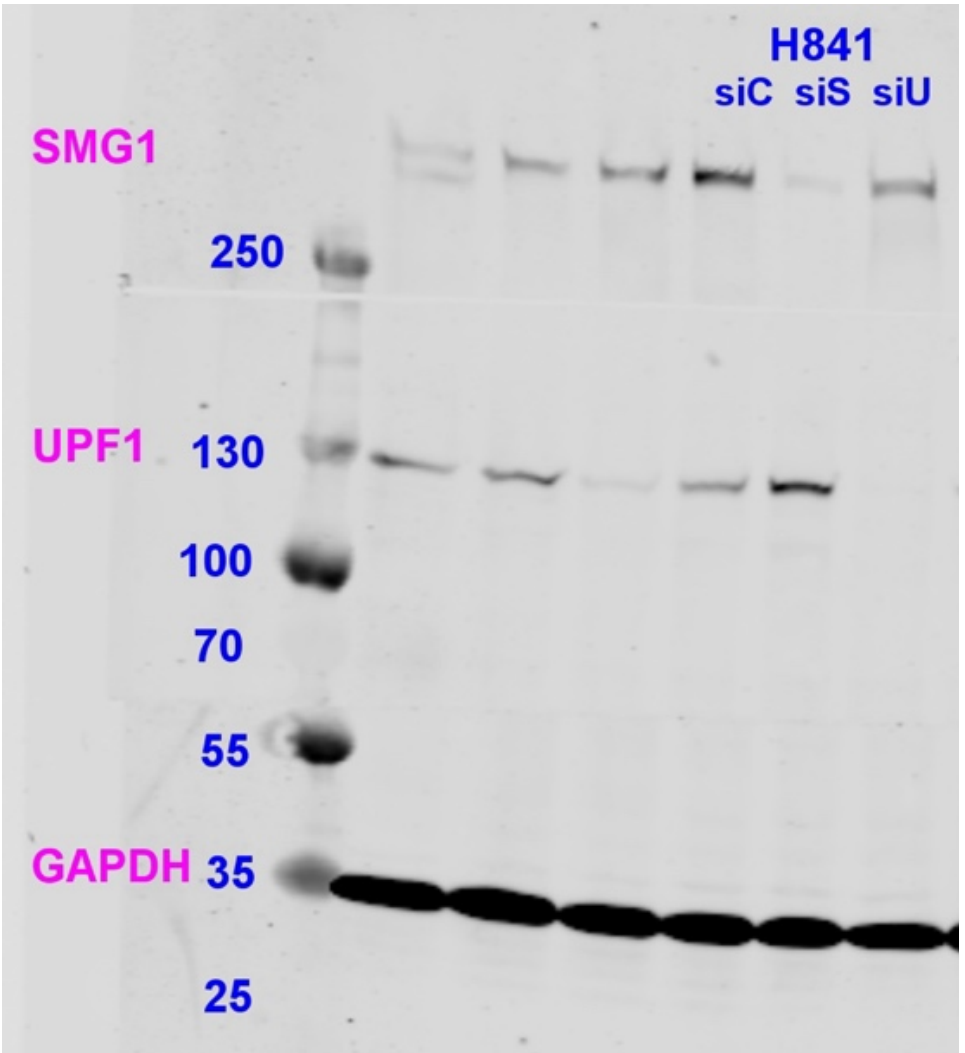

Figure S2B_H1048

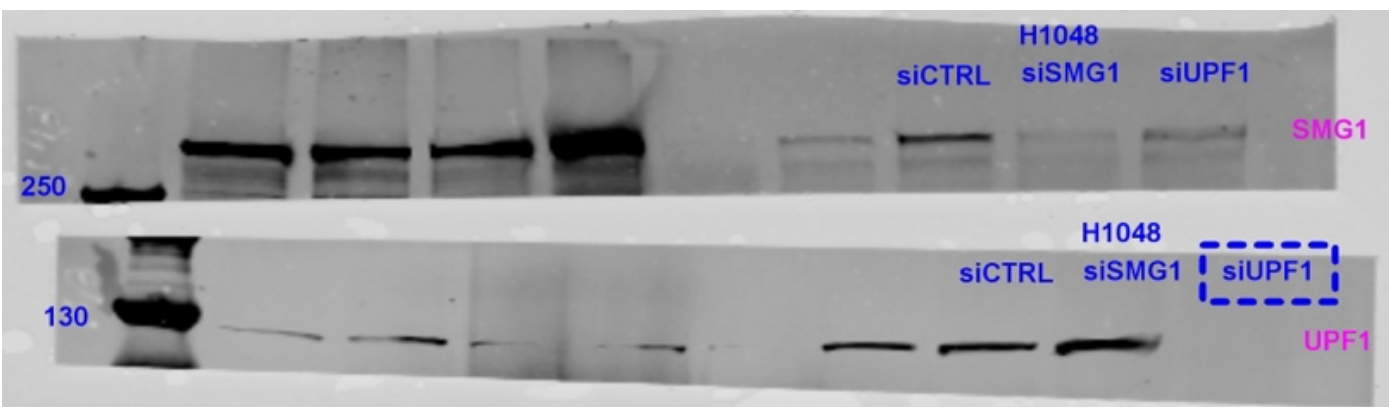

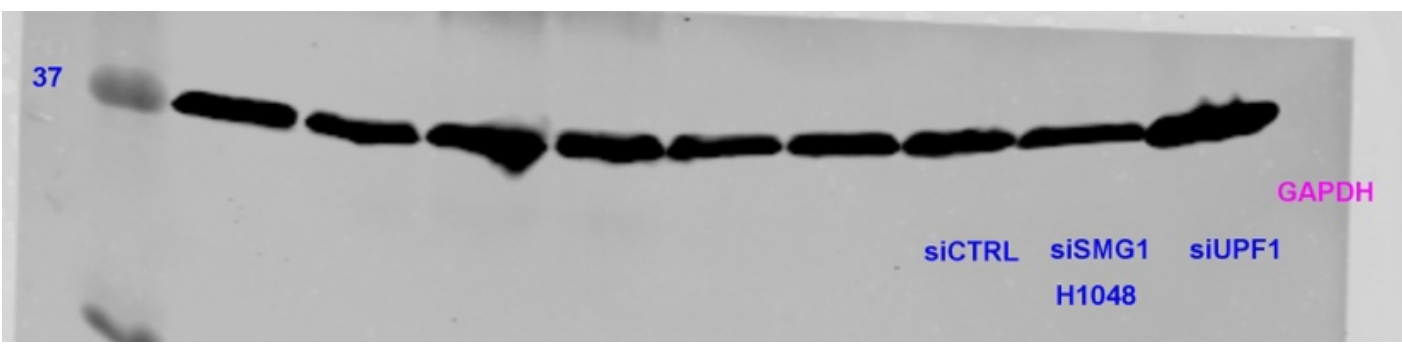

Figure S2B_H1341

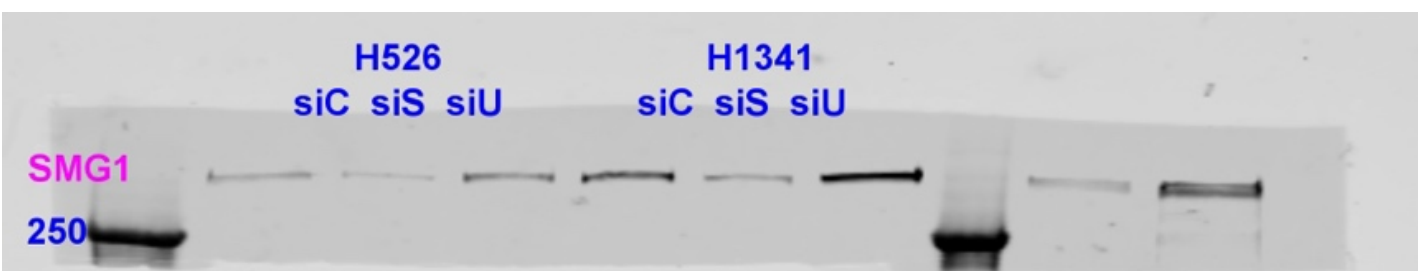

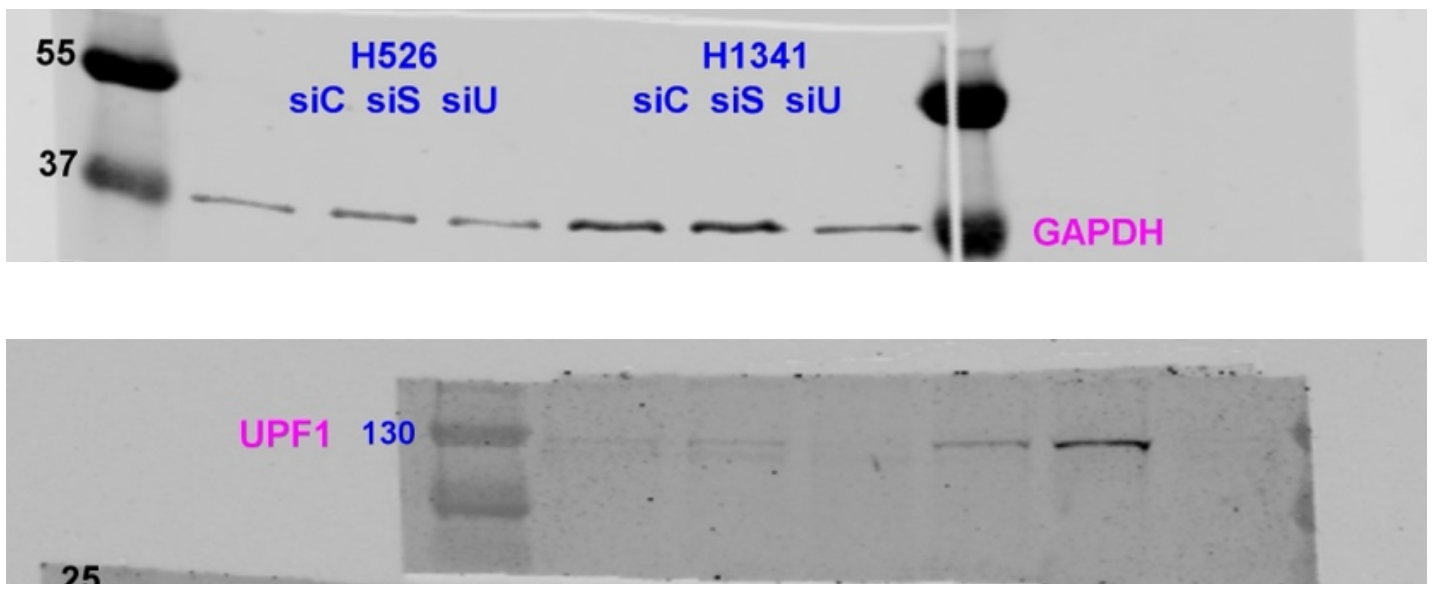

Figure S2B_GLC-1

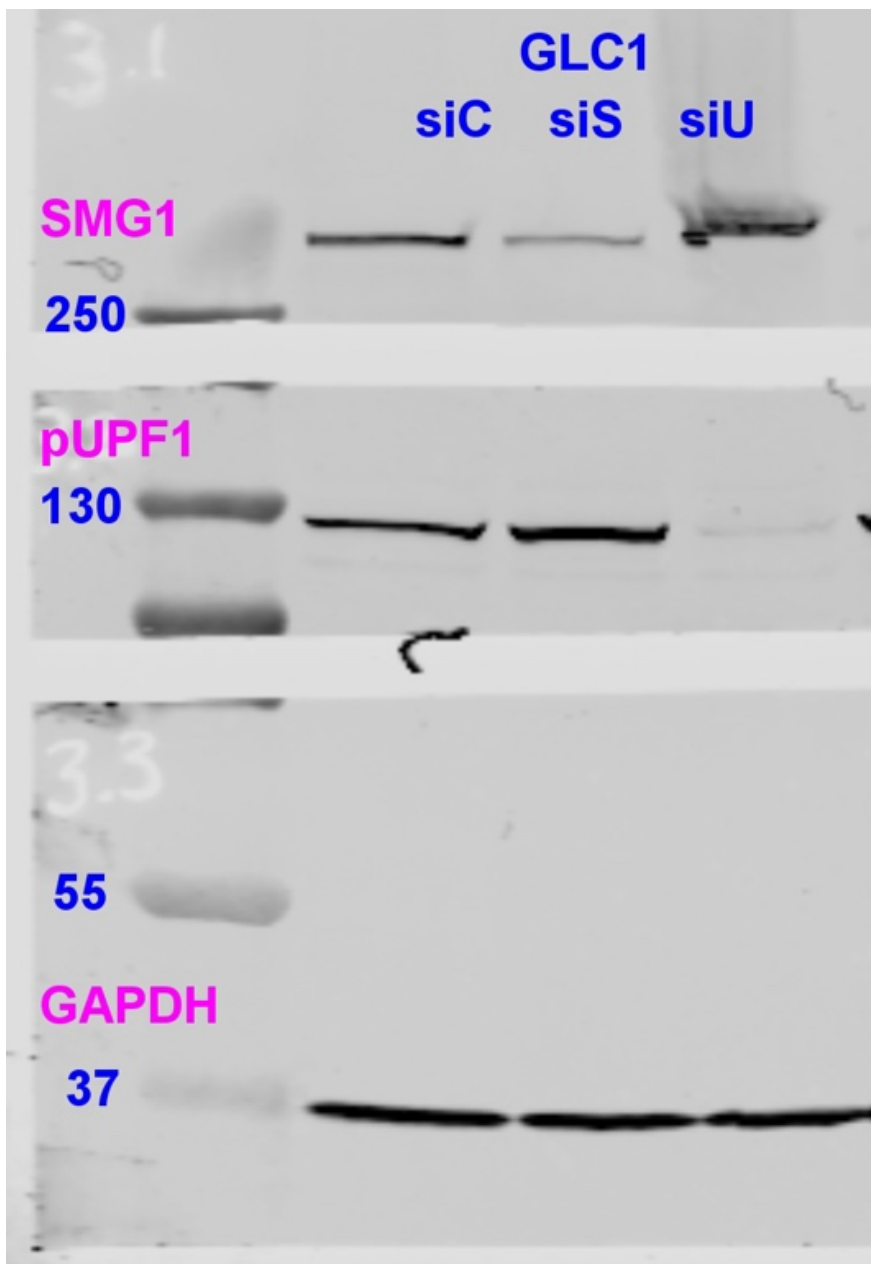

Figure S2B_N417

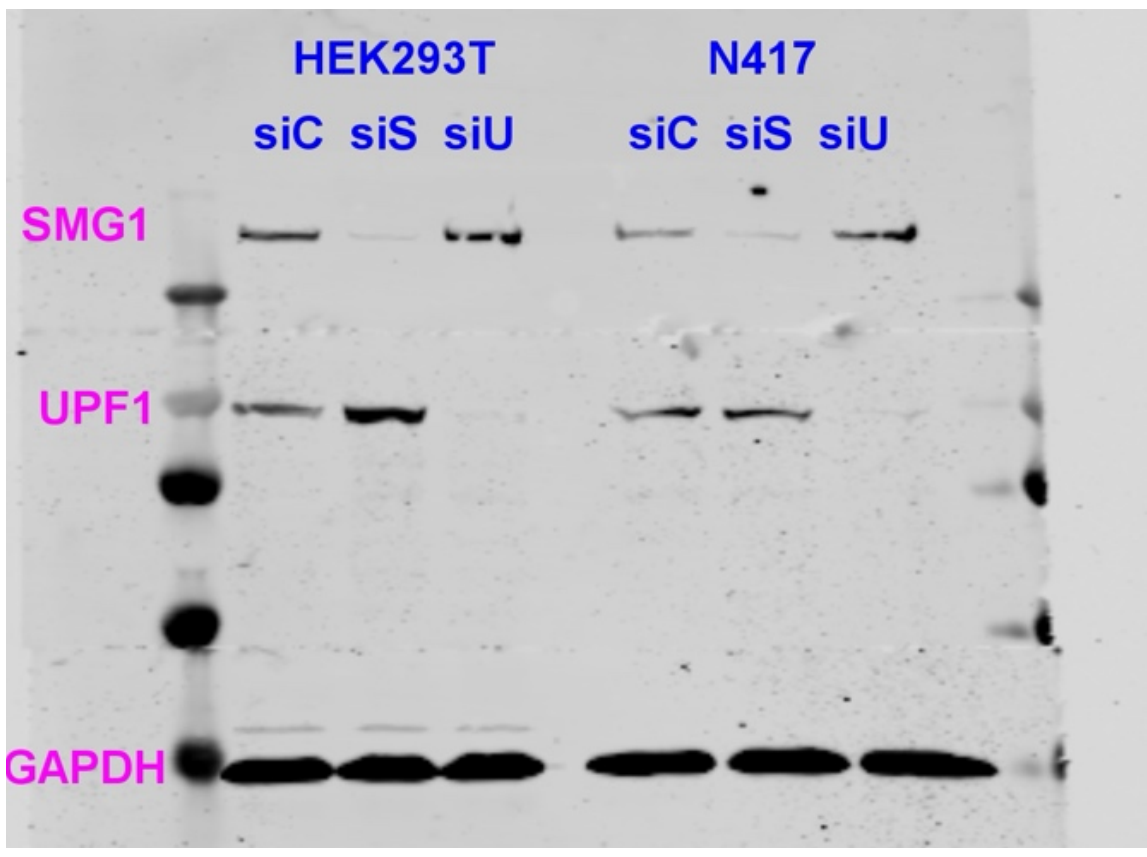

Figure S2D_GLC-1, H146, H1048, H1341, H841, N417

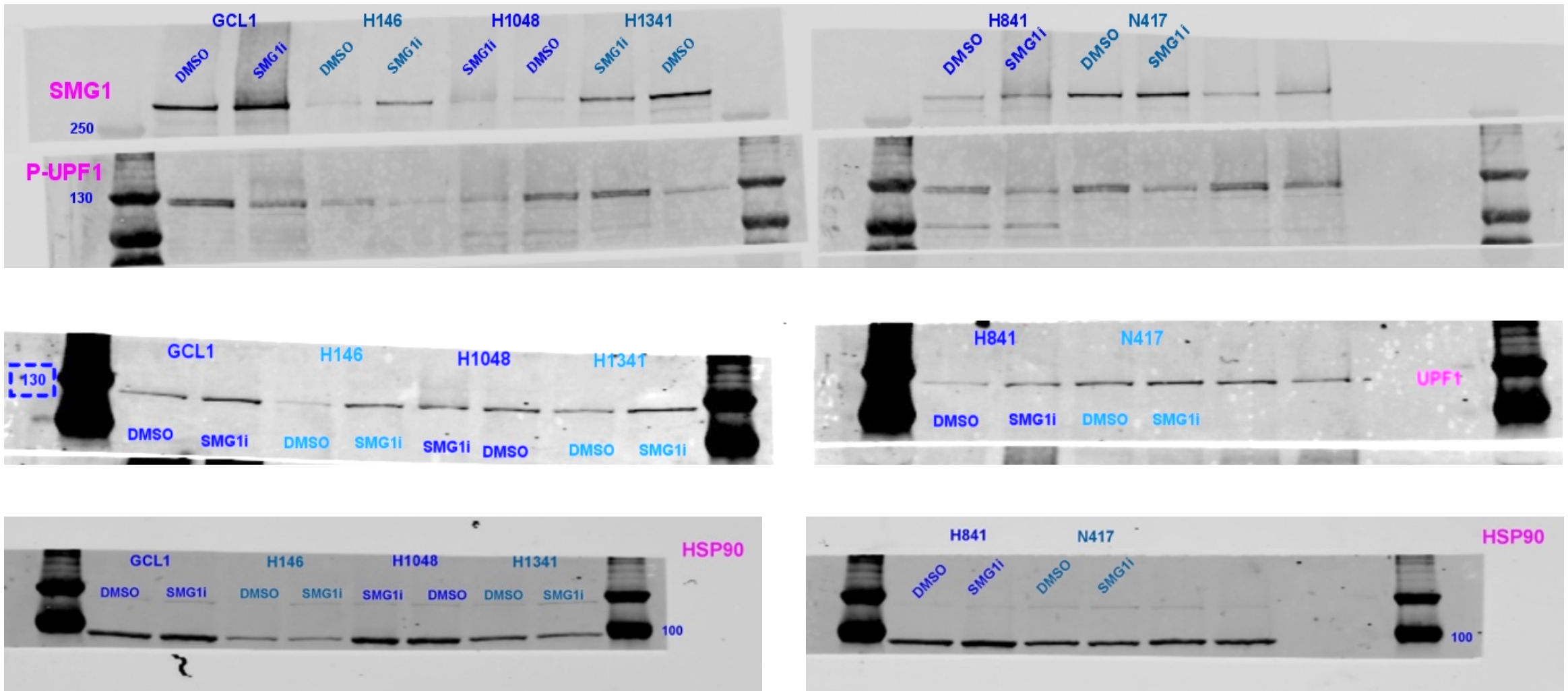

Figure S2D_H1694 & Figure S2K_RP1380

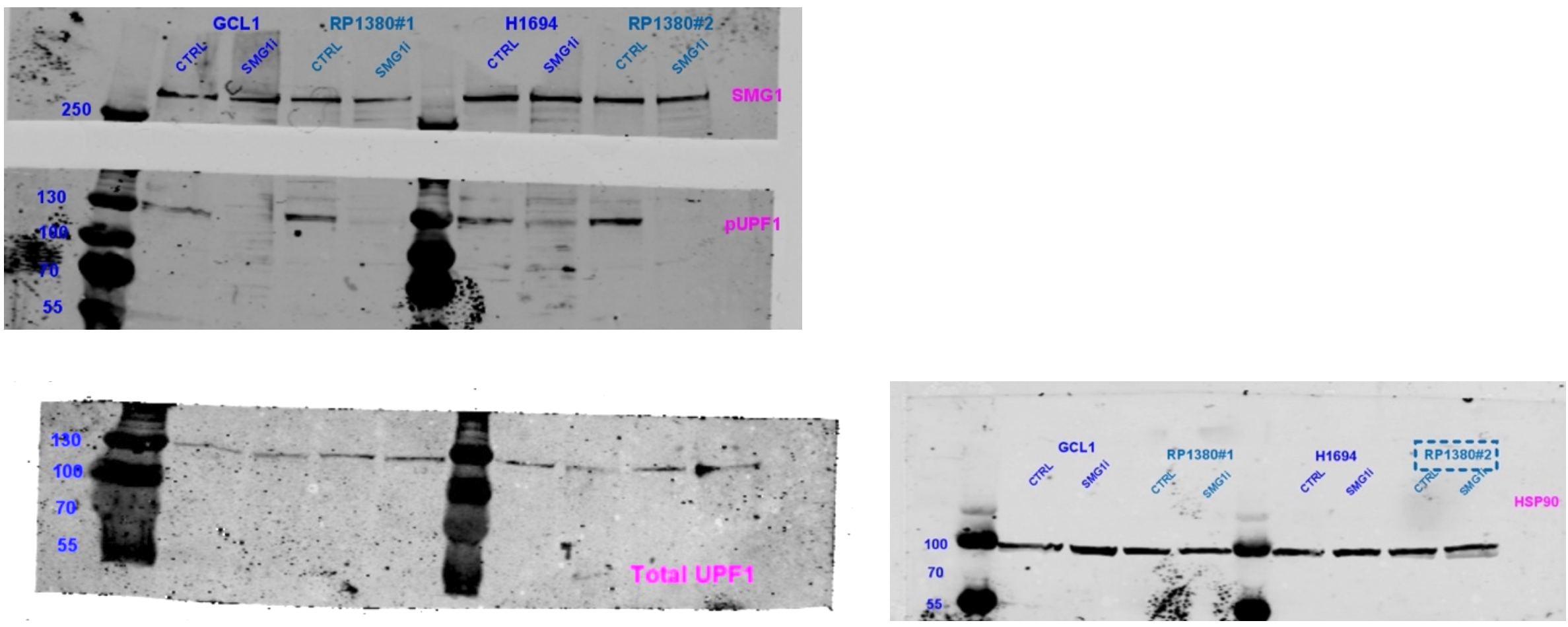

Figure S2K_RPM111

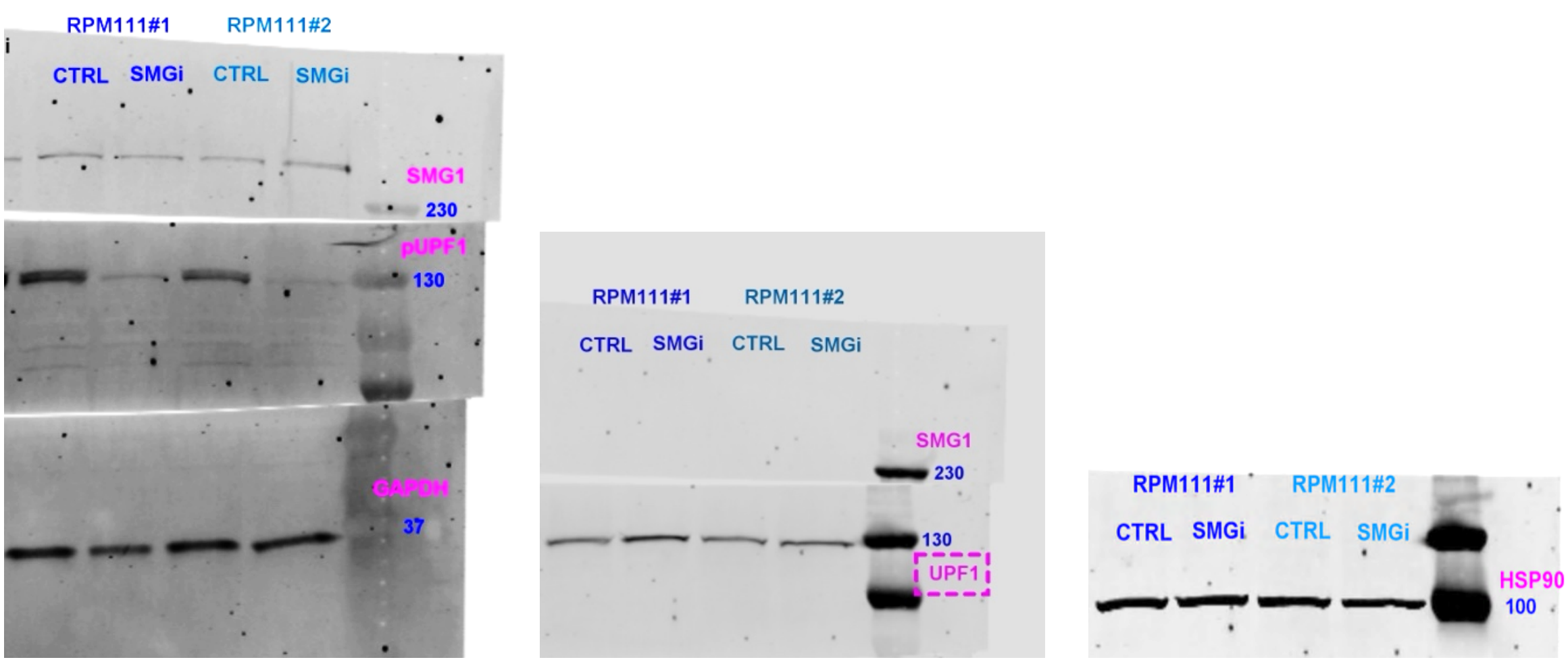

Figure S3C_UPF1, AKT, 4EBP

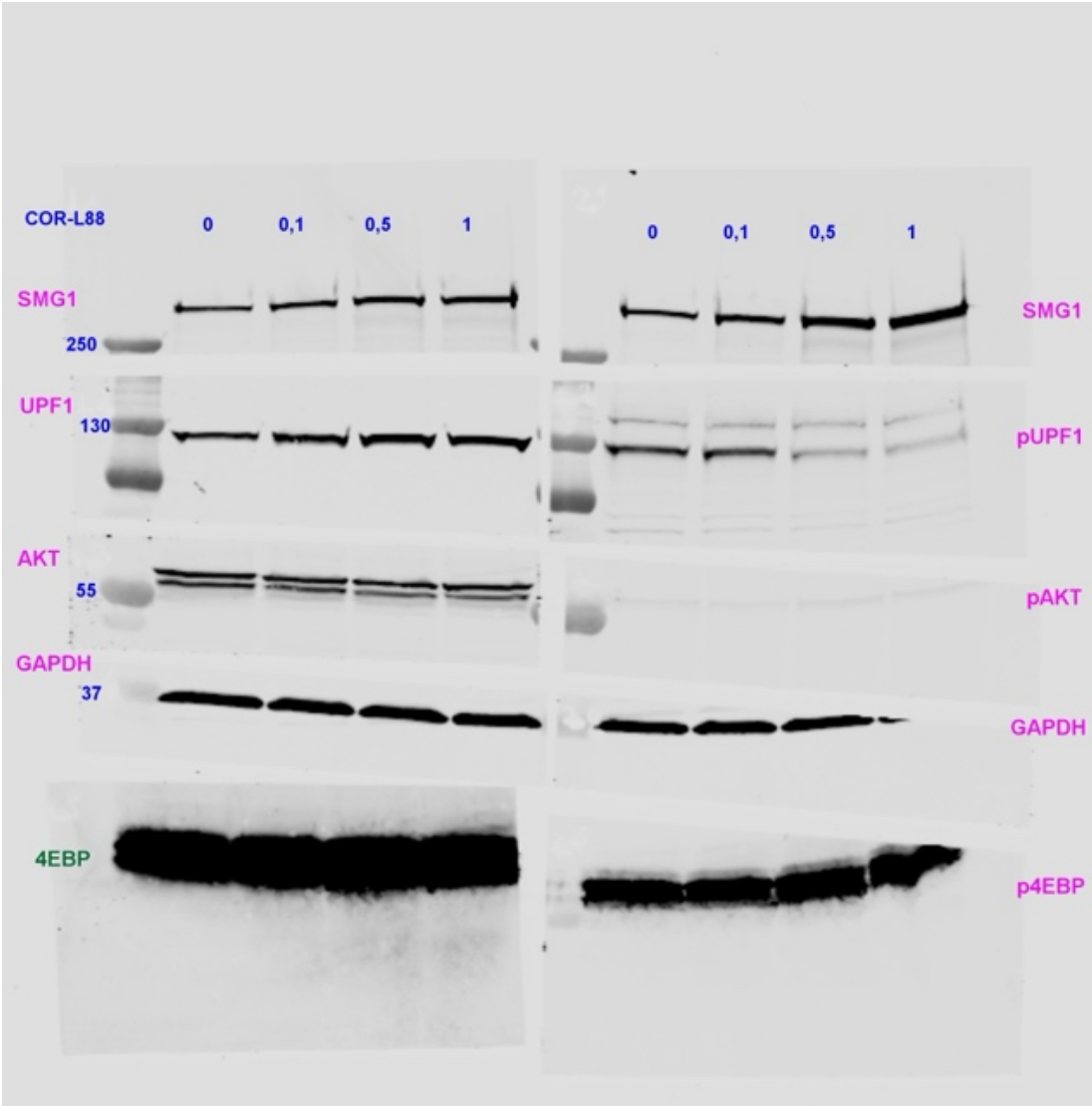

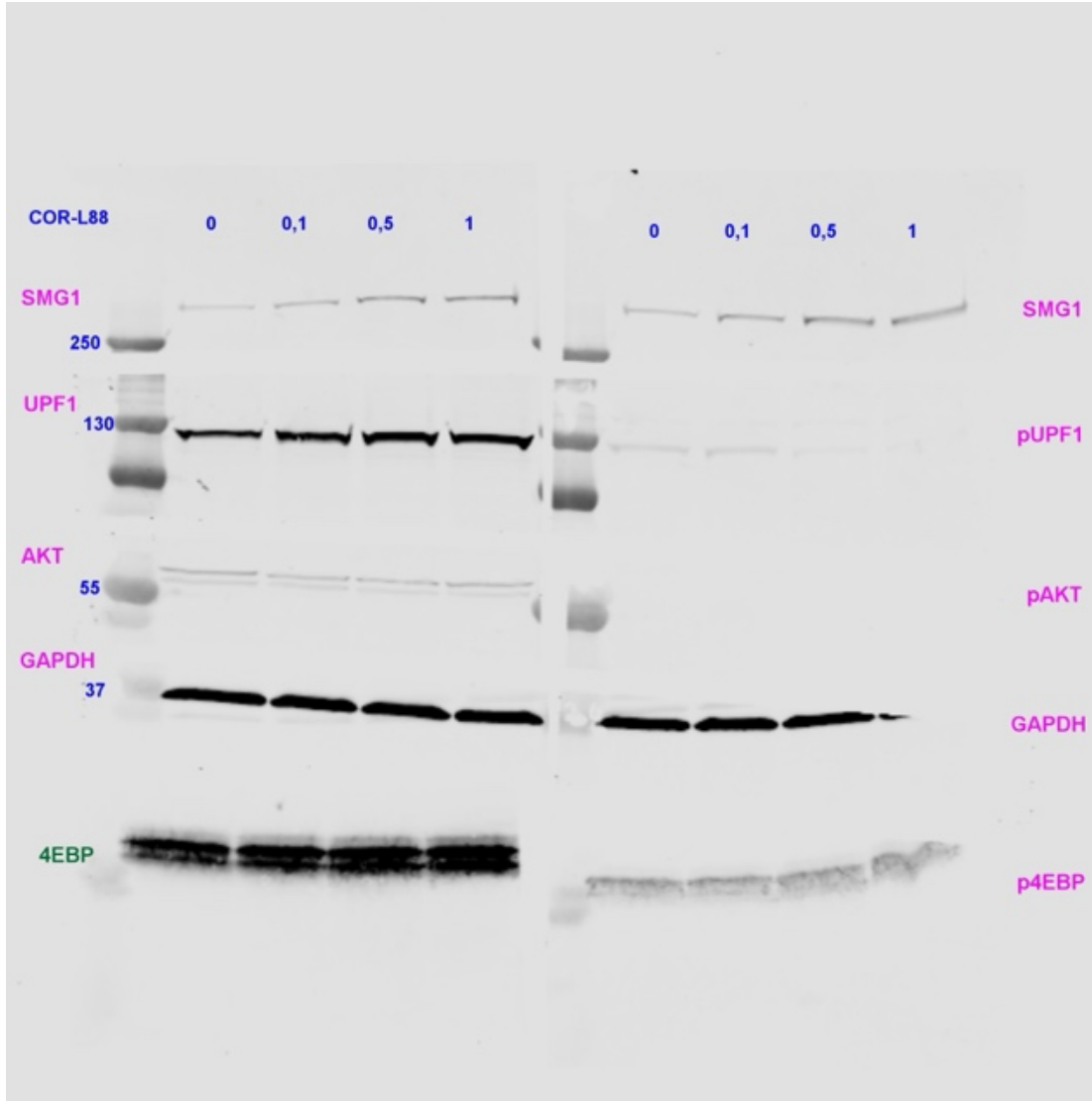

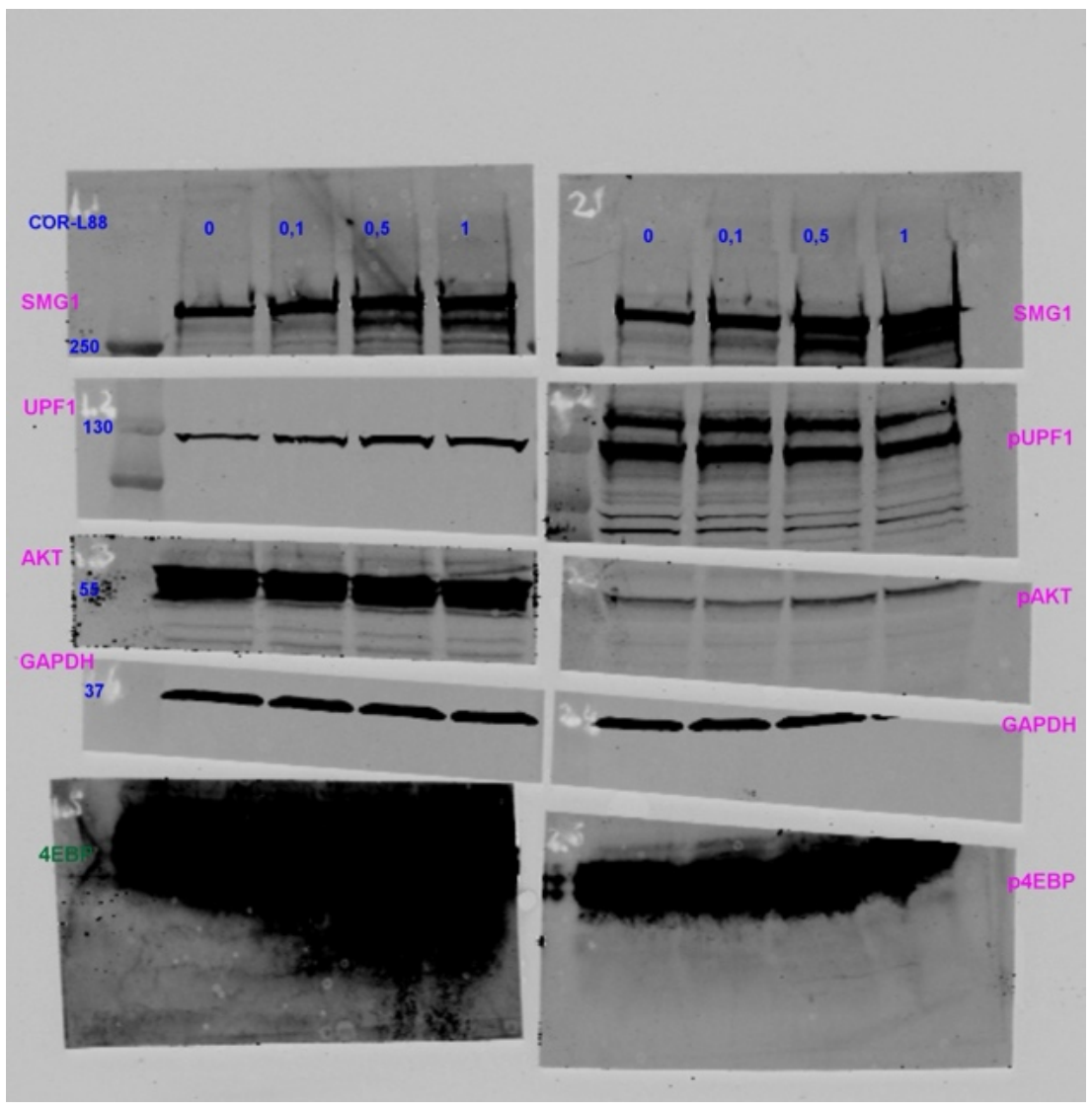

Figure S3C_STAT3

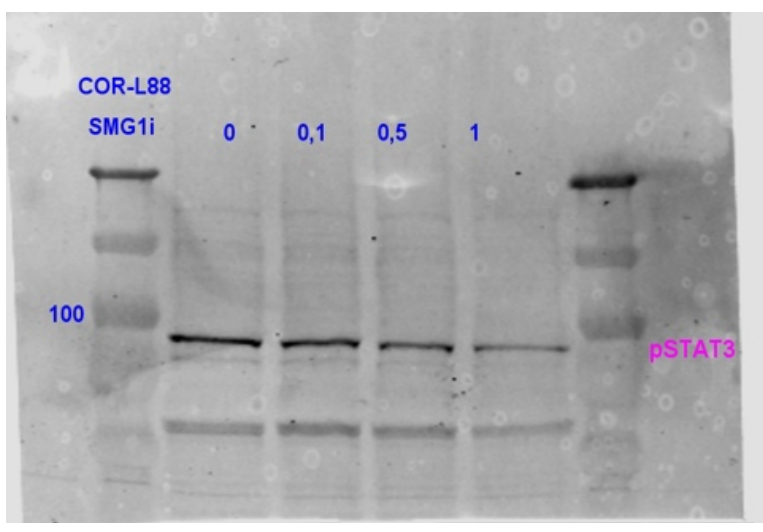

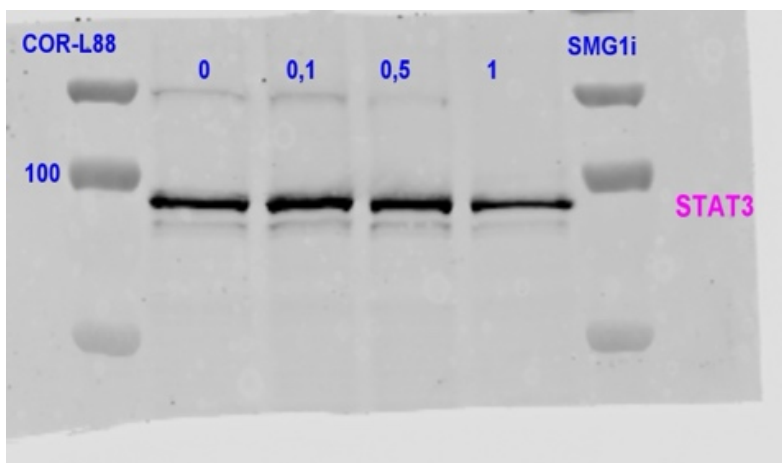

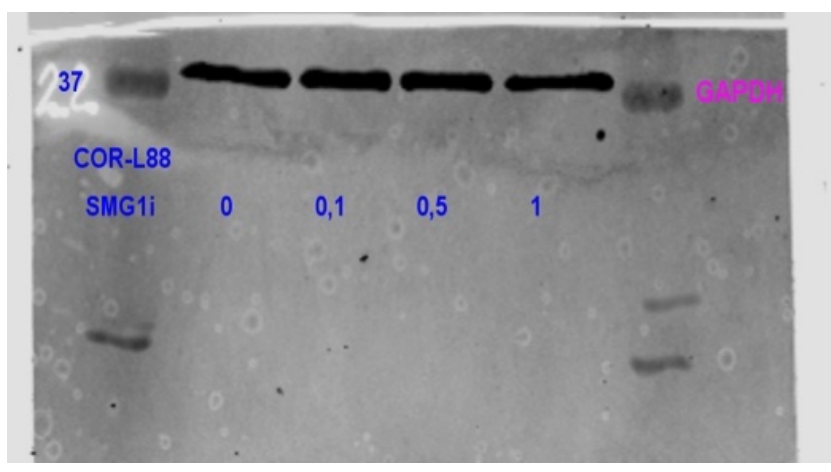

Figure S7G

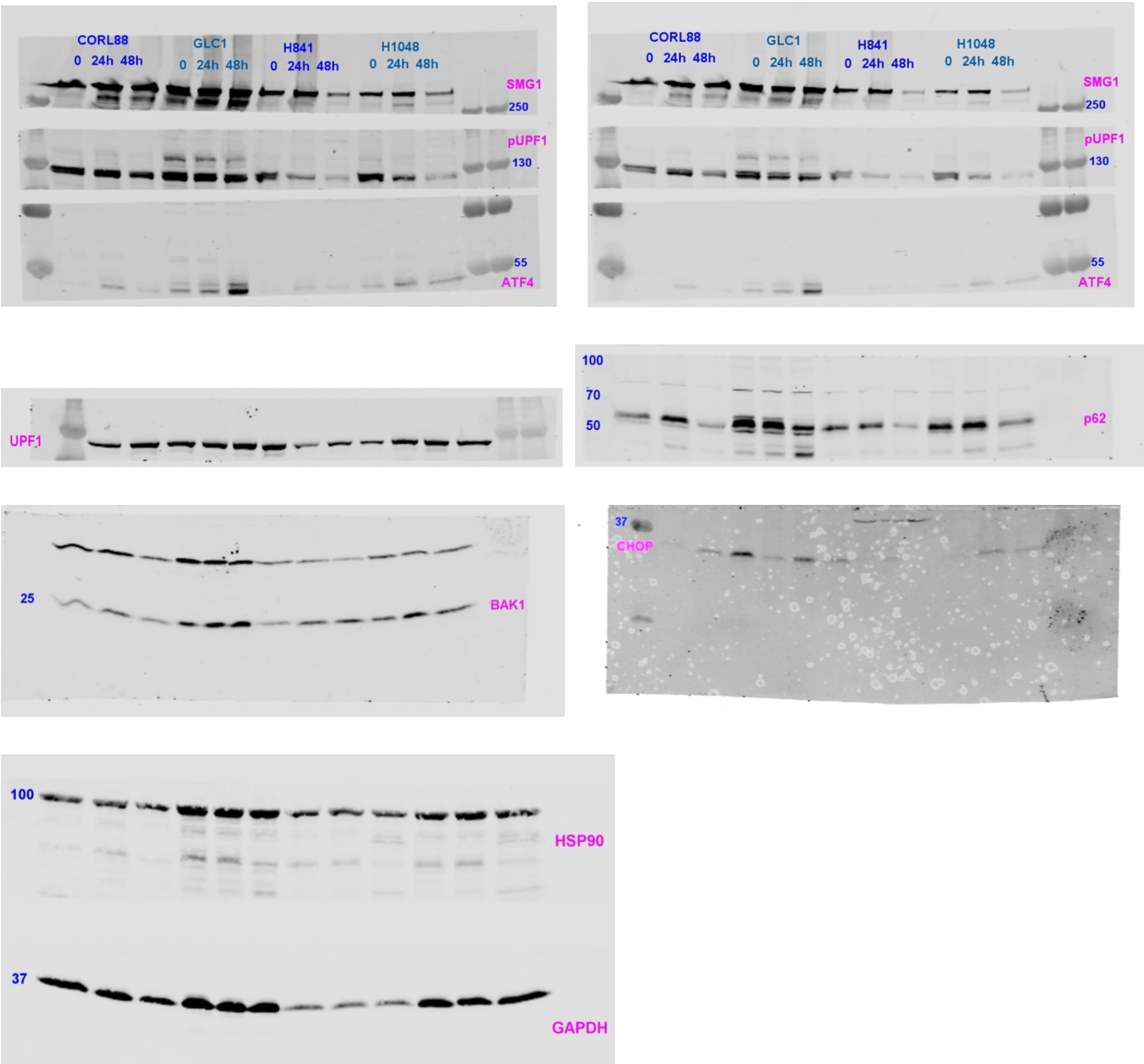

Figure S8A

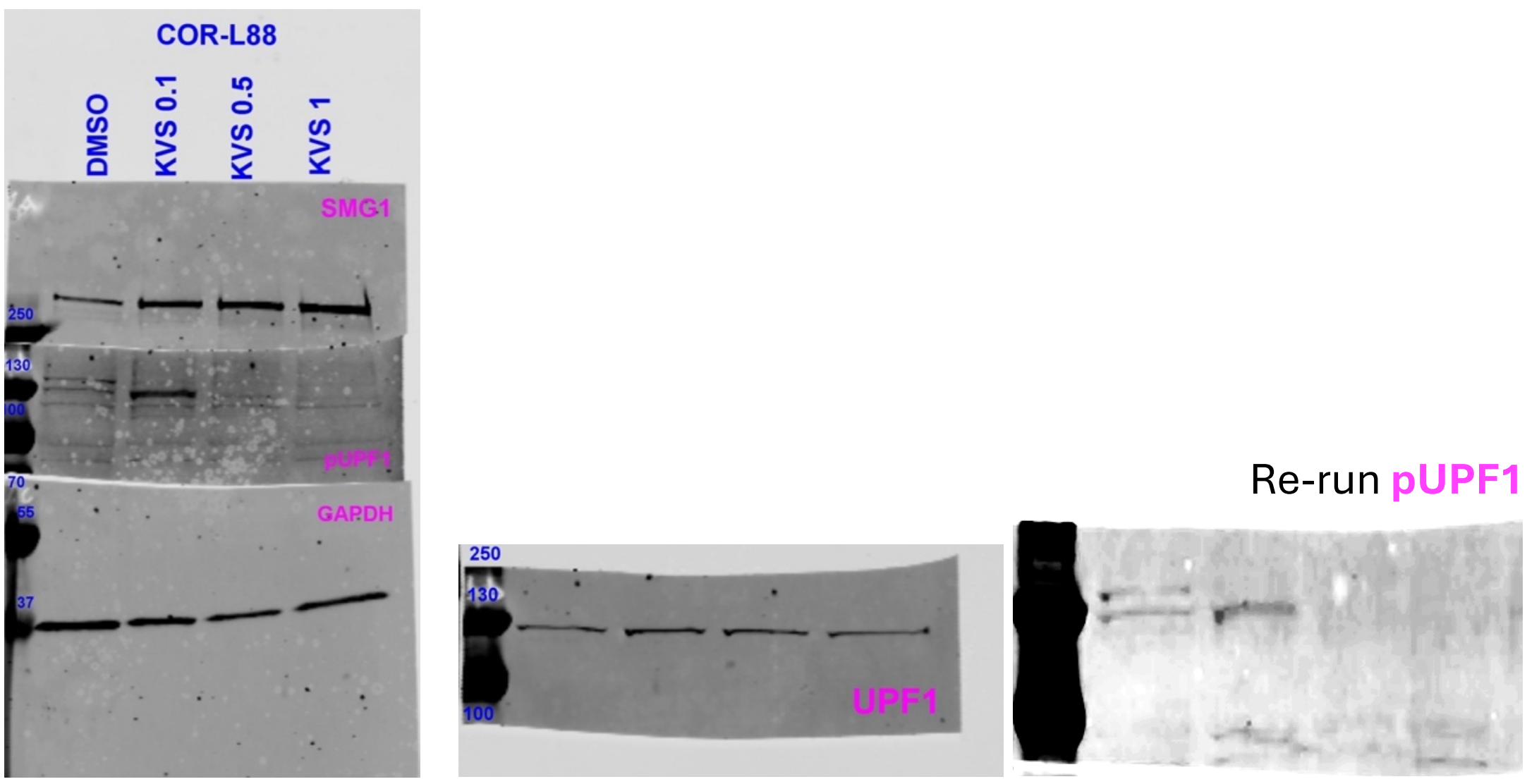

Figure S8D_RP1380

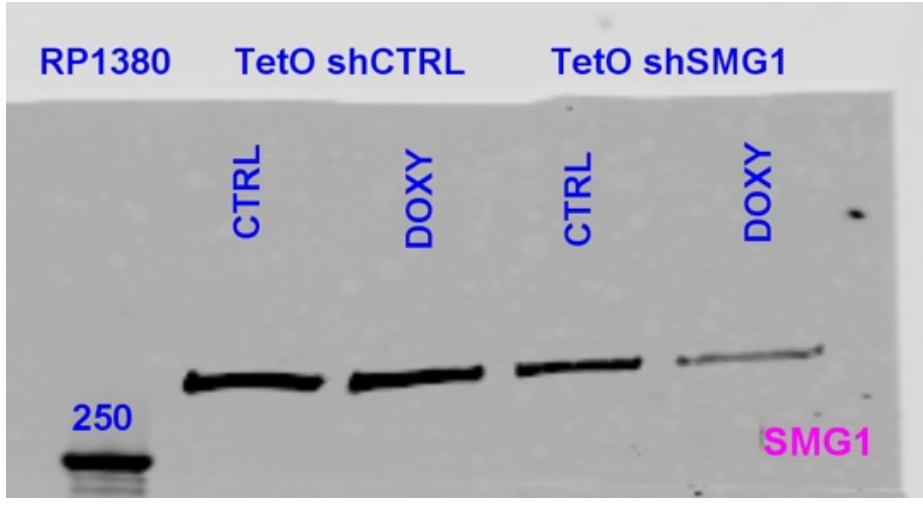

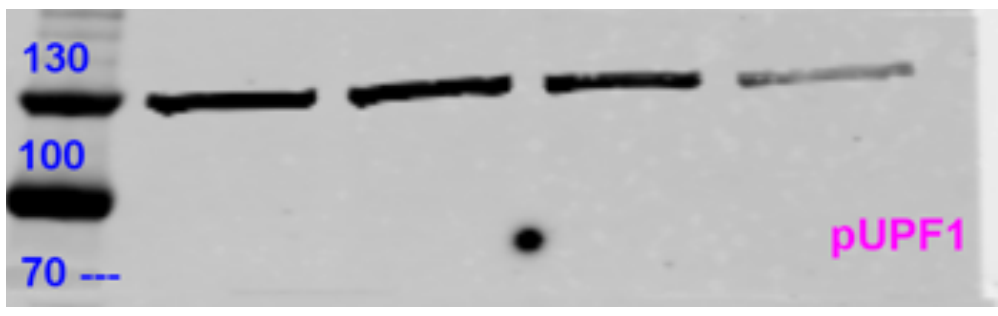

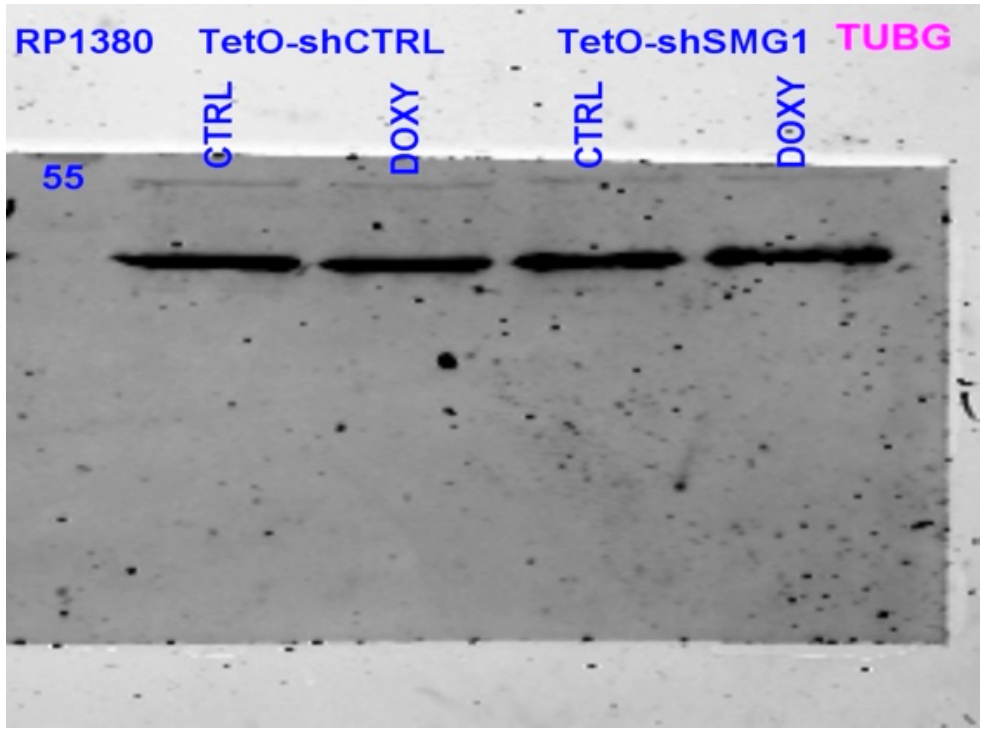

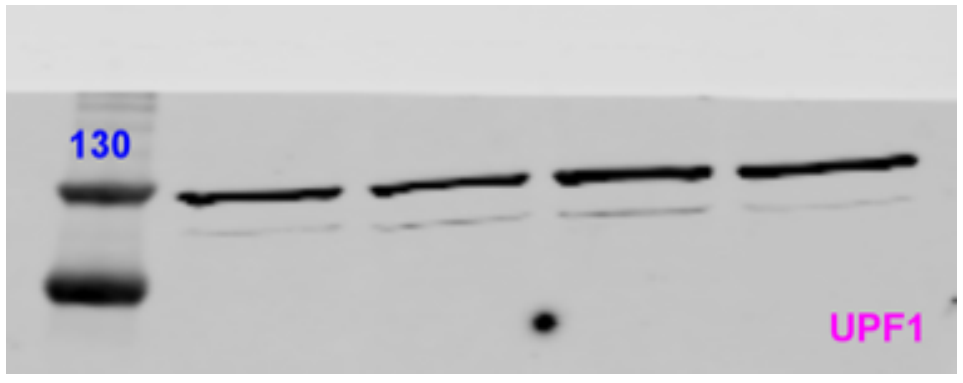

Figure S8D_H841

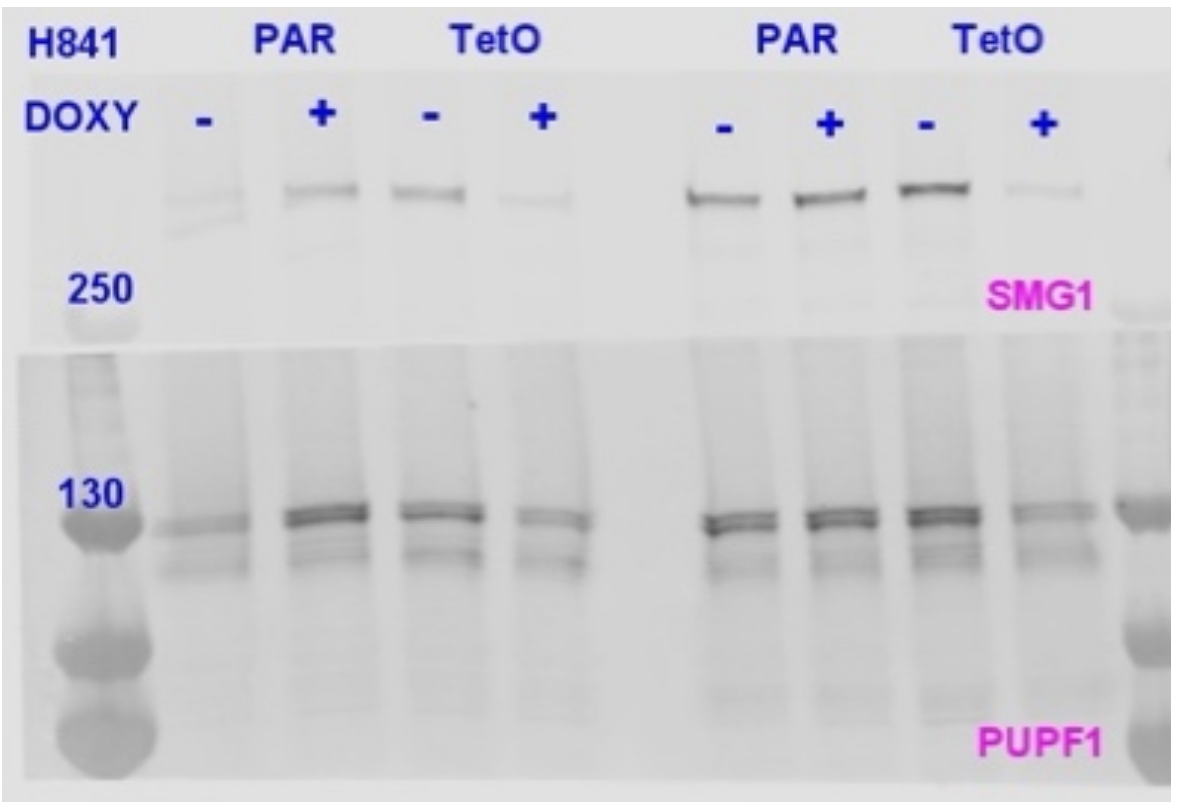

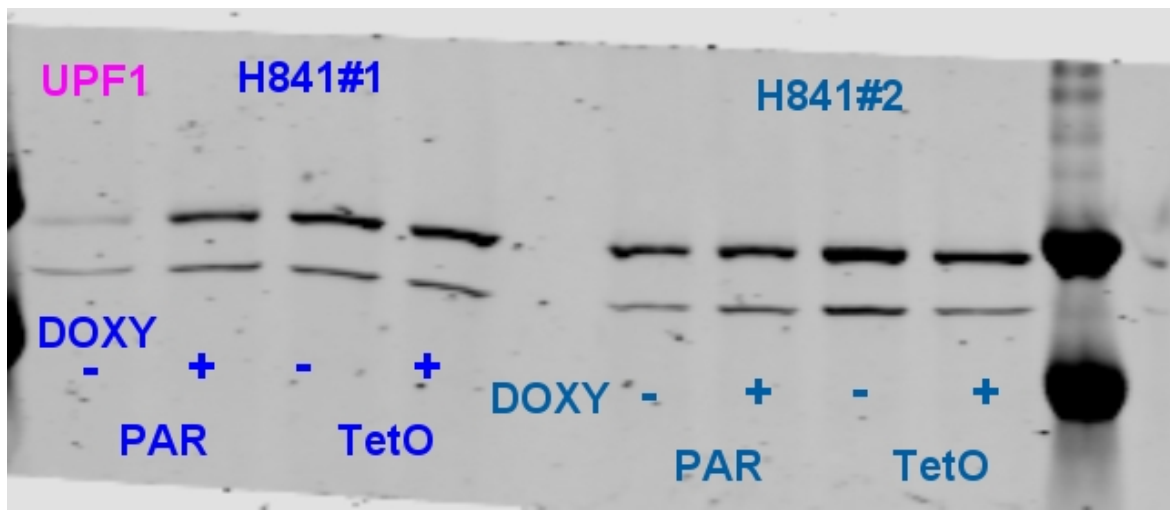

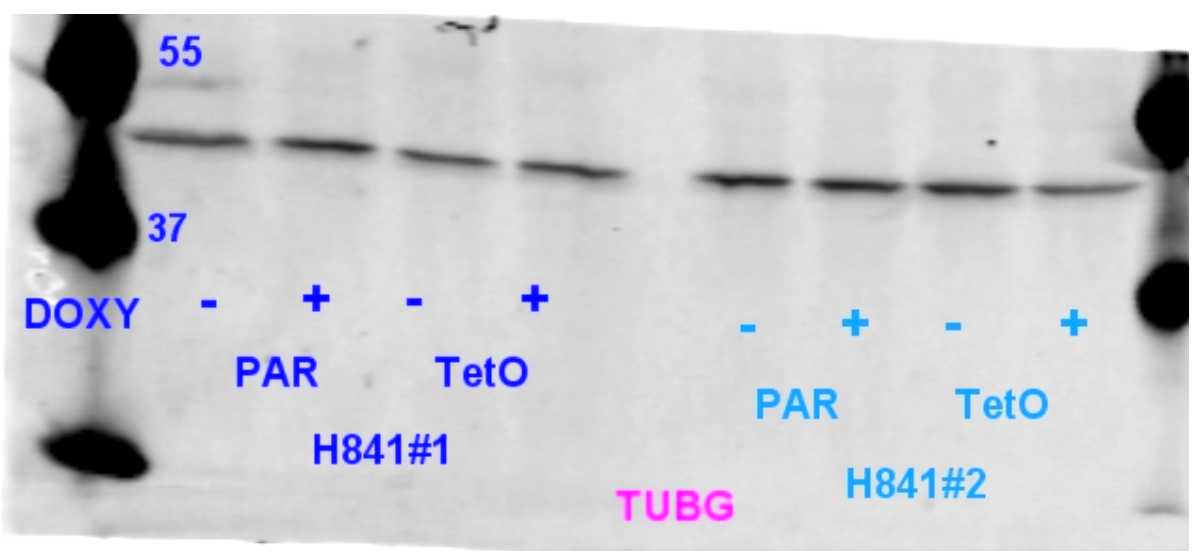

Figure S8D_GLC-1

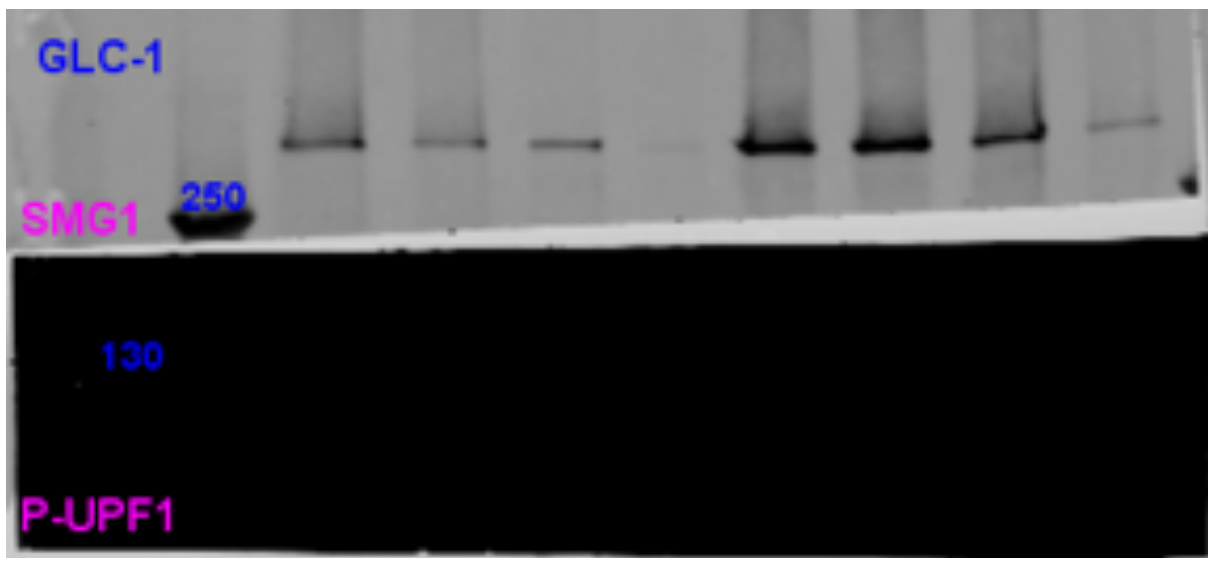

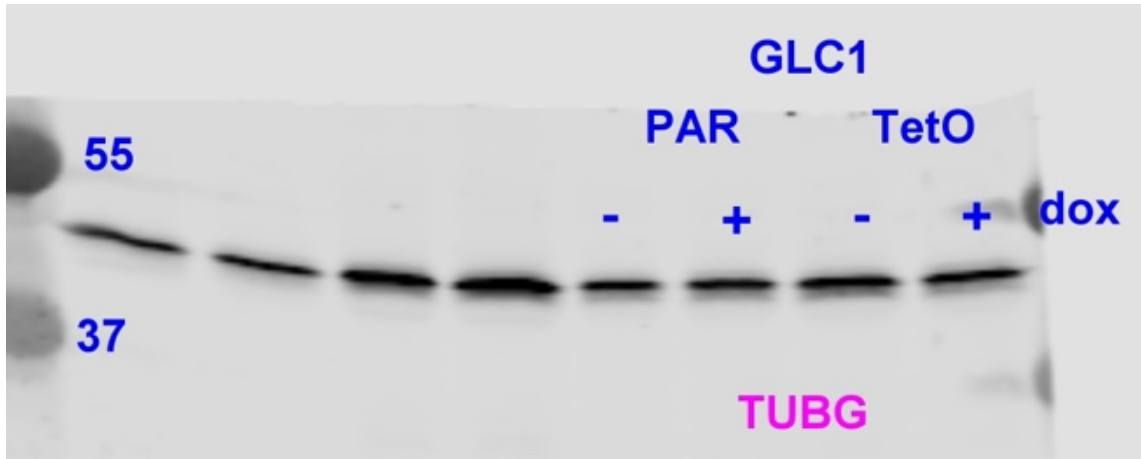

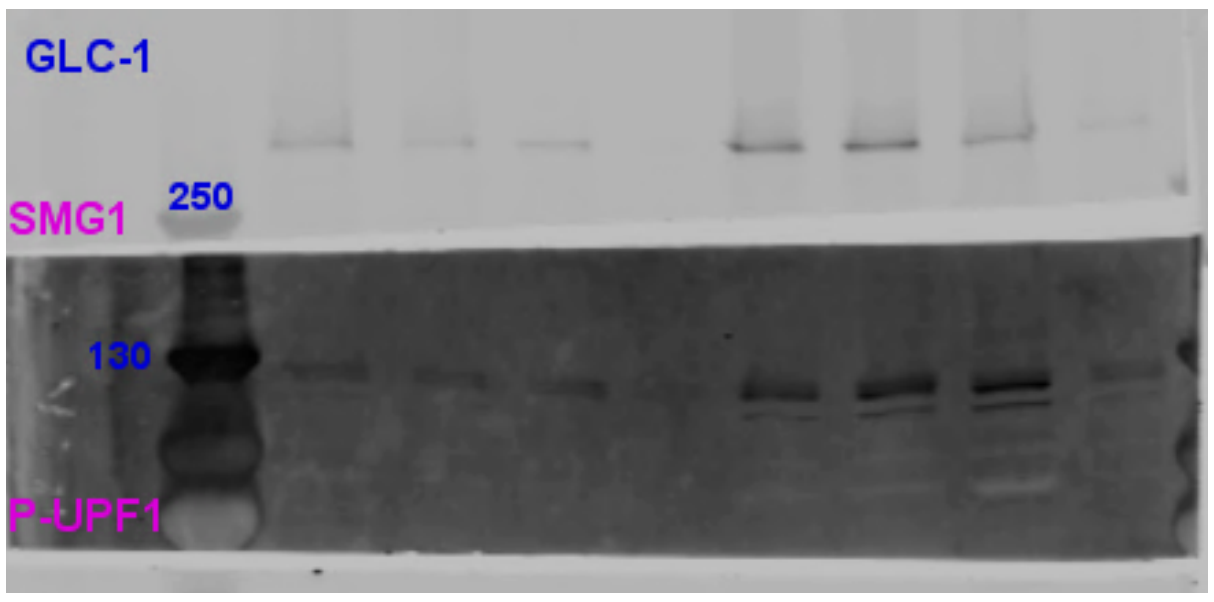

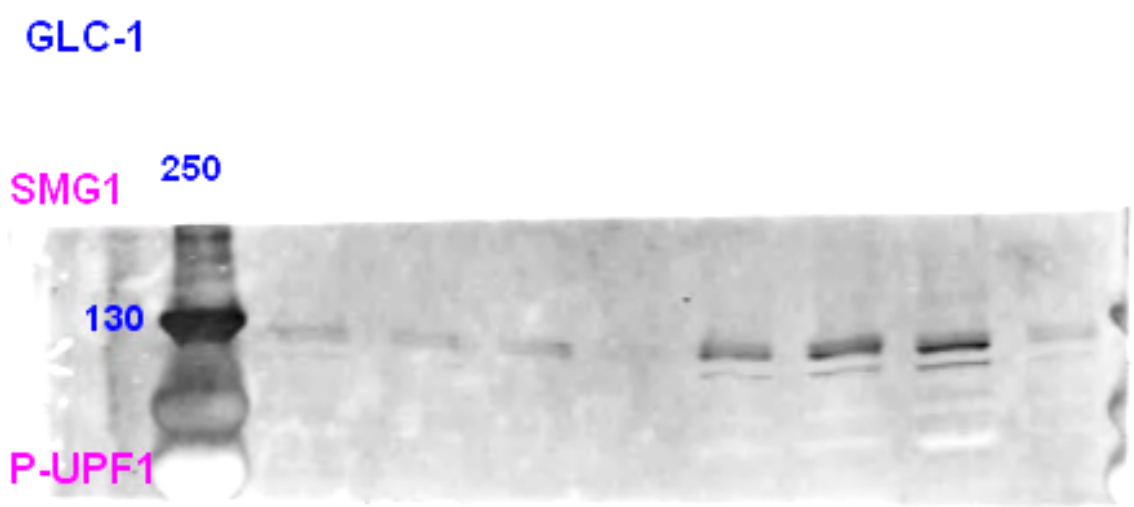

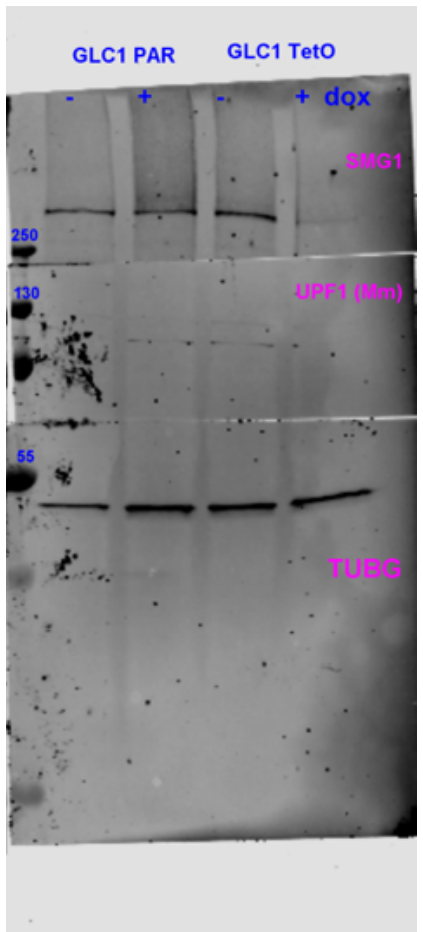

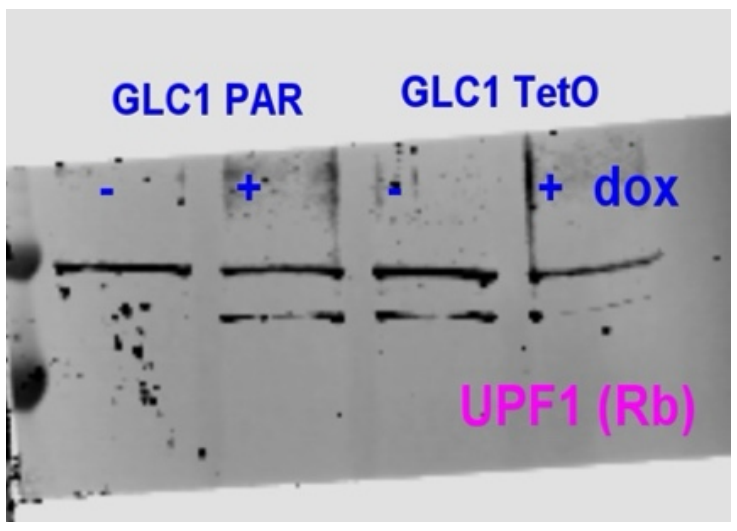

Blot re-run to detect total UPF1

Figure S8D_H1048

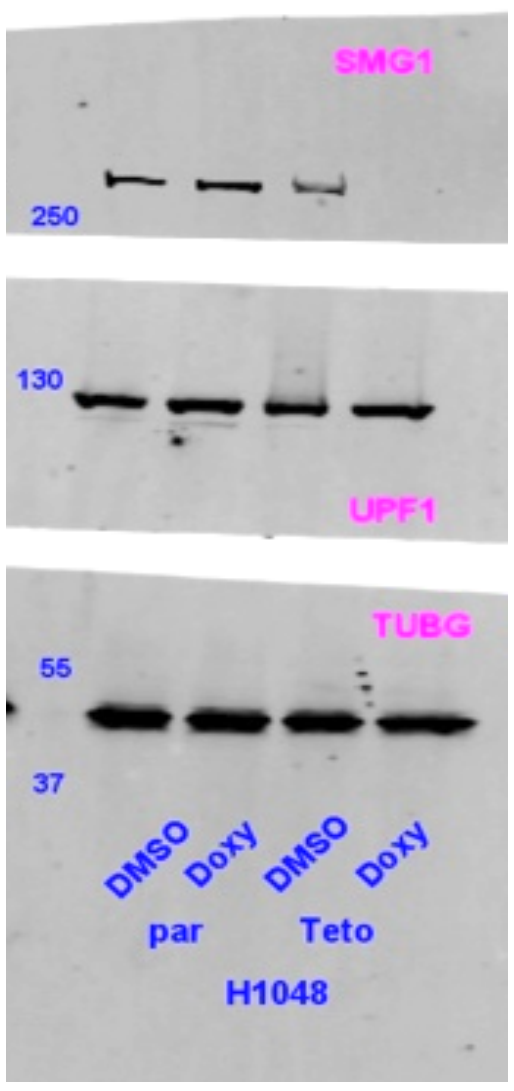

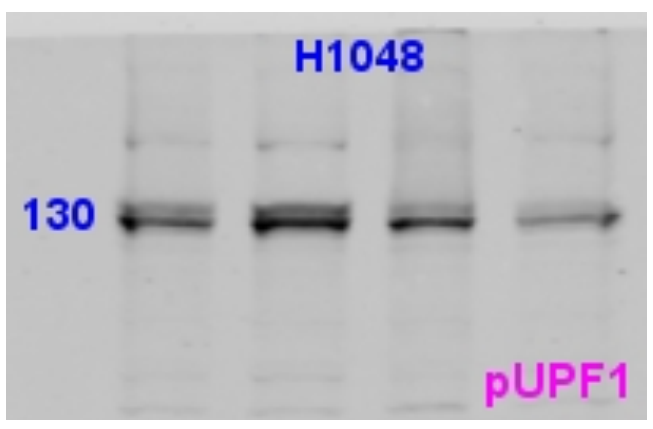

Figure 8H

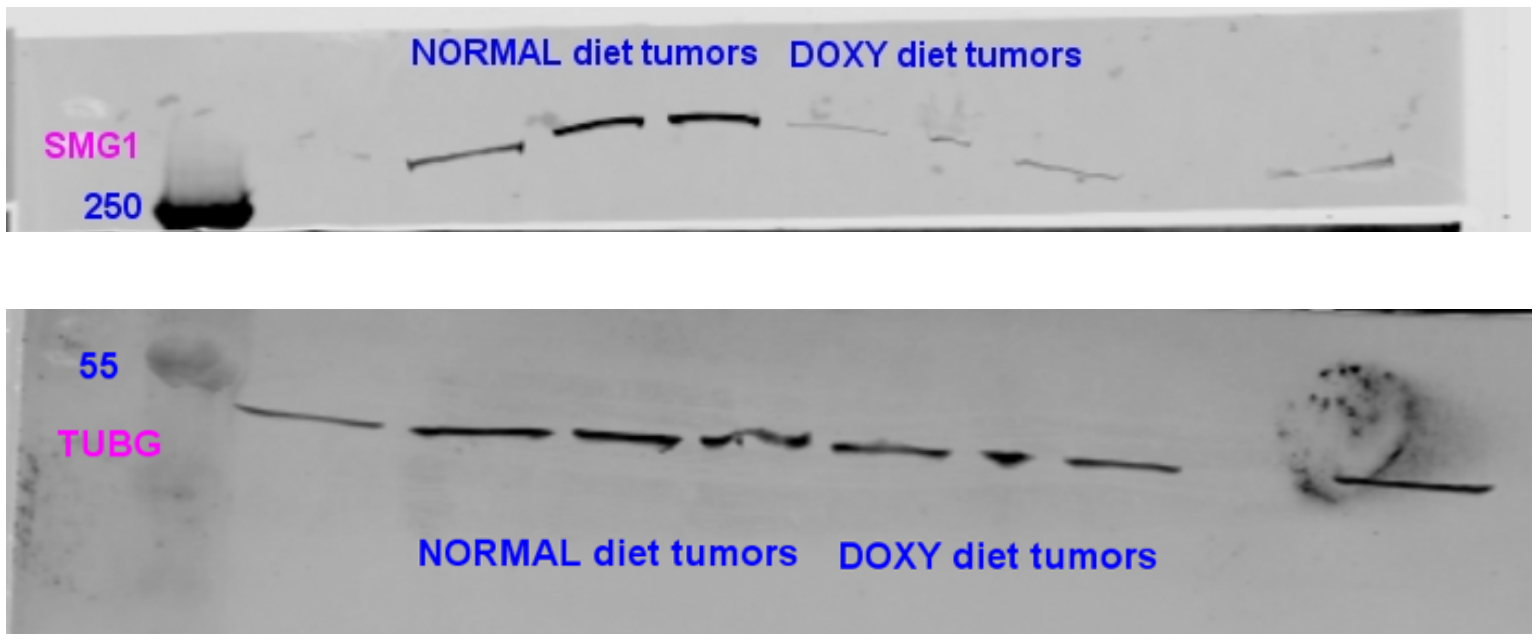

Figure S13C

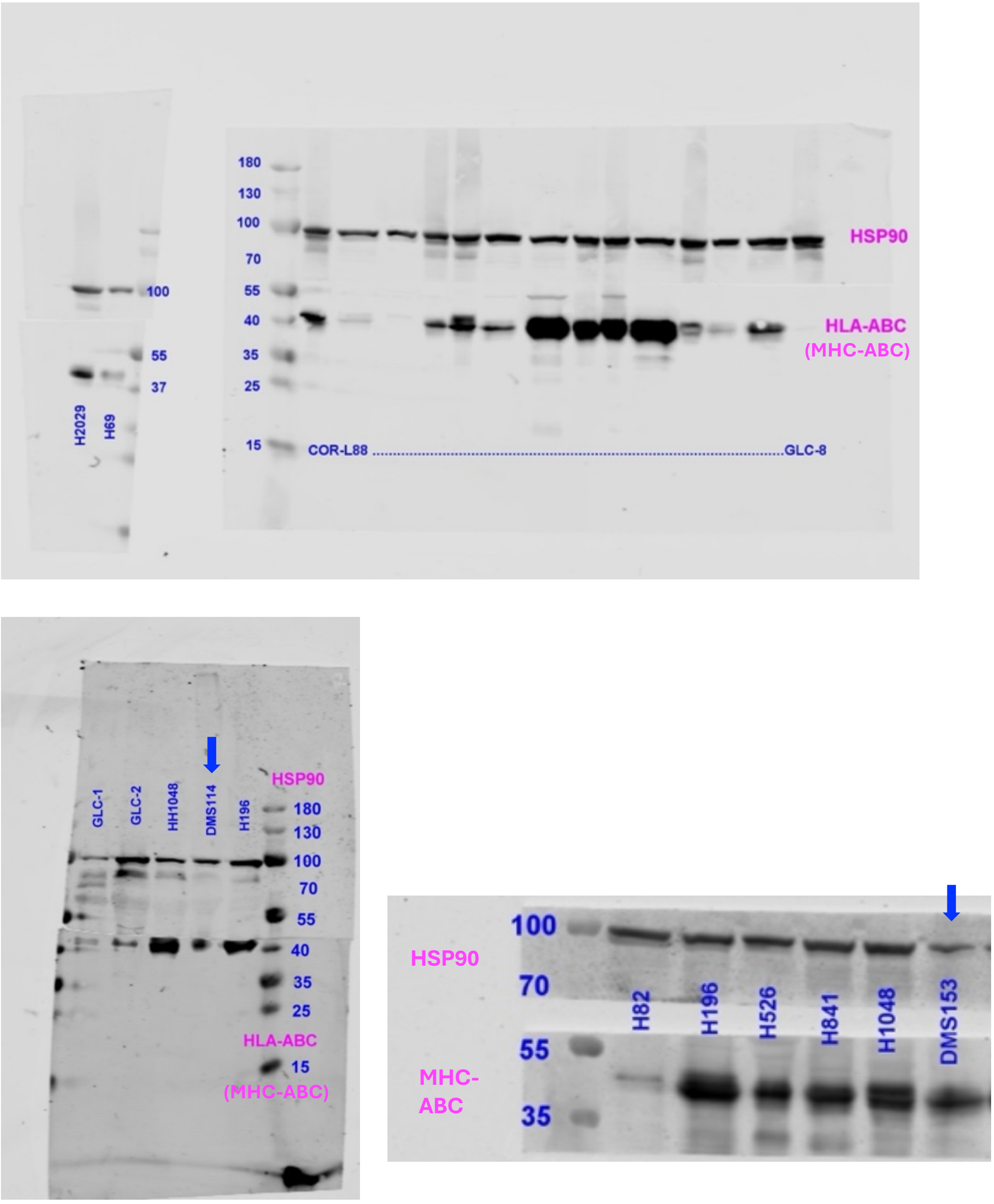

Supplement: Supplementary file 2 [file 12943_2026_2750_MOESM2_ESM.pdf]
